# Supplementary material for: Are there lost opportunities in chronic kidney disease? A region-wide cohort study
Source: BMJ Open. 2024 Apr 19;14(4):e074064. doi: 10.1136/bmjopen-2023-074064 (PMC11033666; doi:10.1136/bmjopen-2023-074064)
Supplement: Supplementary data [file bmjopen-2023-074064supp001.pdf]

BMJ Publishing Group Limited (BMJ) disclaims all liability and responsibility arising from any reliance placed on this supplemental material which has been supplied by the author(s)

Lost opportunities in chronic kidney disease

Supplemental Material

Contents

Supplemental Methods. The CELOSIA database 2

Table S1. Meta-analyses that assessed the effect of first-line pharmacological treatments on the risk of major adverse cardiovascular events in patients with chronic kidney disease, with or without diabetes 3

Table S2. Inclusion criteria for the CELOSIA database from which the study sample was extracted 4

Table S3. Stages of chronic kidney disease diagnosed using measurements of eGFR and UACR according to the KDIGO clinical practice guidelines 6

Table S4. International Classification of Diseases (ICD)-10 codes used to search for diagnoses of CKD, prevalent comorbidities, and outcomes of interest 7

Table S5. Anatomical Therapeutic Chemical (ATC) classification system codes used to search for treatments of interest in this study's population 8

Table S6. Baseline characteristics of the total study cohort stratified by age group 9

Table S7. Proportions of patients diagnosed with chronic kidney disease in healthcare within five years of the development of the disease 11

Figure S1. Participant flow 12

Figure S2. Multi-state models presenting the proportions of patients diagnosed or not diagnosed with chronic kidney disease (CKD) in healthcare who received or didn't receive guideline-directed pharmacological therapy, including the terminal, absorbing outcome, all-cause death 13

Figure S3. Multi-state models presenting the proportions of patients diagnosed or not diagnosed with chronic kidney disease (CKD) in healthcare who received or didn't receive guideline-directed pharmacological therapy, including the terminal, absorbing outcomes, major adverse cardiovascular events (MACE) and non-cardiovascular disease (CVD) death 14

Figure S4. Multi-state models presenting the proportions of patients diagnosed or not diagnosed with chronic kidney disease (CKD) in healthcare who received or didn't receive guideline-directed pharmacological therapy, including the terminal, absorbing outcome, all-cause death 15

Figure S5. Multi-state models presenting the proportions of patients diagnosed or not diagnosed with chronic kidney disease (CKD) in healthcare who received or didn't receive guideline-directed pharmacological therapy, including the terminal, absorbing outcomes, major adverse cardiovascular events (MACE) and non-cardiovascular disease (CVD) death 16

References 17

Sundström J, *et al. BMJ Open* 2024; 14:e074064. doi: 10.1136/bmjopen-2023-074064

Supplemental Methods. The CELOSIA database

The CELOSIA database, from which this present study's sample was extracted, includes all Swedish residents aged ≥18 years who were diagnosed with chronic kidney disease (CKD), heart failure or diabetes mellitus between January 1st, 2000, and December 31st, 2020.

Patients were identified from several sources with national coverage, including the Swedish Prescribed Drug Registry, the National Patient Registry, and the National Death Registry. The Swedish Prescribed Drug Registry logs each resident's filled drug prescriptions according to the Anatomical Therapeutic Chemical [ATC] classification system.[1] The National Patient Registry and the National Death Registry records diagnoses, surgical procedures, and causes of death using the International Classification of Diseases [ICD][2] system and the Nordic Medico-Statistical Committee Classification of Surgical Procedure system.[3]

Patients were also identified from several data sources with regional coverage, including the Healthcare Data Warehouse of Region Stockholm, the Healthcare Data Warehouse of Region Skåne Melior, and the Profdoc Medical Office of Region Skåne. Additionally, the CELOSIA database utilized electronic health records from Region Stockholm and Region Skåne to diagnose patients with CKD using laboratory measurements.

A complete list of the ATC codes, ICD-10 codes, procedure codes, and laboratory values used to identify these patients is available in Supplemental Table 2. Patients were required to have a 12-digit personal identity number, an identity number unique to all Swedish residents,[4] enabling data to be linked between the different sources.

The total population with data available for the present study was ~3.8 million people, corresponding to 36% of the Swedish population.

Sundström J, *et al. BMJ Open* 2024; 14:e074064. doi: 10.1136/bmjopen-2023-074064

Table S1. Meta-analyses that assessed the effect of first-line pharmacological treatments on the risk of major adverse cardiovascular events in patients with chronic kidney disease, with or without diabetes

Study	Drug	Statistic	All-cause mortality	MACE	CVD mortality	Myocardial infarction	Stroke	Hospitalization for HF
Qin et al., 2016[5]	RAASi	HR (95%CI)	0.83 (0.78-0.87)					
Balamuthusamy et al., 2008[6]	RAASi	RR (95%CI)	1.03 (0.92-1.04)	0.91 (0.85-0.98)	1.12 (0.95-1.32)	0.82 (0.67-1.00)	1.02 (0.81-1.28)	0.78 (0.66-0.92)
Xie et al., 2016[7]	ACEi	OR (95%CI)	0.87 (0.74-1.01)	0.82 (0.71-0.92)	0.88 (0.72-1.09)			
Zhang et al., 2020[8]	ACEi	OR (95%CI)	0.77 (0.66-0.91)	0.73 (0.64-0.84)	0.73 (0.63-0.86)			
Ninomiya et al., 2013[9]	ACEi	HR (95%CI)		0.81 (0.73-0.90)				
Xie et al., 2016[7]	ARBs	OR (95%CI)	0.99 (0.78-1.21)	0.76 (0.62-0.89)	1.12 (0.80-1.58)			
Zhang et al., 2020[8]	ARBs	OR (95%CI)	1.01 (0.82-1.25)	0.83 (0.70-0.98)	1.16 (0.88-1.53)			
Major et al., 2015[10]	Statins	RR (95%CI)	0.66 (0.49-0.88)	0.59 (0.48-0.72)			0.56 (0.28-1.13)	
Su et al., 2016[11]	Statins	OR (95%CI)		0.69 (0.61-0.79)				
Upadhyay et al., 2012[12]	Statins	RR (95%CI)	0.88 (0.79-0.98)	0.75 (0.68-0.84)				
Strippoli et al., 2008[13]	Statins	RR (95%CI)	0.81 (0.74-0.89)		0.80 (0.70-0.90)			
Palmer et al., 2014[14]	Statins	RR (95%CI)	0.81 (0.74-0.88)	0.76 (0.73-0.80)	0.78 (0.68-0.89)	0.55 (0.42-0.72)	0.61 (0.38-0.98)	
Hou et al., 2013[15]	Statins	RR (95%CI)	0.92 (0.85-0.99)	0.77 (0.70-0.85)	0.91 (0.84-0.99)		0.79 (0.56-1.12)	
Zhang et al., 2014[16]	Statins	RR (95%CI)	0.79 (0.72-0.86)	0.76 (0.72-0.80)	0.83 (0.73-0.93)	0.66 (0.52-0.83)	0.70 (0.57-0.85)	
Barylski et al., 2013[17]	Statins	RR (95%CI)	0.66 (0.55-0.79)	0.55 (0.40-0.75)	0.69 (0.55-0.78)		0.66 (0.50-0.88)	
Yamada et al., 2021[18]	SGLT-2i	RR (95%CI)		0.87 (0.76-1.00)				
Salah et al., 2021[19]	SGLT-2i	HR (95%CI)	0.82 (0.67-1.00)		0.84 (0.68-1.03)	0.72 (0.54-0.97)	0.67 (0.33-1.36)	0.61 (0.48-0.77)
Lin et al., 2021[20]	SGLT-2i	RR (95%CI)		0.84 (0.82-0.97				
Kamdar et al., 2021[21]	SGLT-2i	HR (95%CI)	0.89 (0.81-0.98)		0.90 (0.79-1.02)	0.82 (0.64-1.05)		0.69 (0.63-0.75)
D'Andrea et al., 2020[22]	SGLT-2i	HR (95%CI)		0.82 (0.69-0.97)				
Staplin et al., 2021[23]	SGLT-2i	RR (95%CI)	0.94 (0.75-1.12)	0.84 (0.76-0.93)	0.84 (0.73-0.97)			
Malik et al., 2020[24]	SGLT-2i	HR (95%CI)		0.80 (0.69-0.92)	0.88 (0.71-1.09)	0.78 (0.62-0.97)	0.83 (0.62-1.11)	0.61 (0.47-0.77)
Cao et al., 2021[25]	SGLT-2i	HR (95%CI)	0.83 (0.55-1.27)	0.75 (0.60-0.93)	0.89 (0.60-1.30)			0.74 (0.55-0.99)
Kaze et al., 2022[26]	SGLT-2i	HR (95%CI)	0.86 (0.77-0.96)	0.83 (0.75-0.93)	0.84 (0.74-0.96)	0.78 (0.67-0.92)	0.76 (0.59-0.97)	0.62 (0.55-0.71)
Yang et al., 2022[27]	SGLT-2i	OR (95%CI)	0.79 (0.66-0.93)		0.80 (0.65-0.98)			0.57 (0.45-0.72)

ACEi denotes angiotensin converting enzyme inhibitors; ARBs, angiotensin receptor blockers; CI, confidence interval; CVD, cardiovascular disease; HF, heart failure; HR, hazard ratio; MACE, major adverse cardiovascular events; OR, odds ratio; RAASi, renin-angiotensin aldosterone system inhibitors; RR, risk ratio; SGLT-2i, sodium-glucose cotransporter-2 inhibitors.

Sundström J, *et al. BMJ Open* 2024; 14:e074064. doi: 10.1136/bmjopen-2023-074064

Table S2. Inclusion criteria for the CELOSIA database from which the study sample was extracted

**Inclusion criteria (any of)**	**Description**
ICD10: I50	Heart failure
ICD10: I11.0	Hypertensive heart disease with (congestive) heart failure
ICD10: I13.0	Hypertensive heart and renal disease with (congestive) heart failure
ICD10: I13.2	Hypertensive heart and renal disease with both (congestive) heart failure and renal failure
ICD10: I25.5	Ischaemic cardiomyopathy
ICD10: I42.0	Dilated cardiomyopathy
ICD10: I42.6	Alcoholic cardiomyopathy
ICD10: I42.9	Cardiomyopathy, unspecified
ICD10: I43.1	Cardiomyopathy in metabolic diseases
ICD10: Z99.4	Dependence on artificial heart
Procedure: FQA	Transplantation of heart
Procedure: FQB	Transplantation of heart and lung
Procedure: FPE26	Implantation of transvenous cardiac pacemaker with biventricular electrodes
ATC: C01CX08	Levosimendan
BNP >100ng/L	Laboratory value
NT-proBNP >300ng/L	Laboratory value
ICD-10: E10.2	Type I diabetes mellitus with renal complications
ICD-10: E11.2	Type II diabetes mellitus with renal complications
ICD-10: E12.2	Malnutrition-related diabetes mellitus with renal complications
ICD-10: E13.2	Other specified diabetes mellitus with renal complications
ICD-10: E14:2	Unspecified diabetes mellitus with renal complications
ICD-10: N08.3	Glomerular disorders in diabetes mellitus
ICD-10: N17	Acute renal failure
ICD-10: N18	Chronic kidney failure
ICD-10: N19	Unspecified kidney failure
ICD-10: I12.0	Glomerular disorders in diabetes mellitus
ICD-10: I12.9	Hypertensive renal disease without renal failure
ICD-10: I13.1	Hypertensive heart and renal disease with renal failure
ICD-10: I13.9	Hypertensive heart and renal disease, unspecified
ICD-10: Z49.1	Extracorporeal dialysis
ICD-10: Z49.2	Other dialysis
ICD-10: Z99.2	Dependence on renal dialysis
Procedure: JAK10	Laparotomy and insertion of peritoneal dialysis catheter
Procedure: TJA33	Percutaneous introduction of peritoneal dialysis catheter
Procedure: TJA35	Removal of peritoneal dialysis catheter
Procedure: DJ008	Laparoscopic dialysis catheter insertion
Procedure: DR013	Initiation of continuos ambulatory peritoneal dialysis (CAPD)
Procedure: DR014	Hemodiafiltration (HDF)
Procedure: DR015	Hemodialysis, acute
Procedure: DR016	Hemodialysis, chronic
Procedure: DR017	Hemofiltration

Sundström J, *et al. BMJ Open* 2024; 14:e074064. doi: 10.1136/bmjopen-2023-074064

Procedure: DR023	Peritonealdialysis, acute
Procedure: DR024	Peritonealdialysis, chronic
Procedure: DR055	Citrate dialysis
Procedure: DR056	Heparin free dialysis
Procedure: DR060	Home hemodialysis control
Procedure: DR061	Home hemodialysis start
Procedure: KAS10	Allogenic transplantation of kidney from cadaver donor
Procedure: KAS20	Allogenic transplantation of kidney from living donor
Procedure: QF006	Peritoneal dialysis
eGFR <60 mL/min/1.73m^2	Laboratory value
U-albumin/creatinine ratio > 3 mg/mmol	Laboratory value
ICD-10: E10	Type I diabetes mellitus
ICD-10: E11	Type II diabetes mellitus
ICD-10: E12	Malnutrition-related diabetes mellitus
ICD-10: E13	Other specified diabetes mellitus
ICD-10: E14	Unspecified diabetes mellitus
ATC: A10	Drugs used in diabetes
HbA1c > 48 mmol/L	Laboratory value

Sundström J, *et al. BMJ Open* 2024; 14:e074064. doi: 10.1136/bmjopen-2023-074064

Table S3. Stages of chronic kidney disease diagnosed using measurements of eGFR and UACR according to the KDIGO clinical practice guidelines

**eGFR-defined stages**		
**Stage**	**eGFR thresholds (mL.min.1.73m²)**	**Abnormal**
1	≥ 90	No
2	60-89	No
3a	45-59	Yes
3b	30-44	Yes
4	15-29	Yes
5	GFR < 15	Yes
**Albuminuria-defined stages**		
**Stage**	**UACR thresholds (g/mol)**	**Abnormal**
A1	UACR < 3	No
A2	UACR 3-30	Yes
A3	UACR > 30	Yes

eGFR denotes estimated glomerular filtration rate; UACR, urinary albumin-creatinine ratio.

Sundström J, *et al. BMJ Open* 2024; 14:e074064. doi: 10.1136/bmjopen-2023-074064

Table S4. International Classification of Diseases (ICD)-10 codes used to search for diagnoses of CKD, prevalent comorbidities, and outcomes of interest

Condition	ICD-10 codes
**CKD diagnosis and exclusions**	
CKD (broad)	N17-N19, I12.0, I12.9, I13.1-I13.2, E10.2, E11.2, E12.2, E13.2, E14.2, N00-N08, N10-N16, Z49, Z99.2
CKD (narrow)	N17-N19, I12.0, I12.9, I13.1-I13.2, E10.2, E11.2, E12.2, E13.2, E14.2, N083, Z49.1-Z49.2, Z99.2
Kidney donation	Z52.4
**Comorbidities**	
Heart failure	I50, I11.0, I13.0, I13.2
Coronary heart disease	I20-I25
Acute myocardial infarction	I21
Unstable angina	I20.0
Stroke	I60-I66, G45
Atrial fibrillation	I48
Peripheral artery disease	I70-I72, I73.1, I73.9, I74, I77.3, I77.6, I77.8, I79
Diabetes	E10-E14
Cancer	C
Head and neck cancer	C00-C14
Digestive organs cancer	C15-C26
Lung and thorax cancer	C30-C39
Skin cancer	C43-C44
Breast cancer	C50
Female genital cancer	C51-C58
Male genital cancer	C61
Urinary cancer	C64-C68
Hematologic cancer	C81-C96
**Outcomes**	
CVD	I
MACE	I50, I11.0, I13.0, I13.2, I42, I43, I25.5, I21, I60-I62, I63.0-I63.5, I63.8-I63.9, I64

CKD denotes chronic kidney disease; CVD, cardiovascular disease; MACE, major cardiovascular events. ICD-10 codes for “CKD (broad)” were used to search for diagnoses of CKD prior to the index date, which would exclude them from the analyses. ICD-10 codes for “CKD (narrow) were used to search for diagnoses of CKD during follow-up.

Sundström J, *et al. BMJ Open* 2024; 14:e074064. doi: 10.1136/bmjopen-2023-074064

Table S5. Anatomical Therapeutic Chemical (ATC) classification system codes used to search for treatments of interest in this study's population

Treatment	ATC codes
RAASi	C09
Statin	C10AA, C10BA01-C10BA09, C10BA11-C10BA12, C10BX
SGLT-2i	A10BK01, A10BK02, A10BK03, A10BD15, A10BD16, A10BD20

RAASi denotes renin angiotensin aldosterone system inhibitors; SGLT-2i, sodium-glucose cotransporter-2 inhibitors.

Sundström J, *et al. BMJ Open* 2024; 14:e074064. doi: 10.1136/bmjopen-2023-074064

Table S6. Baseline characteristics of the total study cohort stratified by age group

	18-45 years	45-65 years	65-80 years	80+ years
**N**	1,441	9,955	47,319	40,667
**Female, N (%)**	706 (49.0%)	3,830 (38.5%)	22,143 (46.8%)	24,519 (60.3%)
**Age, median (IQR)**	37.8 (30.9-41.9)	59.6 (55.0-62.7)	74.2 (70.8-77.1)	85.5 (82.6-89.1)
**Socioeconomic status, N (%)**				
Missing	776 (53.9%)	5,898 (59.2%)	30,480 (64.4%)	27,947 (68.7%)
High	90 (13.5%)	673 (16.6%)	3,743 (22.2%)	2,848 (22.4%)
Middle	216 (32.5%)	1,510 (37.2%)	7,345 (43.6%)	5,083 (40.0%)
Low	359 (54.0%)	1,874 (46.2%)	5,751 (34.2%)	4,789 (37.6%)
**Comorbidities, N (%)**				
Heart failure	45 (3.1%)	1,173 (11.8%)	7,982 (16.9%)	11,090 (27.3%)
CHD	20 (1.4%)	1,574 (15.8%)	12,268 (25.9%)	12,749 (31.3%)
AMI	9 (0.6%)	772 (7.8%)	5,463 (11.5%)	5,041 (12.4%)
Unstable angina	<=5	357 (3.6%)	2,860 (6.0%)	2,279 (5.6%)
Stroke	31 (2.2%)	828 (8.3%)	7,181 (15.2%)	8,677 (21.3%)
Atrial fibrillation	24 (1.7%)	1,038 (10.4%)	11,049 (23.4%)	13,263 (32.6%)
PAD	31 (2.2%)	510 (5.1%)	4,637 (9.8%)	3,975 (9.8%)
Diabetes	775 (53.8%)	5,031 (50.5%)	16,545 (35.0%)	9,394 (23.1%)
Cancer	58 (4.0%)	1,516 (15.2%)	14,615 (30.9%)	14,628 (36.0%)
Head and neck	6 (0.4%)	53 (0.5%)	396 (0.8%)	347 (0.9%)
Digestive	<=5	197 (2.0%)	1,739 (3.7%)	1,786 (4.4%)
Lung and thorax	<=5	112 (1.1%)	880 (1.9%)	455 (1.1%)
Skin	<=5	313 (3.1%)	5,123 (10.8%)	7,991 (19.6%)
Breast	<=5	185 (1.9%)	2,147 (4.5%)	1,836 (4.5%)
Female genital	<=5	119 (1.2%)	884 (1.9%)	778 (1.9%)
Male genital	<=5	160 (1.6%)	3,253 (6.9%)	2,397 (5.9%)
Urinary	10 (0.7%)	283 (2.8%)	1,653 (3.5%)	1,066 (2.6%)
Hematologic	13 (0.9%)	216 (2.2%)	1,215 (2.6%)	787 (1.9%)
**Measurements, median (IQR)**				
Systolic BP (mmHg)	130.0 (120.0-140.0)	137.0 (125.0-150.0)	140.0 (126.0-150.0)	140.0 (129.0-154.5)
BMI (kg/m²)	28.8 (24.3-34.4)	29.3 (25.7-33.4)	27.1 (24.2-30.7)	24.8 (22.2-28.0)
**Laboratory, median (IQR)**				
P-creatinine (µmol/L)	71.0 (57.0-94.0)	94.0 (72.0-115.0)	97.0 (84.0-109.0)	90.0 (78.0-105.0)
eGFR (mL.min.1.73m²)	94.2 (74.0-107.0)	59.2 (53.6-83.8)	54.6 (49.3-58.2)	50.5 (42.8-55.8)
UACR (g/mol)	9.0 (4.7-24.0)	6.4 (3.6-15.2)	3.3 (0.7-8.1)	2.5 (0.7-6.9)
Sodium (mmol/L)	139.0 (138.0-141.0)	140.0 (138.0-141.0)	140.0 (138.0-142.0)	140.0 (138.0-142.0)
Potassium (mmol/L)	4.0 (3.8-4.3)	4.2 (3.9-4.4)	4.2 (4.0-4.4)	4.2 (3.9-4.4)
HbA1c (mmol/mol)	54.0 (40.0-73.0)	50.0 (40.0-65.0)	44.0 (39.0-54.0)	42.0 (38.0-51.0)
Hemoglobin (g/L)	139.5 (126.0-152.0)	140.0 (129.0-152.0)	136.0 (126.0-146.0)	131.0 (120.0-141.0)
**Medications, N (%)**				
Statins	234 (16.2%)	4,012 (40.3%)	22,233 (47.0%)	14,253 (35.0%)
RAASi	452 (31.4%)	5,952 (59.8%)	29,472 (62.3%)	20,894 (51.4%)
SGLT2i	26 (1.8%)	335 (3.4%)	547 (1.2%)	78 (0.2%)

Sundström J, *et al. BMJ Open* 2024; 14:e074064. doi: 10.1136/bmjopen-2023-074064

Data for baseline demographics and prevalent comorbidities were collected for each patient on the date this study identified their CKD (index date) using eGFR and UACR measurements according to the KDIGO clinical practice guidelines. The most recent clinical measurements and laboratory values within the one year prior to the index date are reported. For patients residing in Region Stockholm, the most recent socioeconomic data within the 10 years prior to the index date was collected from the Mosaic system, which applies the principles of geodemography to consumer household and individual data to categorize patients into one of the following three levels: 1) Highest income/education; 2) Medium income/education; and 3) Lowest income/education. AMI denotes acute myocardial infarction; BMI, body mass index; BP, blood pressure; CHD, coronary heart disease; eGFR, estimated glomerular filtration rate; HbA1c, glycated hemoglobin; IQR, interquartile range; PAD, peripheral artery disease; RAASi, renin-angiotensin aldosterone system inhibitors; SGLT2i, sodium-glucose cotransporter-2 inhibitors; UACR, urinary albumin-creatinine ratio.

Sundström J, *et al. BMJ Open* 2024; 14:e074064. doi: 10.1136/bmjopen-2023-074064

Table S7. Proportions of patients diagnosed with chronic kidney disease in healthcare within five years of the development of the disease

**Stage of CKD**	**Percentage with a diagnosis in healthcare within 5 years**
S3a	29%
S3b	59%
S4+S5	82%
A2	34%
A3	62%

Each stage of chronic kidney disease (CKD) is defined using measurements of estimated glomerular filtration rate (stages S3a, S3b, and S4+S5) and urinary albumin-creatinine ratio (stages A2 and A3) according to the KDIGO clinical practice guidelines.

Sundström J, *et al. BMJ Open* 2024; 14:e074064. doi: 10.1136/bmjopen-2023-074064

Figure S1. Participant flow

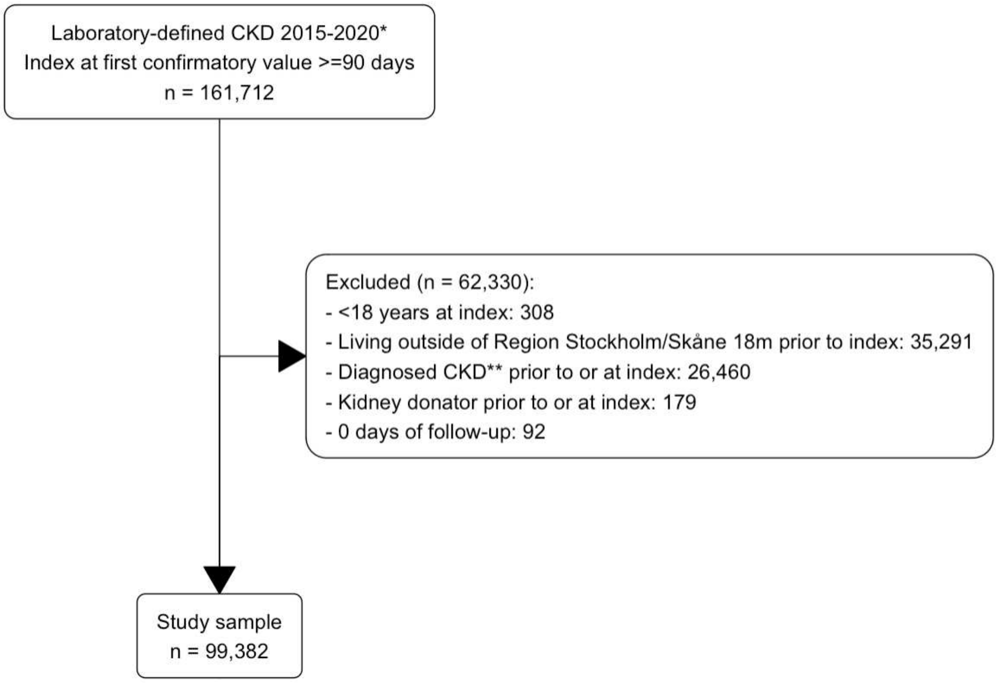

*Laboratory-defined CKD = KDIGO confirmed definition; **Diagnosed CKD = Any diagnosis position, any care level.

Sundström J, *et al. BMJ Open* 2024; 14:e074064. doi: 10.1136/bmjopen-2023-074064

Figure S2. Multi-state models presenting the proportions of patients diagnosed or not diagnosed with chronic kidney disease (CKD) in healthcare who received or didn't receive guideline-directed pharmacological therapy, including the terminal, absorbing outcome, all-cause death

A) Statins

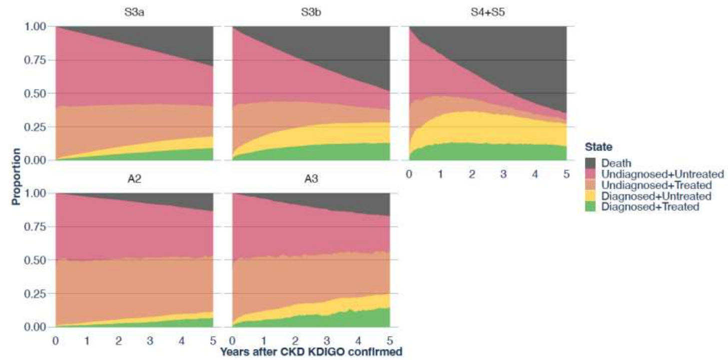

B) RAASi

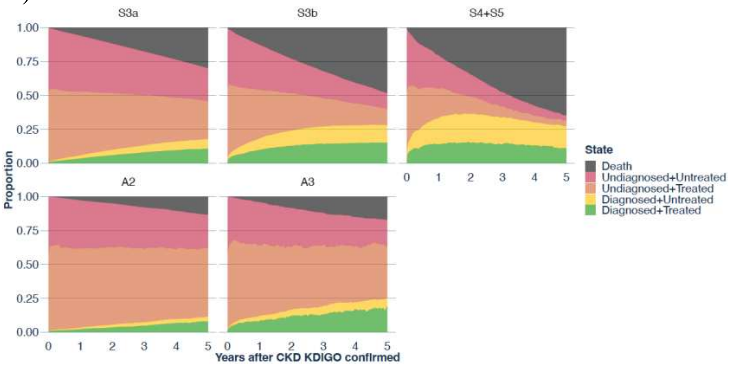

C) SGLT2i

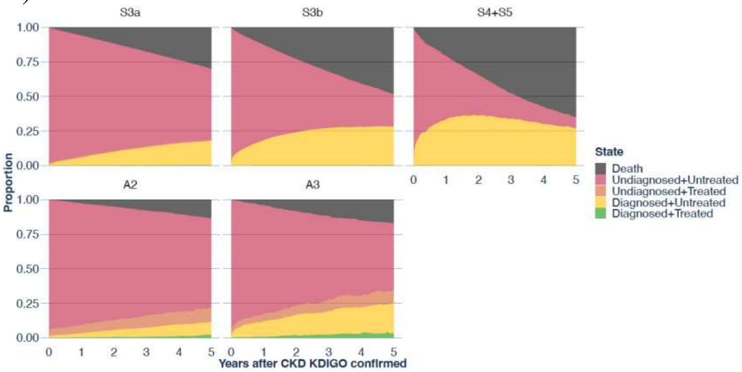

Use of A) Statins, B) renin-angiotensin aldosterone system inhibitors (RAASi), and C) sodium-glucose cotransporter-2 inhibitors (SGLT2i) were assessed. Patients are stratified by the KDIGO-defined stage of their CKD, identified using estimated glomerular filtration rate (Stages S3a, S3b, and S4+S5) and urinary albumin-creatinine ratio (Stages A2 and A3).

Sundström J, *et al. BMJ Open* 2024; 14:e074064. doi: 10.1136/bmjopen-2023-074064

Figure S3. Multi-state models presenting the proportions of patients diagnosed or not diagnosed with chronic kidney disease (CKD) in healthcare who received or didn't receive guideline-directed pharmacological therapy, including the terminal, absorbing outcomes, major adverse cardiovascular events (MACE) and non-cardiovascular disease (CVD) death

A) Statins

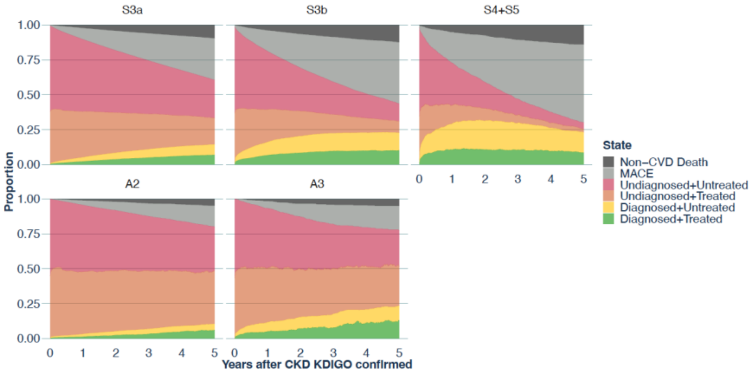

B) RAASi

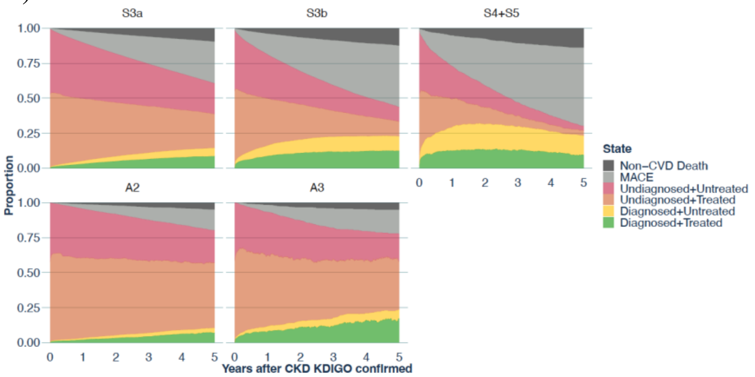

C) SGLT2i

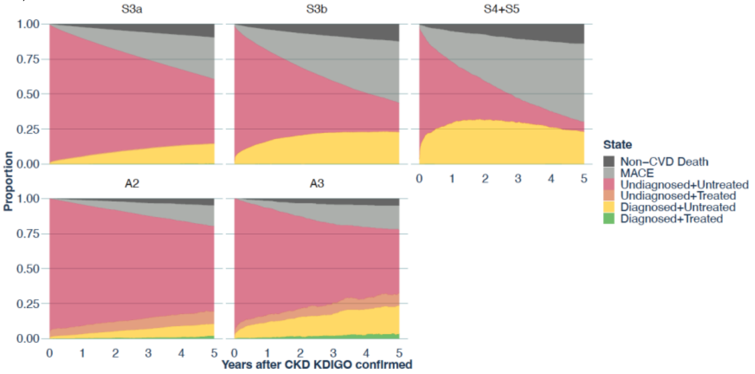

Use of A) Statins, B) renin-angiotensin aldosterone system inhibitors (RAASi), and C) sodium-glucose cotransporter-2 inhibitors (SGLT2i) were assessed. Patients are stratified by the KDIGO-defined stage of their CKD, identified using estimated glomerular filtration rate (Stages S3a, S3b, and S4+S5) and urinary albumin-creatinine ratio (Stages A2 and A3).

Sundström J, *et al. BMJ Open* 2024; 14:e074064. doi: 10.1136/bmjopen-2023-074064

Figure S4. Multi-state models presenting the proportions of patients diagnosed or not diagnosed with chronic kidney disease (CKD) in healthcare who received or didn't receive guideline-directed pharmacological therapy, including the terminal, absorbing outcome, all-cause death

A) Statins

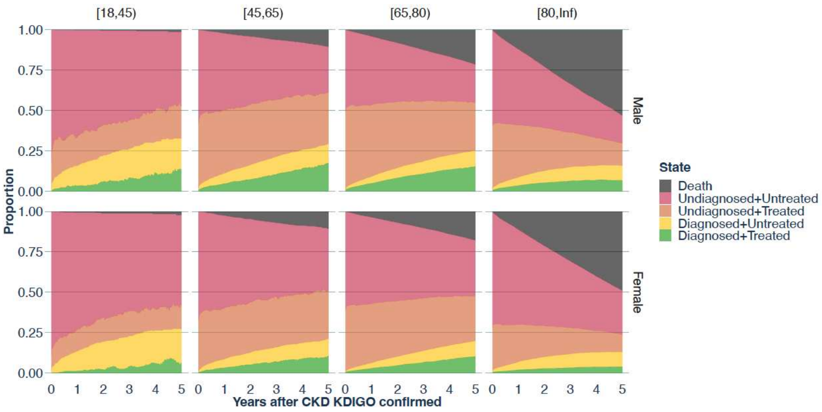

B) RAASi

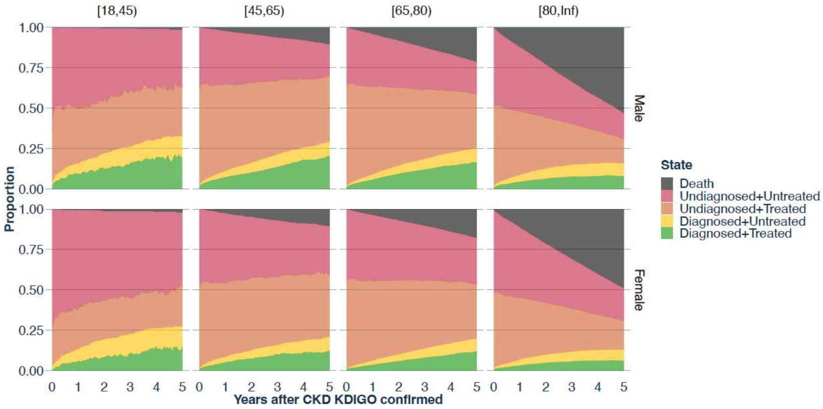

C) SGLT2i

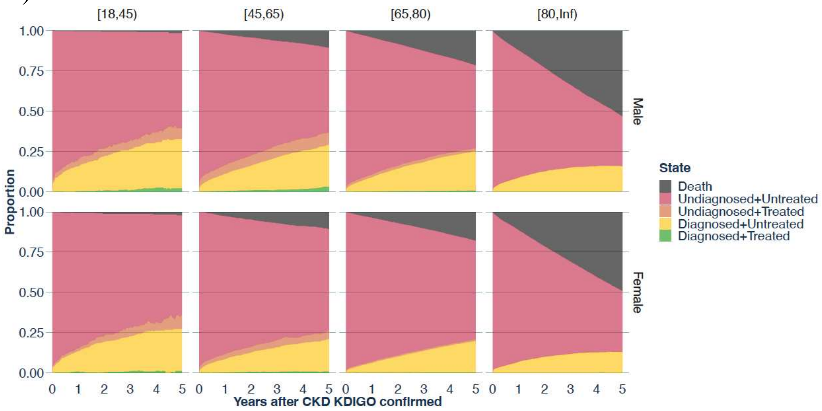

Use of A) Statins, B) renin-angiotensin aldosterone system inhibitors (RAASi), and C) sodium-glucose cotransporter-2 inhibitors (SGLT2i) were assessed. Patients are stratified by gender and age group.

Figure S5. Multi-state models presenting the proportions of patients diagnosed or not diagnosed with chronic kidney disease (CKD) in healthcare who received or didn't receive guideline-directed pharmacological therapy, including the terminal, absorbing outcomes, major adverse cardiovascular events (MACE) and non-cardiovascular disease (CVD) death

A) Statins

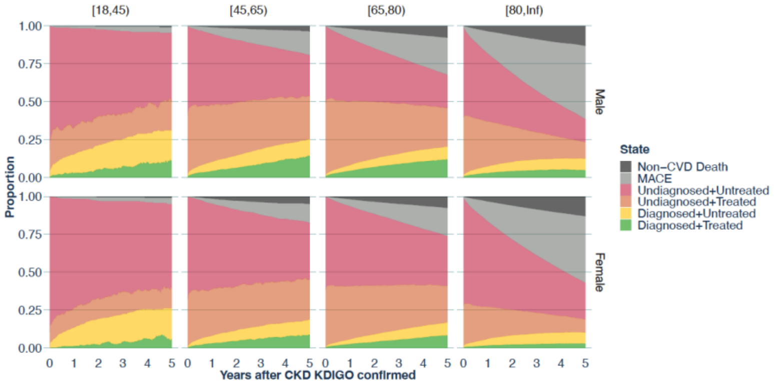

B) RAASi

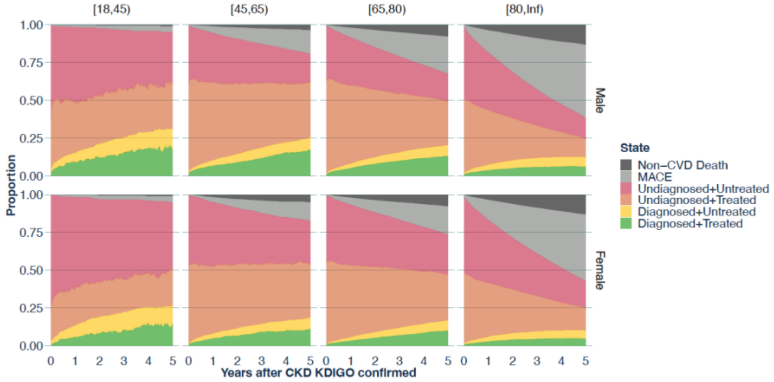

C) SLGT2i

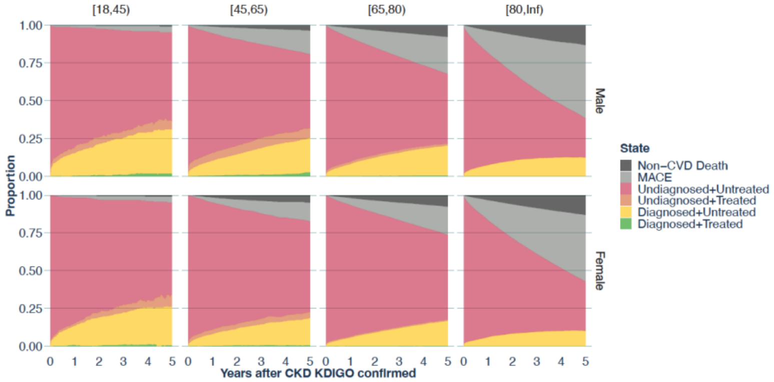

Use of A) Statins, B) renin-angiotensin aldosterone system inhibitors (RAASi), and C) sodium-glucose cotransporter-2 inhibitors (SGLT2i) were assessed. Patients are stratified by gender and age group.

Sundström J, *et al. BMJ Open* 2024; 14:e074064. doi: 10.1136/bmjopen-2023-074064

References

1. WHO Collaborating Centre for Drug Statistics Methodology. ATC/DDD Index 2017. https://www.whocc.no/.
2. WHO. Classifications. http://www.who.int/classifications/icd/en/.
3. (NOMESCO), N. M.-S. C. NOMESCO Classification of Surgical Procedures. https://norden.diva-portal.org/smash/get/diva2:970547/FULLTEXT01.pdf (2010).
4. Ludvigsson, J. F., Otterblad-Olausson, P., Pettersson, B. U. & Ekbom, A. The Swedish personal identity number: Possibilities and pitfalls in healthcare and medical research. *European Journal of Epidemiology* **24**, 659–667 (2009).
5. Qin, Y. *et al.* The effect of angiotensin-converting enzyme inhibitor/angiotensin receptor blocker use on mortality in patients with chronic kidney disease: a meta-analysis of observational studies. *Pharmacoepidemiol Drug Saf* **25**, 503–511 (2016).
6. Balamuthusamy, S. *et al.* Renin angiotensin system blockade and cardiovascular outcomes in patients with chronic kidney disease and proteinuria: a meta-analysis. *Am Heart J* **155**, 791–805 (2008).
7. Xie, X. *et al.* Renin-Angiotensin System Inhibitors and Kidney and Cardiovascular Outcomes in Patients With CKD: A Bayesian Network Meta-analysis of Randomized Clinical Trials. *American Journal of Kidney Diseases* **67**, 728–741 (2016).
8. Zhang, Y. *et al.* ACE Inhibitor Benefit to Kidney and Cardiovascular Outcomes for Patients with Non-Dialysis Chronic Kidney Disease Stages 3–5: A Network Meta-Analysis of Randomised Clinical Trials. *Drugs* **80**, 797–811 (2020).
9. Collaboration, B. P. L. T. T. Blood pressure lowering and major cardiovascular events in people with and without chronic kidney disease: meta-analysis of randomised controlled trials. *BMJ* **347**, f5680 (2013).
10. Major, R. W., Cheung, C. K., Gray, L. J. & Brunskill, N. J. Statins and Cardiovascular Primary Prevention in CKD: A Meta-Analysis. *Clin J Am Soc Nephrol* **10**, 732–739 (2015).
11. Su, X. *et al.* Effect of Statins on Kidney Disease Outcomes: A Systematic Review and Meta-analysis. *American Journal of Kidney Diseases* **67**, 881–892 (2016).
12. Upadhyay, A. *et al.* Lipid-lowering therapy in persons with chronic kidney disease: a systematic review and meta-analysis. *Ann Intern Med* **157**, 251–262 (2012).
13. Strippoli, G. F. M. *et al.* Effects of statins in patients with chronic kidney disease: meta-analysis and meta-regression of randomised controlled trials. *BMJ* **336**, 645–651 (2008).
14. Palmer, S. C. *et al.* Benefits and harms of statin therapy for persons with chronic kidney disease: a systematic review and meta-analysis. *Ann Intern Med* **157**, 263–275 (2012).
15. Hou, W. *et al.* Effect of statin therapy on cardiovascular and renal outcomes in patients with chronic kidney disease: a systematic review and meta-analysis. *Eur Heart J* **34**, 1807–1817 (2013).
16. Zhang, X. *et al.* Effect of statins on cardiovascular events in patients with mild to moderate chronic kidney disease: a systematic review and meta-analysis of randomized clinical trials. *BMC Cardiovasc Disord* **14**, 19 (2014).
17. Barylski, M. *et al.* Statins decrease all-cause mortality only in CKD patients not requiring dialysis therapy—A meta-analysis of 11 randomized controlled trials involving 21,295 participants. *Pharmacological Research* **72**, 35–44 (2013).
18. Yamada, T. *et al.* Cardiovascular and renal outcomes with SGLT-2 inhibitors versus GLP-1 receptor agonists in patients with type 2 diabetes mellitus and chronic kidney disease: a systematic review and network meta-analysis. *Cardiovascular Diabetology* **20**, 14 (2021).

19. Salah, H. M. *et al.* Effect of sodium-glucose cotransporter 2 inhibitors on cardiovascular and kidney outcomes-Systematic review and meta-analysis of randomized placebo-controlled trials. *Am Heart J* **232**, 10–22 (2021).
20. Lin, D. S.-H., Lee, J.-K., Hung, C.-S. & Chen, W.-J. The efficacy and safety of novel classes of glucose-lowering drugs for cardiovascular outcomes: a network meta-analysis of randomised clinical trials. *Diabetologia* **64**, 2676–2686 (2021).
21. Kamdar, A., Sykes, R., Morrow, A., Mangion, K. & Berry, C. Cardiovascular outcomes of glucose lowering therapy in chronic kidney disease patients: a systematic review with meta-analysis. *Rev Cardiovasc Med* **22**, 1479–1490 (2021).
22. D'Andrea, E. *et al.* Heterogeneity of antidiabetic treatment effect on the risk of major adverse cardiovascular events in type 2 diabetes: a systematic review and meta-analysis. *Cardiovasc Diabetol* **19**, 154 (2020).
23. Staplin, N. *et al.* Net effects of sodium-glucose co-transporter-2 inhibition in different patient groups: a meta-analysis of large placebo-controlled randomized trials. *EClinicalMedicine* **41**, 101163 (2021).
24. Malik, A. H. *et al.* Cardiovascular Outcomes With the Use of Sodium-Glucose Cotransporter-2 Inhibitors in Patients With Type 2 Diabetes and Chronic Kidney Disease: An Updated Meta-Analysis of Randomized Controlled Trials. *Cardiol Rev* **28**, 116–124 (2020).
25. Cao, H. *et al.* Sodium-glucose cotransporter 2 inhibitors benefit to kidney and cardiovascular outcomes for patients with type 2 diabetes mellitus and chronic kidney disease 3b-4: A systematic review and meta-analysis of randomized clinical trials. *Diabetes Research and Clinical Practice* **180**, (2021).
26. Kaze, A. D., Zhuo, M., Kim, S. C., Patorno, E. & Paik, J. M. Association of SGLT2 inhibitors with cardiovascular, kidney, and safety outcomes among patients with diabetic kidney disease: a meta-analysis. *Cardiovascular Diabetology* **21**, 47 (2022).
27. Yang, S., Zhao, L., Mi, Y. & He, W. Effects of sodium-glucose cotransporter-2 inhibitors and aldosterone antagonists, in addition to renin-angiotensin system antagonists, on major adverse kidney outcomes in patients with type 2 diabetes and chronic kidney disease: A systematic review and network meta-analysis. *Diabetes, Obesity and Metabolism* **n/a**,.

Sundström J, *et al. BMJ Open* 2024; 14:e074064. doi: 10.1136/bmjopen-2023-074064
