# Supplementary material for: Are there lost opportunities in chronic kidney disease? A region-wide cohort study
Source: BMJ Open. 2024 Apr 19;14(4):e074064. doi: 10.1136/bmjopen-2023-074064 (PMC11033666; doi:10.1136/bmjopen-2023-074064)
Supplement: Author's manuscript [file bmjopen-2023-074064.draft_revisions.pdf]

BMJ Open is committed to open peer review. As part of this commitment we make the peer review history of every article we publish publicly available.

When an article is published we post the peer reviewers' comments and the authors' responses online. We also post the versions of the paper that were used during peer review. These are the versions that the peer review comments apply to.

The versions of the paper that follow are the versions that were submitted during the peer review process. They are not the versions of record or the final published versions. They should not be cited or distributed as the published version of this manuscript.

BMJ Open is an open access journal and the full, final, typeset and author-corrected version of record of the manuscript is available on our site with no access controls, subscription charges or pay-per-view fees (http://bmjopen.bmj.com).

If you have any questions on BMJ Open's open peer review process please email info.bmjopen@bmj.com

BMJ Open

Lost opportunities in chronic kidney disease

Journal:	*BMJ Open*
Manuscript ID	bmjopen-2023-074064
Article Type:	Original research
Date Submitted by the Author:	26-Mar-2023
Complete List of Authors:	Sundström, Johan; Uppsala University, Department of Medical Sciences; University of New South Wales, The George Institute for Global Health<br>Norhammar, Anna; Karolinska Institutet, Department of Medicine; Capio S:t Görans Hospital<br>Karayiannides, Stelios; Karolinska Institute, Department of Clinical Sciences, Danderyd Hospital ; Region Stockholm, Center for Diabetes, Academic Specialist Center<br>Bodegård, Johan; AstraZeneca PLC, Renal and Metabolism, Medical Department, BioPharmaceuticals<br>Gustafsson, Stefan; Sence Research AB<br>Cars, Thomas; Sence Research AB<br>Eriksson Svensson, Maria; Uppsala University, Department of Medical Sciences Renal Medicine<br>Ärnlöv, Johan; Karolinska Institutet, Division of Family Medicine, Department of Neurobiology, Care Sciences and Society; Dalarna University, School of Health and Social Studies
Keywords:	EPIDEMIOLOGY, Chronic renal failure < NEPHROLOGY, Adult nephrology < NEPHROLOGY

SCHOLARONE™
Manuscripts

I, the Submitting Author has the right to grant and does grant on behalf of all authors of the Work (as defined in the below author licence), an exclusive licence and/or a non-exclusive licence for contributions from authors who are: i) UK Crown employees; ii) where BMJ has agreed a CC-BY licence shall apply, and/or iii) in accordance with the terms applicable for US Federal Government officers or employees acting as part of their official duties; on a worldwide, perpetual, irrevocable, royalty-free basis to BMJ Publishing Group Ltd ("BMJ") its licensees and where the relevant Journal is co-owned by BMJ to the co-owners of the Journal, to publish the Work in this journal and any other BMJ products and to exploit all rights, as set out in our licence.

The Submitting Author accepts and understands that any supply made under these terms is made by BMJ to the Submitting Author unless you are acting as an employee on behalf of your employer or a postgraduate student of an affiliated institution which is paying any applicable article publishing charge ("APC") for Open Access articles. Where the Submitting Author wishes to make the Work available on an Open Access basis (and intends to pay the relevant APC), the terms of reuse of such Open Access shall be governed by a Creative Commons licence – details of these licences and which Creative Commons licence will apply to this Work are set out in our licence referred to above.

Other than as permitted in any relevant BMJ Author's Self Archiving Policies, I confirm this Work has not been accepted for publication elsewhere, is not being considered for publication elsewhere and does not duplicate material already published. I confirm all authors consent to publication of this Work and authorise the granting of this licence.

For peer review only

Lost opportunities in chronic kidney disease

Johan Sundström (1,2), Anna Norhammar (3,4), Stelios Karayiannides (5,6), Johan Bodegard (7), Stefan Gustafsson (8), Thomas Cars (8) Maria K Svensson (9), Johan Ärnlöv (10,11)

1) Department of Medical Sciences, Uppsala University, Uppsala, Sweden
2) The George Institute for Global Health, University of New South Wales, Sydney, Australia
3) Cardiology Unit, Department of Medicine, Solna, Karolinska Institute, Stockholm, Sweden
4) Capio S:t Görans Hospital, Stockholm, Sweden
5) Department of Clinical Sciences, Karolinska Institutet, Danderyd Hospital, Stockholm, Sweden
6) Center for Diabetes, Academic Specialist Center, Region Stockholm, Sweden
7) Cardiovascular, Renal and Metabolism, Medical Department, BioPharmaceuticals, AstraZeneca, Oslo, Norway
8) Sence Research AB, Uppsala, Sweden
9) Department of Medical Sciences Renal Medicine, Uppsala University, Uppsala, Sweden
10) Division of Family Medicine, Department of Neurobiology, Care Sciences and Society, Karolinska Institutet, Huddinge, Sweden
11) School of Health and Social Studies, Dalarna University, Falun, Sweden

Word count: 3529

Correspondence to:
Professor Johan Sundström, MD PhD
Department of Medical Sciences, Uppsala University
Entrance 40, 5th floor; Uppsala University Hospital; 75185 Uppsala, Sweden
johan.sundstrom@uu.se
+46704225220

For peer review only - http://bmjopen.bmj.com/site/about/guidelines.xhtml

ABSTRACT

Objectives

Identify the windows of opportunity for diagnosis of chronic kidney disease (CKD) and the prevention of its adverse outcomes, and to quantify the potential population gains of such prevention.

Design and setting

Observational, population-wide study of residents in the Stockholm and Skåne regions of Sweden between January 1st, 2015, and December 31st, 2020.

Participants

All patients who didn't yet have a diagnosis of CKD in healthcare, but had CKD according to laboratory measurements of CKD biomarkers available in electronic health records.

Outcome measures

We assessed the proportions of the patient population that received a subsequent diagnosis of CKD in healthcare, that used guideline-directed pharmacological therapy (statins, renin-angiotensin aldosterone system inhibitors [RAASi], and/or sodium-glucose cotransporter-2 inhibitors [SGLT2i]), and that experienced adverse outcomes (all-cause mortality or major adverse cardiovascular events [MACE]). The potential to prevent adverse outcomes in CKD was assessed using simulations of guideline-directed pharmacological therapy in untreated subsets of the study population.

Results

We identified 99,382 patients with undiagnosed CKD during the study period. Only 33% of those received a subsequent diagnosis of CKD in healthcare after five years. The proportion that used statins or RAASi was of similar size to the proportion that didn't, regardless of how advanced their CKD was. Use of SGLT2i was negligible. In simulations, 5,535 (25%) of the 21,737 deaths and 8,418 (38%) of the 22,224 MACE could have been avoided if every patient that didn't use an indicated medication for their laboratory-confirmed CKD was treated with guideline-directed pharmacological therapy for CKD.

Conclusions

While we noted critical under-diagnosis and under-treatment of CKD in this large contemporary population, we also identified a substantial realizable potential to improve CKD outcomes and reduce its burden by treating patients early with guideline-directed pharmacological therapy.

For peer review only - http://bmjopen.bmj.com/site/about/guidelines.xhtml

STRENGTHS AND LIMITATIONS OF THIS STUDY

- This study was strengthened by its access to health registries and electronic health records detailing most incidences of healthcare and medication use by residents in two of Sweden's largest regions, representing over a third of the national population.

- Given that only persons with suspected or known other disease have provided the samples necessary to make a laboratory-based assessment of CKD status, it is likely that this study underestimates the number of patients with undiagnosed CKD.

- This study couldn't assess if patients from a specific socioeconomic background or ethnicity were disproportionally affected by the deficiencies in CKD-related healthcare.

- Simulations of guideline-directed pharmacological therapy assumed that treatment was initiated at the time CKD was identified using laboratory measurements and that the adherence to treatment would be 100%, efficiency and success in treatment that is unlikely in routine clinical practice.

For peer review only

For peer review only - http://bmjopen.bmj.com/site/about/guidelines.xhtml

INTRODUCTION

Current estimates indicate that one in ten adults has CKD (between 700 million and one billion people globally).(1–3) As the prevalence of CKD increases with an aging population and growing rates of CKD risk factors (e.g., diabetes and hypertension),(4) so too does its contribution to total mortality and its economic burden;(2,5,6) highlighting the need to evaluate CKD diagnosis and treatment patterns.

Given that cardiovascular disease is a major cause of premature death among patients with CKD, management of cardiovascular disease risk factors (e.g., high cholesterol, hypertension, and chronic hyperglycemia) remains central to the treatment of CKD. Statins, renin-angiotensin aldosterone system inhibitors (RAASi), and sodium-glucose cotransporter-2 inhibitors (SGLT2i) are first-line pharmacological therapies in patients with CKD;(7–9) each demonstrated to reduce the risk of major adverse cardiovascular events (MACE) in this patient population (Table S1). Data from recent large cohort studies suggest that low proportions of patients with any degree of CKD are treated with these drugs.(1,10) While low use of SGLT2i may be explained by the recency of their inclusion in CKD treatment guidelines,(11,12) it is less clear as to why statins and RAASi have been underused, considering that they have been readily available in clinical practice for more than 20 years. Hence, detailed characterization of prevalence and consequences of sub-optimal CKD-related prevention and healthcare, and determination of the potential for improving outcomes in patients with CKD, are of great public health importance.

For that purpose, we identified a large cohort of patients with CKD using laboratory data in electronic health records, with the aim to detail the proportions of those patients that received a subsequent diagnosis of CKD in healthcare, that used guideline-directed pharmacological therapy for their CKD, and that developed adverse outcomes. We also aimed to identify windows of opportunity for preventing adverse outcomes in patients with CKD, by assessing the absolute numbers of adverse outcomes that would be prevented by the simulated application of several variants of guideline-directed pharmacological therapy.

METHODS

Data sources

Sweden has a comprehensive, nationwide public healthcare system to which each resident has access with a minor co-payment for healthcare visits, hospitalizations and medications.(13) Residents have a unique personal identification number (person-ID),(14) which is mandatory for all administrative purposes, including any contact with the healthcare system and filling of drug prescriptions. Thus, providing a basis for complete population-wide medical history.

The present study utilized data from the CELOSIA database, the details of which can be viewed in the Supplemental Methods. Briefly, it includes data from the Swedish Prescribed Drug Register, the Cause of Death Register, the National Patient Register, and from several data sources with regional coverage including electronic health records in the Stockholm and Skåne regions. A complete list of the Anatomical Therapeutic Chemical (ATC)

For peer review only - http://bmjopen.bmj.com/site/about/guidelines.xhtml

codes,(15) International Classification of Diseases (ICD)-10 codes,(16) procedure codes,(17) and laboratory values used in the CELOSIA database is available in Table S2.

Individual patient-level data from the national registers and regional data sources were linked using the person-ID. The linked pseudonymized database was managed separately by Sence Research AB, Uppsala, Sweden. The study was approved by the Swedish Ethical Review Authority (approval numbers 2020-03850 and 2020-06716).

Study population

Participant flow through the study is described in Figure S1. Laboratory measurements of creatinine-based estimated glomerular filtration rate (eGFR) and/or urine albumin-creatinine ratio (UACR), available in the electronic health records, were used to identify patients >18 years of age with CKD (irrespectively of whether it was diagnosed, indicated by the presence of an ICD code in healthcare databases) according to the Kidney Disease: Improving Global Outcomes (KDIGO) clinical practice guidelines.(8) Meaning, CKD was identified if a patient presented at least two abnormal eGFR measurements (<60 ml/min/1.73m^2) and/or two abnormal UACR measurements (≥3 g/mol), with the first and last measurements taken at least 90 days apart and where all measurements in between were abnormal.

The last abnormal measurement, which (would have) allowed the identification of CKD, needed to have been taken between January 1st, 2015, and December 31st, 2020. The first abnormal measurement, however, could have been taken prior to the start of that study period. The date at which CKD was identified was considered the index date. The revised Lund-Malmö equation was used to calculate eGFR from creatinine, age and sex;(18) and UACR was expressed in g/mol. The eGFR and UACR thresholds defining each stage of CKD according to KDIGO guidelines are detailed in Table S3.

At the time of and within the 18 months prior to the date that CKD was identified (index), patients needed to be residing in Region Stockholm or Region Skåne where electronic health records were available. Patients with a prior diagnosis of CKD in healthcare or who had donated a kidney were excluded (ICD-10 codes used to identify those patients are listed in Table S4; ICD-10 codes for a broad CKD definition were used). Patients with zero days of follow-up (i.e., those who died at the time CKD was identified) were also excluded (n=92).

Follow-up and characterization of exposures, outcomes, and comorbidities

Patients were followed for up to five years or until the censor date (December 31st, 2020), whichever occurred first. Adverse outcomes of interest during follow-up were all-cause death, non-cardiovascular death, and MACE, where MACE was defined as a primary diagnosis of MACE in inpatient care or a primary/non-primary diagnosis of death caused by cardiovascular disease.

To evaluate the lapse in time between the laboratory-based identification of CKD and a subsequent diagnosis of CKD in healthcare, ICD-10 codes indicating a CKD diagnosis in healthcare were also searched for. Prevalent comorbidities were defined as all diagnoses up until the laboratory-based identification of CKD. Diagnoses available from primary care, specialized outpatient care and inpatient care were used, including both primary and non-

For peer review only - http://bmjopen.bmj.com/site/about/guidelines.xhtml

primary diagnoses. The ICD-10 codes used to identify diagnoses of CKD in healthcare (codes for a narrow CKD definition were used), prevalent comorbidities, and the adverse outcomes of interest are listed in Table S4.

To characterize the use of guideline-directed pharmacological therapy within the total study population, filled prescriptions for statins, RAASi, and SGLT2i were searched for using the ATC codes listed in Table S5. For each medication, a patient was considered treated throughout the follow-up period if a new prescription was filled before the coverage period of the previous filled prescription expired. The coverage period for each filled prescription was calculated as the total number of pills dispensed divided by the prescribed daily dosage. This coverage period was extended by a 25% grace period, allowing for small deviations (e.g., potentially due to imperfect medication adherence) from the expected dispensation pattern.

Statistical analyses

All statistical analyses were performed using R version 3.6.0, and the R package Epi was used for all multi-state analyses.(19,20)

Proportions of patients with diagnoses, treatments, and adverse outcomes

To detail the proportion of patients with laboratory-identified CKD that received a subsequent diagnosis of CKD in healthcare and/or used a guideline-directed pharmacological therapy (statins, RAASi, and/or SGLT2i), as well as the proportion affected by adverse outcomes, multi-state models were constructed with four possible transient states:

1) Undiagnosed and untreated.
2) Undiagnosed and treated.
3) Diagnosed and untreated.
4) Diagnosed and treated.

The transient nature of the multi-state models meant that any change in a patient's state during follow-up was accounted for by the model and reflected in the results (e.g., any change in treatment status over time or at the first diagnosis of CKD in healthcare). Each model also included either all-cause death, non-cardiovascular disease death, or MACE as terminal, absorbing states.

In figures for the multi-state models and all tables where patients are stratified by the severity of their CKD, CKD was identified in each patient primarily using eGFR measurements. If eGFR data were not available, UACR measurements were used. To detail the lapse in time between the laboratory-based identification of CKD and a diagnosis of CKD in healthcare, a treatment, and/or an adverse event, the Aalen-Johansen estimator was calculated along the time scale, years since the laboratory-based identification of CKD. Kaplan-Meier curves were used to present the proportions of patients who received a diagnosis of CKD in healthcare after the laboratory-based identification of CKD.

Simulations of population effects of guideline-directed pharmacological therapy

To evaluate the potential to prevent adverse outcomes in patients with CKD, several variants of guideline-directed pharmacological therapy were simulated in non-overlapping

For peer review only - http://bmjopen.bmj.com/site/about/guidelines.xhtml

subsets of the study population, which included all patients who weren't treated with one or more of statins, RAASi or SGLT2i throughout follow-up. For example, in simulations of statin therapy, patients included in the analysis couldn't have been treated with statins during the entire follow-up period, but they could have been treated with RAASi or SGLT2i. Separate analyses were conducted for monotherapy with each treatment and for all possible combinations of treatment in polytherapy (e.g., any two of the medications of interest or all three medications in combination).

Hazard ratios from several large systematic reviews and meta-analyses,(21–25) which evaluated the effect of statin, RAASi or SGLT2i treatment on all-cause death or MACE in patients with CKD, were used to calculate the probability of five-year survival for those outcomes, simulating the use of guideline-directed pharmacological therapy in the total number of patients untreated with that specific therapy. From the difference between the actual probability of an event in the untreated population, as observed during follow-up, and the estimated probability of five-year survival, $S_{untreated}(5y)$ and $S_{treated}(5y)$, the absolute reduction in risk of an adverse event, Δ_{risk}, was calculated. Using this value, the number needed to treat, NNT, to prevent one adverse event, and the number of prevented deaths (by any cause) and MACE were estimated for each treatment.

$$S_{treated}(5y) = S_{untreated}(5y)^{\exp\left(log_n(HR_{RAASi}) + log_n(HR_{statin}) + log_n(HR_{SGLT2i})\right)};$$
$$\Delta_{risk} = (1 - S_{untreated}(5y)) - (1 - S_{treated}(5y));$$
$$NNT = {}^{1}/\Delta_{risk};$$
$$N_{saved} = {}^{N_{untreated}}/NNT$$

It was assumed that the treatment effect reported by each meta-analysis was replicable in each simulation and that medication adherence was 100% from the date that CKD was identified using laboratory measurements. For combination therapy, it was assumed that there was no deviation from a multiplicative relative effect of using treatments in combination (i.e., no interaction between medications).(26–28)

Patient and public involvement

Patients and the public were not involved in the design, conduct, reporting, or dissemination plans of this study.

RESULTS

Baseline characteristics

In a background population of ~3.8 million residents between the beginning of 2015 and the end of 2020, a total of 99,382 patients without a medical record of CKD were identified as having CKD using laboratory measurements of eGFR and/or UACR. The characteristics of these patients are presented in Table 1 and Table S6. Overall, patients were elderly (median age, 77.7 years; IQR, 71.3-84.2 years), and the prevalence of cardiovascular diseases and diabetes were high. Patients were followed for a median of 3.0 years (IQR, 1.6-4.5 years).

For peer review only - http://bmjopen.bmj.com/site/about/guidelines.xhtml

Table 1. Baseline characteristics of the total study cohort with laboratory-identified chronic kidney disease and that stratified by KDIGO-defined stages of chronic kidney disease

	Total	S3a eGFR 45-59 mL/min/1.73m²	S3b eGFR 30-44 mL/min/1.73m²	S4+S5 eGFR < 30 mL/min/1.73m²	A2 UACR 3-30 g/mol	A3 UACR > 30 g/mol
**N**	99,382	72,407	14,315	1,740	9,549	1,371
**Female, N (%)**	51,198 (51.5%)	37,477 (51.8%)	8,465 (59.1%)	1,180 (67.8%)	3,532 (37.0%)	544 (39.7%)
**Age, median (IQR)**	77.7 (71.3-84.2)	77.9 (72.3-83.6)	83.4 (76.7-88.8)	87.3 (81.5-91.6)	65.3 (56.0-73.2)	62.0 (50.5-70.4)
**Socioeconomic status, N (%)**						
Missing	65,101 (65.5%)	46,897 (64.8%)	10,187 (71.2%)	1,342 (77.1%)	5,757 (60.3%)	918 (67.0%)
High	7,354 (21.5%)	5,847 (22.9%)	881 (21.3%)	85 (21.4%)	497 (13.1%)	44 (9.7%)
Middle	14,154 (41.3%)	10,752 (42.1%)	1,656 (40.1%)	135 (33.9%)	1,444 (38.1%)	167 (36.9%)
Low	12,773 (37.3%)	8,911 (34.9%)	1,591 (38.5%)	178 (44.7%)	1,851 (48.8%)	242 (53.4%)
**Comorbidities, N (%)**						
Heart failure	20,290 (20.4%)	14,511 (20.0%)	4,274 (29.9%)	625 (35.9%)	744 (7.8%)	136 (9.9%)
CHD	26,611 (26.8%)	19,555 (27.0%)	4,543 (31.7%)	555 (31.9%)	1,701 (17.8%)	257 (18.7%)
AMI	11,285 (11.4%)	8,197 (11.3%)	1,999 (14.0%)	255 (14.7%)	725 (7.6%)	109 (8.0%)
Unstable angina	5,498 (5.5%)	4,075 (5.6%)	829 (5.8%)	114 (6.6%)	424 (4.4%)	56 (4.1%)
Stroke	16,717 (16.8%)	12,256 (16.9%)	2,982 (20.8%)	370 (21.3%)	974 (10.2%)	135 (9.8%)
Atrial fibrillation	25,374 (25.5%)	19,035 (26.3%)	4,515 (31.5%)	575 (33.0%)	1,099 (11.5%)	150 (10.9%)
PAD	9,153 (9.2%)	6,649 (9.2%)	1,543 (10.8%)	184 (10.6%)	676 (7.1%)	101 (7.4%)
Diabetes	31,745 (31.9%)	19,510 (26.9%)	4,019 (28.1%)	441 (25.3%)	6,866 (71.9%)	909 (66.3%)
Cancer	30,817 (31.0%)	23,735 (32.8%)	4,652 (32.5%)	571 (32.8%)	1,664 (17.4%)	195 (14.2%)
**Measurements, median (IQR)**						
Systolic BP (mmHg)	140.0 (127.0-150.0)	140.0 (126.0-150.0)	140.0 (125.0-153.0)	140.0 (125.0-155.0)	140.0 (130.0-150.0)	140.0 (130.0-153.0)
BMI (kg/m²)	26.5 (23.5-30.1)	26.2 (23.4-29.7)	25.8 (22.8-29.5)	25.6 (22.6-29.3)	29.0 (25.4-33.1)	29.4 (25.4-33.7)
**Laboratory, median (IQR)**						
P-creatinine (µmol/L)	94.0 (81.0-108.0)	93.0 (82.0-105.0)	116.0 (102.0-133.0)	151.0 (133.0-178.0)	69.0 (59.0-80.0)	70.0 (58.2-83.0)
eGFR (mL/min/1.73m²)	53.7 (46.9-57.9)	54.1 (49.8-57.3)	38.9 (33.8-43.0)	24.5 (20.9-28.3)	78.9 (70.2-90.1)	80.0 (70.6-92.8)
UACR (g/mol)	4.0 (1.0-9.7)	1.3 (0.5-4.6)	2.5 (0.7-9.6)	6.6 (2.1-27.6)	6.6 (4.3-12.0)	54.1 (34.0-90.0)
Sodium (mmol/L)	140.0 (138.0-142.0)	140.0 (138.0-142.0)	140.0 (138.0-142.0)	140.0 (138.0-142.0)	140.0 (138.0-141.0)	139.0 (137.0-141.0)
Potassium (mmol/L)	4.2 (3.9-4.4)	4.2 (3.9-4.4)	4.2 (4.0-4.5)	4.3 (4.0-4.7)	4.1 (3.9-4.3)	4.1 (3.9-4.3)
HbA1c (mmol/mol)	44.0 (39.0-55.0)	42.0 (38.0-51.0)	44.0 (38.0-55.0)	44.0 (38.0-57.0)	53.0 (44.0-67.0)	53.0 (42.0-69.0)
Hemoglobin (g/L)	134.0 (123.0-145.0)	135.0 (124.0-145.0)	128.0 (117.0-139.0)	122.0 (111.0-132.0)	142.0 (131.0-152.0)	140.0 (128.0-153.0)

For peer review only - http://bmjopen.bmj.com/site/about/guidelines.xhtml

**Medications, N (%)**						
Statins	40,732 (41.0%)	29,271 (40.4%)	5,594 (39.1%)	625 (35.9%)	4,611 (48.3%)	631 (46.0%)
RAASi	56,770 (57.1%)	40,626 (56.1%)	8,460 (59.1%)	972 (55.9%)	5,854 (61.3%)	858 (62.6%)
SGLT2i	986 (1.0%)	470 (0.6%)	52 (0.4%)	≤5	412 (4.3%)	49 (3.6%)

Data for baseline demographics and prevalent comorbidities were collected for each patient on the date this study identified their chronic kidney disease (CKD; index date) using laboratory measurements of eGFR and UACR according to the KDIGO clinical practice guidelines. The most recent clinical measurements and laboratory values within the one year prior to the index date are reported. For patients residing in Region Stockholm, the most recent socioeconomic data within the 10 years prior to the index date was collected from the Mosaic system, which applies the principles of geodemography to consumer household and individual data to categorize patients into one of the following three levels: 1) Highest income/education; 2) Medium income/education; and 3) Lowest income/education. Stratification of the patients into the different KDIGO-defined stages of CKD using eGFR (stages S3a, S3b, and S4+S5) and UACR measurements (stages A2 and A3) is based on the median of all measurements in the ≥90-day period required to diagnose CKD. AMI denotes acute myocardial infarction; BMI, body mass index; BP, blood pressure; CHD, coronary heart disease; eGFR, estimated glomerular filtration rate; HbA1c, glycated hemoglobin; IQR, interquartile range; PAD, peripheral artery disease; RAASi, renin-angiotensin aldosterone system inhibitors; SGLT2i, sodium-glucose cotransporter-2 inhibitors; UACR, urinary albumin-creatinine ratio.

For peer review only - http://bmjopen.bmj.com/site/about/guidelines.xhtml

Two out of three with chronic kidney disease remain undiagnosed after five years

After five years from the date that CKD was identified using laboratory measurements, only 33% of the 99,382 patients received a subsequent diagnosis of CKD in healthcare. Of that 33%, the median time to a diagnosis was 1.4 years (IQR, 0.4-2.7 years). The proportion of patients that received a subsequent diagnosis of CKD in healthcare varied between stages of KDIGO-defined CKD, largely irrespective of age (Figure 1 and Figure 2).

In patients with less advanced CKD (KDIGO-defined CKD stage 3a), identified using eGFR measurements, the proportion of patients that received a diagnosis of CKD in healthcare was lower than in those with more advanced CKD (KDIGO stages 4 and 5 CKD) (29% *versus* 82%). A similar pattern was found in those with CKD identified using UACR measurements (KDIGO stage A2 CKD, 34% *versus* KDIGO stage A3 CKD, 62%; Table S7). Analogously, the time to a diagnosis of CKD in healthcare was shorter in patients with more advanced stages of CKD.

Use of guideline-directed pharmacological therapy in relation to a chronic kidney disease diagnosis

The proportions of patients, stratified by the severity of their CKD according to eGFR or UACR measurements, who received a CKD diagnosis in healthcare or who used a guideline-directed pharmacological therapy are presented in Figure 3. Interestingly, around half of the patients were already using statins or RAASi when CKD was identified using laboratory measurements. Use of SGLT2i was negligible, as anticipated.

Use of combination guideline-directed pharmacological therapy might greatly reduce absolute adverse outcome rates

After five years, 21,870 persons died (of any cause), and 22,442 persons experienced a MACE. The proportion of patients that died from any cause or had a MACE increased with each worsening stage of CKD (Figure S2 and Figure S3). Similary, proportions of those adverse outcomes increased with each incremental increase in age (Figure S4 and Figure S5). Findings were similar between men and women.

In simulated monotherapy of statins, RAASi or SGLT2i, the incidences of all-cause death and MACE were reduced by 9-23% (Table 2). When variations of dual therapy were simulated, the incidences of all-cause death and MACE were reduced by 18-36% (Table 2). The greatest reductions in the incidences of all-cause death and MACE were estimated when all three pharmacological treatments were used in combination, preventing 32% of deaths and 42% of MACE (Table 2). If every patient that didn't use an indicated medication for their laboratory-confirmed CKD was treated according to CKD treatment guidelines, 5,535 of the 21,737 deaths and 8,418 of the 22,224 MACE could have potentially been avoided. If everyone that wasn't treated with triple therapy had received it, the number needed to treat to avoid one death and one MACE would be eight and seven, respectively.

For peer review only - http://bmjopen.bmj.com/site/about/guidelines.xhtml
For peer review only

Table 2. Number of adverse events potentially prevented over five years in simulated provision of monotherapy and combination pharmacological therapy to all that were untreated with those corresponding regimens

Treatment	Number of patients without specific treatment regimen, additional possibility to treat	Outcome	Events	Probability of an event in untreated	HR from meta-analysis	Estimated probability of an event	Absolute difference in risk	Number needed to treat	Events prevented
**Eligible for additional onotherapy with:**									
Statins	402	Death	23	0.084	0.92	0.077	0.007	156	3
RAASi	353	Death	11	0.074	0.83	0.062	0.012	82	4
SGLT2i	38760	Death	6415	0.250	0.89	0.226	0.024	42	933
Statins	400	MACE	47	0.199	0.77	0.157	0.042	24	17
RAASi	345	MACE	14	0.083	0.91	0.076	0.007	140	2
SGLT2i	36766	MACE	7477	0.294	0.83	0.251	0.043	23	1584
**Eligible for additional dual therapy with:**									
Statins + RAASi	142	Death	12	0.271	0.76	0.214	0.057	18	8
Statins + SGLT2i	25877	Death	6252	0.359	0.82	0.305	0.054	19	1392
RAASi + SGLT2i	10560	Death	2363	0.327	0.74	0.254	0.073	14	775
Statins + RAASi	146	MACE	10	0.181	0.70	0.131	0.050	20	7
Statins + SGLT2i	26738	MACE	6728	0.368	0.64	0.254	0.114	9	3044
RAASi + SGLT2i	10621	MACE	2344	0.325	0.76	0.257	0.068	15	725
**Eligible for triple therapy**									
All therapies	20420	Death	6661	0.463	0.68	0.345	0.118	8	2420
All therapies	21836	MACE	5604	0.387	0.58	0.247	0.140	7	3039

The outcome death is death by any cause. Treatment will all therapies indicates simulated treatment with a combination of statins, renin-angiotensin aldosterone inhibitors (RAASi), and sodium-glucose cotransporter-2 inhibitors (SGLT2i). The probability of an event in untreated is the probability of an event observed in the given sub-population during follow-up. The estimated probability of event is the probability of an event when each given treatment is simulated using the hazard ratio (HR) reported in the relevant meta-analysis. The absolute difference in risk is the difference in the observed probability of an event and the estimated probability of an event. The number needed to treat is the number of patients that need to be treated

For peer review only - http://bmjopen.bmj.com/site/about/guidelines.xhtml

to prevent one event. MACE denotes major adverse cardiovascular events. RAASi, renin-angiotensin aldosterone inhibitors; SGLT2i, sodium-glucose cotransporter-2 inhibitors.

For peer review only

DISCUSSION

In a background population of ~3.8 million people from Sweden's Stockholm and Skåne regions, this study identified 99,382 patients with undiagnosed CKD in a six-year period that ended in 2020. Of that population, CKD remained undiagnosed by healthcare in two out of three patients five years after CKD could be identified using available laboratory measurements. Those with more advanced CKD were diagnosed faster than those with less severe CKD, seemingly irrespective of age. Use of guideline-directed pharmacological therapy was not dramatically affected by a given CKD diagnosis, seemingly irrespective of the severity of the CKD; and there was very little use of SGLT2i across the entire study population, as anticipated during this observation period. Simulations of guideline-directed pharmacological therapy indicated that there is potential to substantially improve outcomes in CKD, with the greatest projected protective effects resulting from the use of statins, RAASi and SGLT2i in combination.

What opportunities are currently lost in chronic kidney disease?

A CKD diagnosis would be anticipated to lead to several changes in care, such as more structured follow-up, changed dosing of several drug classes, and more preventive measures taken at contrast radiological examinations, to name but a few.

No striking changes to treatment regimens were observed around the time of a diagnosis of CKD in healthcare, suggesting that clinical practice didn't always follow the clinical practice guidelines informing practitioners during the study period, such as those published by KDIGO in 2012 and 2013,(8,9) and national guidelines and recommendations. Indeed, the use of such pharmacological treatments is pertinent to this study's population, where the proportion of patients that was affected by MACE was far greater than the proportion that died of non-cardiovascular causes. The underuse of guideline-directed pharmacological therapy is consistent with recent large, cohort studies, which also reported that only small proportions of patients with CKD were using statins, RAASi or SGLT2i.(1,10) Given that SGLT2i have only recently been included in clinical practice guidelines after the study period,(7,11,12) low use of these agents was in line with expectations. Hence, our data clearly show that opportunities are lost in both the diagnosis and treatment of CKD.

It should be noted that diagnosing and treating CKD is likely less incentivized in Sweden than in many other countries. A CKD diagnosis has smaller effects on rewards for healthcare units in Sweden, where a government-funded healthcare system covers all citizens, than it would have under insurance/claims systems.(29) Further, some other countries with similar healthcare systems have much stronger encouragement for registering a CKD diagnosis and providing evidence-based CKD care. The United Kingdom, for example, had a focus on registering CKD diagnoses and providing evidence-based preventive care since 2006, as part of the primary care Quality and Outcomes Framework.(30)

What are the windows of opportunity in chronic kidney disease?

Simulations of pharmacological treatments indicated that, with greater adherence to guidelines by the caring physician and adequate medication adherence by the patient, there

For peer review only - http://bmjopen.bmj.com/site/about/guidelines.xhtml

is significant potential to improve outcomes in CKD. Addressing cardiovascular disease risk is central to CKD treatment.(7) Lowering of blood pressure and lipids in patients with CKD has robust evidence for the prevention of both cardiovascular disease events and death;(21–23) and evidence is accumulating that SGLT2i reduces the risk of MACE and all-cause mortality in all patients with CKD, irrespective of whether they have diabetes or not.(11,12,31) Combinations of these treatments are likely to have multiplicative protective effects.(26,27) Therefore, there is little reason, apart from side effects, to not use all three treatments in combination from the time of CKD diagnosis. On the contrary, RAASi and SGLT2i seem to have additive effects when used in combination.

It's likely that the laboratory analyses used for CKD definitions in the present study are routinely performed for an array of conditions and complaints, but unless the caring physician is specifically looking for kidney disease, the historic data needed to make a CKD diagnosis may be overlooked. Although our findings are exclusively based on Swedish health care data, the low rate of CKD diagnosis in patients who fulfill CKD criteria has been described in numerous other health care systems globally.(1) While greater use of evidence-based treatment is likely to have positive effects, the potential to improve outcomes in CKD can only be fully realized if deficiencies in the diagnosis of CKD are also addressed. Future research might trial systems where eGFR and UACR values in databases are automatically monitored, notifying the caring physicians if a patient presents abnormal measurements meeting the criteria for CKD.

This concept has already been implemented in acute kidney injury, where electronic alert systems monitor electronic health records.(32,33) An automated system, such as that, may be the most reliable way to address the issue of human oversight, the likely reason so many cases of CKD went undiagnosed. Indeed, automated systems could theoretically be applied in multiple scenarios, from small databases within private health clinics to large national or regional databases, providing the capacity for continuous population-wide opportunistic screening; as compared to the current system, in which screening is only recommended in high-risk populations (i.e., diabetes, hypertension and previously known cardiovascular disease).(34)

Strengths and limitations

This study had access to health registries and electronic health records detailing most incidences of healthcare and medication use by residents in two of Sweden's largest regions, representing over a third of the national population. Despite its strength in its capacity to identify patients relevant to the study aims and to characterize the subsequent, large study population, several limitations must be acknowledged.

The identification of undiagnosed CKD was dependent on patients providing samples for laboratory analyses. Since only persons with suspected or known other disease have provided the samples necessary to make a laboratory-based assessment of CKD status, it is likely that this study underestimates the number of patients with undiagnosed CKD. It is possible that some diagnosing has taken place outside of the utilized data source. This would instead lead to an underestimation of the number of patients with a diagnosis of CKD. The follow-up period varied between patients, and in some cases, patients may have been enrolled towards the end of the inclusion period, leaving little opportunity for a diagnosis of CKD in healthcare. However, the methods used take person-time and

For peer review only - http://bmjopen.bmj.com/site/about/guidelines.xhtml

censoring into account. It must also be acknowledged that this study couldn't assess if patients from a specific socioeconomic background or ethnicity were disproportionally affected by the deficiencies in CKD-related healthcare.

Simulations of guideline-directed pharmacological therapy assumed that treatment was initiated at the time CKD was identified using laboratory measurements and that the adherence to treatment would be 100%. While indicating the full preventive potential, such efficiency and success in treatment is unlikely in routine clinical practice. The simulations assumed that the treatment effects observed in meta-analyses of each medication of interest were replicable in this study's untreated populations and that conjoint effects on outcomes did not deviate from multiplicativity, which has been demonstrated for RAASi and statins, but not robustly for SGLT2i in relation to the other two drug classes.

Conclusion

Chronic kidney disease remained undiagnosed in two out of three patients over half a decade in routine healthcare, despite readily available data indicating its development. This highlights the need for automated systems that continuously monitor electronic health records and alert physicians when CKD has developed in a patient; eliminating any reliance on the physician to, firstly, decide to search for the disease and to then make a diagnosis. Those who do receive a diagnosis get insufficient guideline-directed pharmacological therapy with statins, RAASi, and SGLT2i, collectively potentiating a situation where CKD won't be addressed in healthcare until more advanced disease develops. Despite these lost opportunities, this study indicates that there is significant potential to substantially improve outcomes in CKD with stricter adherence to clinical practice guidelines by both the treating physicians and the patients.

For peer review only - http://bmjopen.bmj.com/site/about/guidelines.xhtml

AUTHOR CONTRIBUTIONS

J.S., A.N., S.K., J.B, S.G., T.C:, M.K.S., and J.Ä. developed the concept of the study. S.G and T.C performed all data curation and statistical analyses. J.S., A.N., S.K., S.G., T.C:, M.K.S., and J.Ä. participated in data interpretation. J.S. drafted the original manuscript, with critical intellectual input from A.N., S.K., J.B, S.G., T.C:, M.K.S., and J.Ä.

ACKNOWLEDGEMENTS

The authors thank Jordan Loader PhD, of Sence, Uppsala, Sweden, for providing medical writing support, which was funded by AstraZeneca, Stockholm, Sweden, in accordance with Good Publication Practice (GPP3) guidelines (http://www.ismpp.org/gpp3). The authors also thank Susanna Jerström and Pål Hasvold from AstraZeneca for valuable comments on the manuscript.

FUNDING

The study was fully sponsored by AstraZeneca.

COMPETING INTERESTS

J.S. reports stock ownership in Anagram kommunikation AB and Symptoms Europe AB. A.N. has participated as a consultant on advisory board committees and educational activities with Astra Zeneca, Novo Nordisk, Boehringer Ingelhim and Eli Lilly. S.K. has participated as a consultant on advisory board committees and educational activities with AstraZeneca and Novo Nordisk. J.B. is an employee of AstraZeneca. T.C. is an employee and stockowner in Sence Research AB (an independent company in epidemiology and outcomes research) that received funding from AstraZeneca for statistical analysis within this research project. J.Ä. has received payment/honoraria from AstraZeneca and Novartis; participated on a data safety monitoring/advisory board for AstraZeneca, Astella and Boehringer Ingelheim. The other authors report no competing interests.

For peer review only

REFERENCES

1. Sundström J, Bodegard J, Bollmann A, Vervloet MG, Mark PB, Karasik A, et al. Prevalence, outcomes, and cost of chronic kidney disease in a contemporary population of 2·4 million patients from 11 countries: The CaReMe CKD study. The Lancet Regional Health - Europe. 2022 Sep 1;20:100438.
2. GBD Chronic Kidney Disease Collaboration. Global, regional, and national burden of chronic kidney disease, 1990-2017: a systematic analysis for the Global Burden of Disease Study 2017. Lancet. 2020 Feb 29;395(10225):709–33.
3. Hill NR, Fatoba ST, Oke JL, Hirst JA, O'Callaghan CA, Lasserson DS, et al. Global Prevalence of Chronic Kidney Disease - A Systematic Review and Meta-Analysis. PLoS One. 2016;11(7):e0158765.
4. Chen TK, Knicely DH, Grams ME. Chronic Kidney Disease Diagnosis and Management: A Review. JAMA. 2019 Oct 1;322(13):1294–304.
5. Saran R, Robinson B, Abbott KC, Agodoa LYC, Bhave N, Bragg-Gresham J, et al. US Renal Data System 2017 Annual Data Report: Epidemiology of Kidney Disease in the United States. American Journal of Kidney Diseases. 2018 Mar;71(3):A7.
6. Saran R, Robinson B, Abbott KC, Bragg-Gresham J, Chen X, Gipson D, et al. US Renal Data System 2019 Annual Data Report: Epidemiology of Kidney Disease in the United States. American Journal of Kidney Diseases. 2020 Jan;75(1):A6–7.
7. de Boer IH, Khunti K, Sadusky T, Tuttle KR, Neumiller JJ, Rhee CM, et al. Diabetes Management in Chronic Kidney Disease: A Consensus Report by the American Diabetes Association (ADA) and Kidney Disease: Improving Global Outcomes (KDIGO). Diabetes Care. 2022 Oct 3;45(12):3075–90.
8. Levin A, Stevens PE. Summary of KDIGO 2012 CKD Guideline: behind the scenes, need for guidance, and a framework for moving forward. Kidney Int. 2014 Jan;85(1):49–61.
9. Wanner C, Tonelli M, Kidney Disease: Improving Global Outcomes Lipid Guideline Development Work Group Members. KDIGO Clinical Practice Guideline for Lipid Management in CKD: summary of recommendation statements and clinical approach to the patient. Kidney Int. 2014 Jun;85(6):1303–9.
10. Tuttle KR, Alicic RZ, Duru OK, Jones CR, Daratha KB, Nicholas SB, et al. Clinical Characteristics of and Risk Factors for Chronic Kidney Disease Among Adults and Children: An Analysis of the CURE-CKD Registry. JAMA Netw Open. 2019 Dec 2;2(12):e1918169.
11. Heerspink HJL, Stefánsson BV, Correa-Rotter R, Chertow GM, Greene T, Hou FF, et al. Dapagliflozin in Patients with Chronic Kidney Disease. N Engl J Med. 2020 Oct 8;383(15):1436–46.
12. The EMPA-KIDNEY Collaborative Group, Herrington WG, Staplin N, Wanner C, Green JB, Hauske SJ, et al. Empagliflozin in Patients with Chronic Kidney Disease. N Engl J Med. 2023 Jan 12;388(2):117–27.
13. Anell A. The public-private pendulum--patient choice and equity in Sweden. N Engl J Med. 2015 Jan 1;372(1):1–4.
14. Ludvigsson JF, Otterblad-Olausson P, Pettersson BU, Ekbom A. The Swedish personal identity number: Possibilities and pitfalls in healthcare and medical research. European Journal of Epidemiology. 2009;24(11):659–67.

For peer review only - http://bmjopen.bmj.com/site/about/guidelines.xhtml

15. World Health Organization. WHO Collaborating Centre for Drug Statistics Methodology. ATC/DDD Index 2017. Available from: https://www.whocc.no/. Accessed December 9, 2022.
16. World Health Organization. International classification of disease and related health problems. Available from: http://www.who.int/classifications/icd/en/. Accessed December 9, 2022.
17. Nordic Medico-Statistical Committee. NOMESCO Classification of Surgical Procedures. 2010. Available from: https://norden.diva-portal.org/smash/get/diva2:970547/FULLTEXT01.pdf. Accessed December 9, 2022.
18. Nyman U, Grubb A, Larsson A, Hansson LO, Flodin M, Nordin G, et al. The revised Lund-Malmö GFR estimating equation outperforms MDRD and CKD-EPI across GFR, age and BMI intervals in a large Swedish population. Clin Chem Lab Med. 2014 Jun;52(6):815–24.
19. R Development Core Team Core Team. R: A language and environment for statistical computing. (2011).
20. Carstensen B, Plummer M. Using Lexis Objects for Multi-State Models in R. Journal of Statistical Software. 2011 Jan 4;38:1–18.
21. Hou W, Lv J, Perkovic V, Yang L, Zhao N, Jardine MJ, et al. Effect of statin therapy on cardiovascular and renal outcomes in patients with chronic kidney disease: a systematic review and meta-analysis. Eur Heart J. 2013 Jun;34(24):1807–17.
22. Balamuthusamy S, Srinivasan L, Verma M, Adigopula S, Jalandhara N, Jalandara N, et al. Renin angiotensin system blockade and cardiovascular outcomes in patients with chronic kidney disease and proteinuria: a meta-analysis. Am Heart J. 2008 May;155(5):791–805.
23. Qin Y, Chen T, Chen Q, Lv JY, Qi N, Wu C, et al. The effect of angiotensin-converting enzyme inhibitor/angiotensin receptor blocker use on mortality in patients with chronic kidney disease: a meta-analysis of observational studies. Pharmacoepidemiol Drug Saf. 2016 May;25(5):503–11.
24. Kamdar A, Sykes R, Morrow A, Mangion K, Berry C. Cardiovascular outcomes of glucose lowering therapy in chronic kidney disease patients: a systematic review with meta-analysis. Rev Cardiovasc Med. 2021 Dec 22;22(4):1479–90.
25. Kaze AD, Zhuo M, Kim SC, Patorno E, Paik JM. Association of SGLT2 inhibitors with cardiovascular, kidney, and safety outcomes among patients with diabetic kidney disease: a meta-analysis. Cardiovascular Diabetology. 2022 Mar 23;21(1):47.
26. Sundström J, Gulliksson G, Wirén M. Synergistic effects of blood pressure-lowering drugs and statins: systematic review and meta-analysis. BMJ Evid Based Med. 2018 Apr;23(2):64–9.
27. Kanukula R, Esam H, Sundström J, Rodgers A, Salam A. Does Co-administration of Antihypertensive Drugs and Statins Alter Their Efficacy and Safety? A Systematic Review and Meta-analysis of Randomized Controlled Trials. J Cardiovasc Pharmacol. 2019 Jun;73(6):352–8.
28. Wald DS, Law M, Morris JK, Bestwick JP, Wald NJ. Combination therapy versus monotherapy in reducing blood pressure: meta-analysis on 11,000 participants from 42 trials. Am J Med. 2009 Mar 1;122(3):290–300.
29. Tangri N, Franzén S, Peach EJ, Barone S, Kushner PR. REVEAL CKD: Estimated Glomerular Filtration Rate (eGFR) Decline Before and After a CKD Diagnosis Among

For peer review only - http://bmjopen.bmj.com/site/about/guidelines.xhtml

Patients With CKD Stage 3. Presented at the American Society of Nephrology Kidney Week 2022, Orlando, FL, USA, November 3–6. 2022;
30. Stevens PE, O'Donoghue DJ. The UK model for system redesign and chronic kidney disease services. Semin Nephrol. 2009 Sep;29(5):475–82.
31. Wheeler DC, Stefánsson BV, Jongs N, Chertow GM, Greene T, Hou FF, et al. Effects of dapagliflozin on major adverse kidney and cardiovascular events in patients with diabetic and non-diabetic chronic kidney disease: a prespecified analysis from the DAPA-CKD trial. Lancet Diabetes Endocrinol. 2021 Jan;9(1):22–31.
32. Kashani KB. Automated acute kidney injury alerts. Kidney Int. 2018 Sep;94(3):484–90.
33. Breighner CM, Kashani KB. Impact of e-alert systems on the care of patients with acute kidney injury. Best Pract Res Clin Anaesthesiol. 2017 Sep;31(3):353–9.
34. Nationellt programområde för njur-och urinvägssjukdomar. Nationellt vårdprogram för kronisk njursjukdom. Version 2, 2022. Available from: https://d2flujgsl7escs.cloudfront.net/external/Nationellt_vardprogram_for_kronisk_njursjukdom.pdf. Accessed January 15, 2023.

For peer review only

FIGURE LEGENDS

Figure 1. Time to first chronic kidney disease (CKD) diagnosis in healthcare after CKD was identified using measurements of estimated glomerular filtration rate (eGFR)

Kaplan-Meier curves. Patients are stratified by the KDIGO-defined stage of their CKD and by age group. S3a indicates eGFR 45-59 mL/min/1.73m^2; S3b, eGFR 30-44 mL/min/1.73m^2; S4, eGFR 15-29 mL/min/1.73m^2; S5, eGFR < 15 mL/min/1.73m^2.

Figure 2. Time to first chronic kidney disease (CKD) diagnosis in healthcare after CKD was identified using measurements of urinary albumin-creatinine ratio (UACR)

Kaplan-Meier curves. Patients are stratified by the KDIGO-defined stage of their CKD and by age group. All patients from the total cohort whose CKD could be identified using abnormal UACR measurements according to the KDIGO guidelines were included in these analyses, regardless of whether measurements of estimated glomerular filtration rate were available. A2 indicates UACR 3-30 g/mol; A3, UACR > 30 g/mol.

Figure 3. Multi-state models presenting the proportions of patients diagnosed or not diagnosed with chronic kidney disease (CKD) in healthcare that used or didn't use guideline-directed pharmacological therapy

Use of **A)** statins, **B)** renin-angiotensin aldosterone system inhibitors (RAASi), and **C)** sodium-glucose cotransporter-2 inhibitors (SGLT2i) were assessed. Patients are stratified by the KDIGO-defined stage of their CKD, identified using estimated glomerular filtration rate (Stages S3a, S3b, and S4+S5) and urinary albumin-creatinine ratio (Stages A2 and A3).

For peer review only - http://bmjopen.bmj.com/site/about/guidelines.xhtml

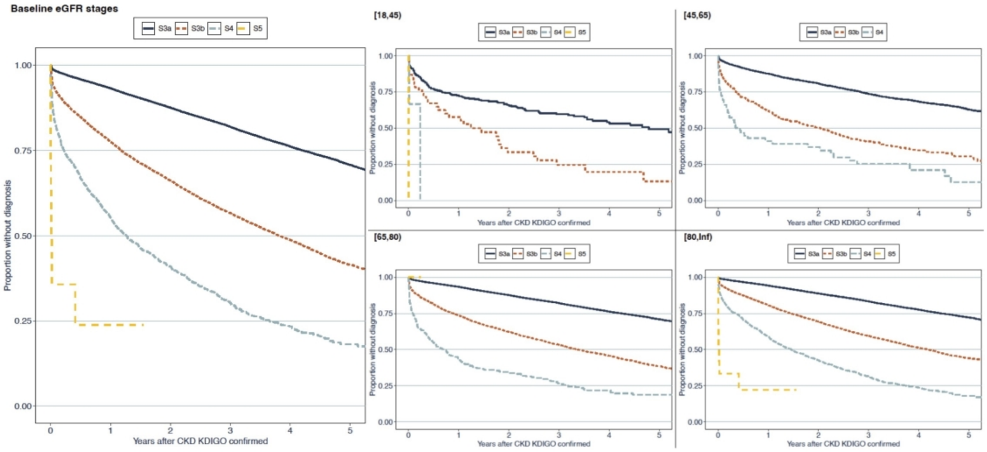

Figure 1. Time to first chronic kidney disease (CKD) diagnosis in healthcare after CKD was identified using measurements of estimated glomerular filtration rate (eGFR)
Kaplan-Meier curves. Patients are stratified by the KDIGO-defined stage of their CKD and by age group. S3a indicates eGFR 45-59 mL/min/1.73m2; S3b, eGFR 30-44 mL/min/1.73m2; S4, eGFR 15-29 mL/min/1.73m2; S5, eGFR < 15 mL/min/1.73m2.

236x111mm (300 x 300 DPI)

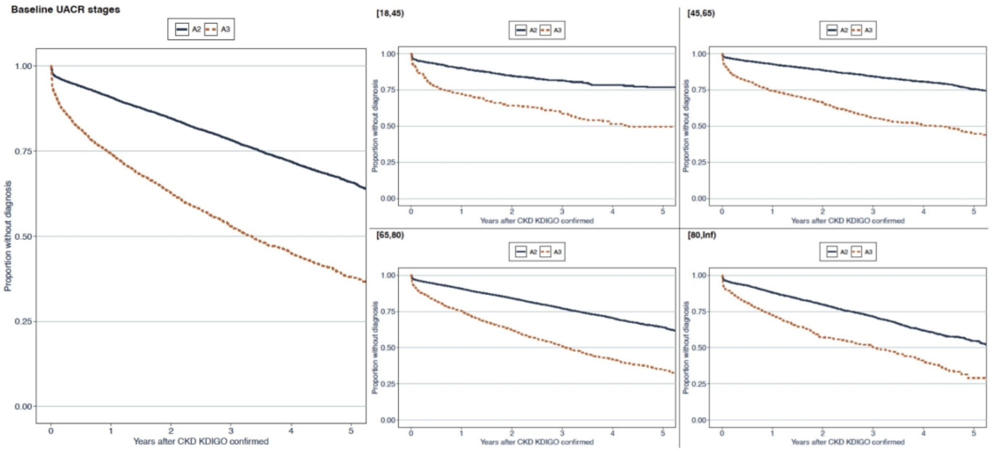

Figure 2. Time to first chronic kidney disease (CKD) diagnosis in healthcare after CKD was identified using measurements of urinary albumin-creatinine ratio (UACR)

Kaplan-Meier curves. Patients are stratified by the KDIGO-defined stage of their CKD and by age group. All patients from the total cohort whose CKD could be identified using abnormal UACR measurements according to the KDIGO guidelines were included in these analyses, regardless of whether measurements of estimated glomerular filtration rate were available. A2 indicates UACR 3-30 g/mol; A3, UACR > 30 g/mol.

229x105mm (300 x 300 DPI)

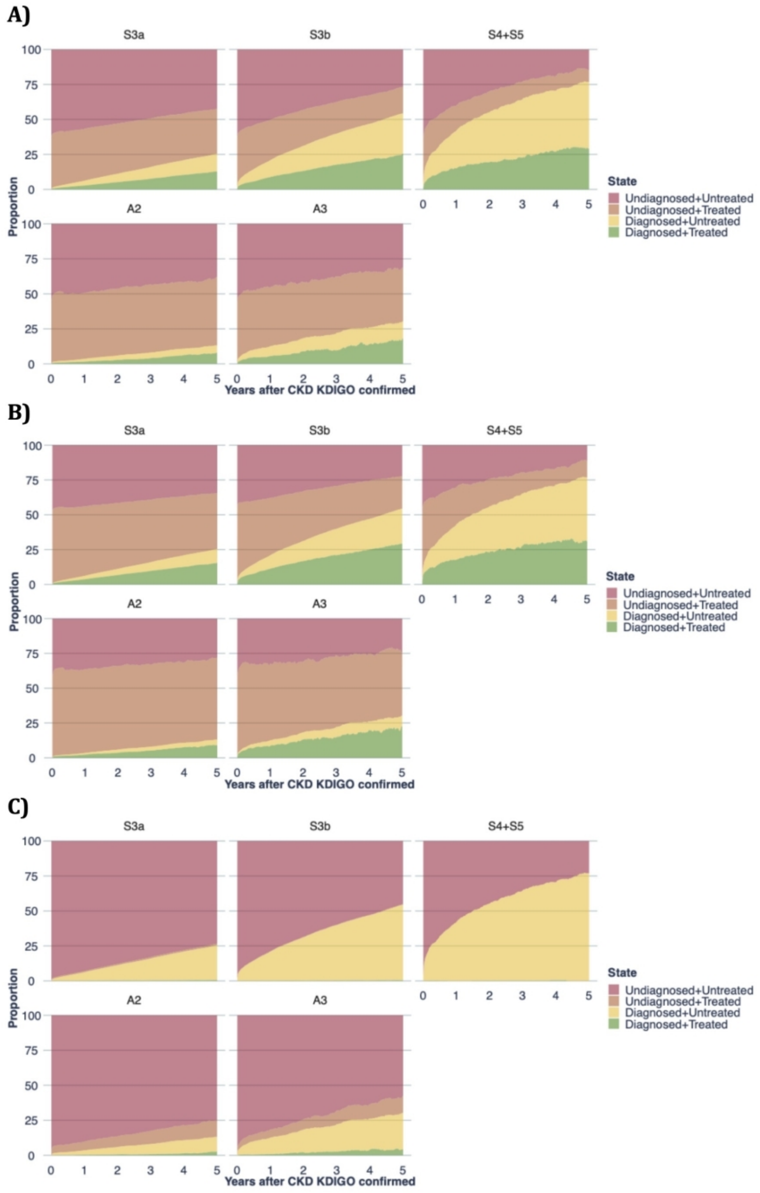

Figure 3. Multi-state models presenting the proportions of patients diagnosed or not diagnosed with chronic kidney disease (CKD) in healthcare that used or didn't use guideline-directed pharmacological therapy Use of A) statins, B) renin-angiotensin aldosterone system inhibitors (RAASi), and C) sodium-glucose cotransporter-2 inhibitors (SGLT2i) were assessed. Patients are stratified by the KDIGO-defined stage of their CKD, identified using estimated glomerular filtration rate (Stages S3a, S3b, and S4+S5) and urinary albumin-creatinine ratio (Stages A2 and A3).

145x228mm (300 x 300 DPI)

Lost opportunities in chronic kidney disease

Supplemental Material

Contents

Supplemental Methods. The CELOSIA database....................2

Table S1. Meta-analyses that assessed the effect of first-line pharmacological treatments on the risk of major adverse cardiovascular events in patients with chronic kidney disease, with or without diabetes....................3

Table S2. Inclusion criteria for the CELOSIA database from which the study sample was extracted....................4

Table S3. Stages of chronic kidney disease diagnosed using measurements of eGFR and UACR according to the KDIGO clinical practice guidelines....................6

Table S4. International Classification of Diseases (ICD)-10 codes used to search for diagnoses of CKD, prevalent comorbidities, and outcomes of interest....................7

Table S5. Anatomical Therapeutic Chemical (ATC) classification system codes used to search for treatments of interest in this study's population....................8

Table S6. Baseline characteristics of the total study cohort stratified by age group....................9

Table S7. Proportions of patients diagnosed with chronic kidney disease in healthcare within five years of the development of the disease....................11

Figure S1. Participant flow....................12

Figure S2. Multi-state models presenting the proportions of patients diagnosed or not diagnosed with chronic kidney disease (CKD) in healthcare who received or didn't receive guideline-directed pharmacological therapy, including the terminal, absorbing outcome, all-cause death....................13

Figure S3. Multi-state models presenting the proportions of patients diagnosed or not diagnosed with chronic kidney disease (CKD) in healthcare who received or didn't receive guideline-directed pharmacological therapy, including the terminal, absorbing outcomes, major adverse cardiovascular events (MACE) and non-cardiovascular disease (CVD) death....................15

Figure S4. Multi-state models presenting the proportions of patients diagnosed or not diagnosed with chronic kidney disease (CKD) in healthcare who received or didn't receive guideline-directed pharmacological therapy, including the terminal, absorbing outcome, all-cause death....................17

Figure S5. Multi-state models presenting the proportions of patients diagnosed or not diagnosed with chronic kidney disease (CKD) in healthcare who received or didn't receive guideline-directed pharmacological therapy, including the terminal, absorbing outcomes, major adverse cardiovascular events (MACE) and non-cardiovascular disease (CVD) death....................19

For peer review only - http://bmjopen.bmj.com/site/about/guidelines.xhtml

References...21

For peer review only

For peer review only - http://bmjopen.bmj.com/site/about/guidelines.xhtml

Supplemental Methods. The CELOSIA database

The CELOSIA database, from which this present study's sample was extracted, includes all Swedish residents aged ≥18 years who were diagnosed with chronic kidney disease (CKD), heart failure or diabetes mellitus between January 1st, 2000, and December 31st, 2020.

Patients were identified from several sources with national coverage, including the Swedish Prescribed Drug Registry, the National Patient Registry, and the National Death Registry. The Swedish Prescribed Drug Registry logs each resident's filled drug prescriptions according to the Anatomical Therapeutic Chemical [ATC] classification system.[1] The National Patient Registry and the National Death Registry records diagnoses, surgical procedures, and causes of death using the International Classification of Diseases [ICD][2] system and the Nordic Medico-Statistical Committee Classification of Surgical Procedure system.[3]

Patients were also identified from several data sources with regional coverage, including the Healthcare Data Warehouse of Region Stockholm, the Healthcare Data Warehouse of Region Skåne Melior, and the Profdoc Medical Office of Region Skåne. Additionally, the CELOSIA database utilized electronic health records from Region Stockholm and Region Skåne to diagnose patients with CKD using laboratory measurements.

A complete list of the ATC codes, ICD-10 codes, procedure codes, and laboratory values used to identify these patients is available in Supplemental Table 2. Patients were required to have a 12-digit personal identity number, an identity number unique to all Swedish residents,[4] enabling data to be linked between the different sources.

The total population with data available for the present study was ~3.8 million people, corresponding to 36% of the Swedish population.

For peer review only

Table S1. Meta-analyses that assessed the effect of first-line pharmacological treatments on the risk of major adverse cardiovascular events in patients with chronic kidney disease, with or without diabetes

Study	Drug	Statistic	All-cause mortality	MACE	CVD mortality	Myocardial infarction	Stroke	Hospitalization for HF
Qin et al., 2016[5]	RAASi	HR (95%CI)	0.83 (0.78-0.87)					
Balamuthusamy et al., 2008[6]	RAASi	RR (95%CI)	1.03 (0.92-1.04)	0.91 (0.85-0.98)	1.12 (0.95-1.32)	0.82 (0.67-1.00)	1.02 (0.81-1.28)	0.78 (0.66-0.92)
Xie et al., 2016[7]	ACEi	OR (95%CI)	0.87 (0.74-1.01)	0.82 (0.71-0.92)	0.88 (0.72-1.09)			
Zhang et al., 2020[8]	ACEi	OR (95%CI)	0.77 (0.66-0.91)	0.73 (0.64-0.84)	0.73 (0.63-0.86)			
Ninomiya et al., 2013[9]	ACEi	HR (95%CI)		0.81 (0.73-0.90)				
Xie et al., 2016[7]	ARBs	OR (95%CI)	0.99 (0.78-1.21)	0.76 (0.62-0.89)	1.12 (0.80-1.58)			
Zhang et al., 2020[8]	ARBs	OR (95%CI)	1.01 (0.82-1.25)	0.83 (0.70-0.98)	1.16 (0.88-1.53)			
Major et al., 2015[10]	Statins	RR (95%CI)	0.66 (0.49-0.88)	0.59 (0.48-0.72)			0.56 (0.28-1.13)	
Su et al., 2016[11]	Statins	OR (95%CI)		0.69 (0.61-0.79)				
Upadhyay et al., 2012[12]	Statins	RR (95%CI)	0.88 (0.79-0.98)	0.75 (0.68-0.84)				
Strippoli et al., 2008[13]	Statins	RR (95%CI)	0.81 (0.74-0.89)		0.80 (0.70-0.90)			
Palmer et al., 2014[14]	Statins	RR (95%CI)	0.81 (0.74-0.88)	0.76 (0.73-0.80)	0.78 (0.68-0.89)	0.55 (0.42-0.72)	0.61 (0.38-0.98)	
Hou et al., 2013[15]	Statins	RR (95%CI)	0.92 (0.85-0.99)	0.77 (0.70-0.85)	0.91 (0.84-0.99)		0.79 (0.56-1.12)	
Zhang et al., 2014[16]	Statins	RR (95%CI)	0.79 (0.72-0.86)	0.76 (0.72-0.80)	0.83 (0.73-0.93)	0.66 (0.52-0.83)	0.70 (0.57-0.85)	
Barylski et al., 2013[17]	Statins	RR (95%CI)	0.66 (0.55-0.79)	0.55 (0.40-0.75)	0.69 (0.55-0.78)		0.66 (0.50-0.88)	
Yamada et al., 2021[18]	SGLT-2i	RR (95%CI)		0.87 (0.76-1.00)				
Salah et al., 2021[19]	SGLT-2i	HR (95%CI)	0.82 (0.67-1.00)		0.84 (0.68-1.03)	0.72 (0.54-0.97)	0.67 (0.33-1.36)	0.61 (0.48-0.77)
Lin et al., 2021[20]	SGLT-2i	RR (95%CI)		0.84 (0.82-0.97				
Kamdar et al., 2021[21]	SGLT-2i	HR (95%CI)	0.89 (0.81-0.98)		0.90 (0.79-1.02)	0.82 (0.64-1.05)		0.69 (0.63-0.75)
D'Andrea et al., 2020[22]	SGLT-2i	HR (95%CI)		0.82 (0.69-0.97)				
Staplin et al., 2021[23]	SGLT-2i	RR (95%CI)	0.94 (0.75-1.12)	0.84 (0.76-0.93)	0.84 (0.73-0.97)			
Malik et al., 2020[24]	SGLT-2i	HR (95%CI)		0.80 (0.69-0.92)	0.88 (0.71-1.09)	0.78 (0.62-0.97)	0.83 (0.62-1.11)	0.61 (0.47-0.77)
Cao et al., 2021[25]	SGLT-2i	HR (95%CI)	0.83 (0.55-1.27)	0.75 (0.60-0.93)	0.89 (0.60-1.30)			0.74 (0.55-0.99)
Kaze et al., 2022[26]	SGLT-2i	HR (95%CI)	0.86 (0.77-0.96)	0.83 (0.75-0.93)	0.84 (0.74-0.96)	0.78 (0.67-0.92)	0.76 (0.59-0.97)	0.62 (0.55-0.71)
Yang et al., 2022[27]	SGLT-2i	OR (95%CI)	0.79 (0.66-0.93)		0.80 (0.65-0.98)			0.57 (0.45-0.72)

ACEi denotes angiotensin converting enzyme inhibitors; ARBs, angiotensin receptor blockers; CI, confidence interval; CVD, cardiovascular disease; HF, heart failure; HR, hazard ratio; MACE, major adverse cardiovascular events; OR, odds ratio; RAASi, renin-angiotensin aldosterone system inhibitors; RR, risk ratio; SGLT-2i, sodium-glucose cotransporter-2 inhibitors.

For peer review only - http://bmjopen.bmj.com/site/about/guidelines.xhtml

Table S2. Inclusion criteria for the CELOSIA database from which the study sample was extracted

Inclusion criteria (any of)	Description
ICD10: I50	Heart failure
ICD10: I11.0	Hypertensive heart disease with (congestive) heart failure
ICD10: I13.0	Hypertensive heart and renal disease with (congestive) heart failure
ICD10: I13.2	Hypertensive heart and renal disease with both (congestive) heart failure and renal failure
ICD10: I25.5	Ischaemic cardiomyopathy
ICD10: I42.0	Dilated cardiomyopathy
ICD10: I42.6	Alcoholic cardiomyopathy
ICD10: I42.9	Cardiomyopathy, unspecified
ICD10: I43.1	Cardiomyopathy in metabolic diseases
ICD10: Z99.4	Dependence on artificial heart
Procedure: FQA	Transplantation of heart
Procedure: FQB	Transplantation of heart and lung
Procedure: FPE26	Implantation of transvenous cardiac pacemaker with biventricular electrodes
ATC: C01CX08	Levosimendan
BNP >100ng/L	Laboratory value
NT-proBNP >300ng/L	Laboratory value
ICD-10: E10.2	Type I diabetes mellitus with renal complications
ICD-10: E11.2	Type II diabetes mellitus with renal complications
ICD-10: E12.2	Malnutrition-related diabetes mellitus with renal complications
ICD-10: E13.2	Other specified diabetes mellitus with renal complications
ICD-10: E14:2	Unspecified diabetes mellitus with renal complications
ICD-10: N08.3	Glomerular disorders in diabetes mellitus
ICD-10: N17	Acute renal failure
ICD-10: N18	Chronic kidney failure
ICD-10: N19	Unspecified kidney failure
ICD-10: I12.0	Glomerular disorders in diabetes mellitus
ICD-10: I12.9	Hypertensive renal disease without renal failure
ICD-10: I13.1	Hypertensive heart and renal disease with renal failure
ICD-10: I13.9	Hypertensive heart and renal disease, unspecified
ICD-10: Z49.1	Extracorporeal dialysis
ICD-10: Z49.2	Other dialysis
ICD-10: Z99.2	Dependence on renal dialysis
Procedure: JAK10	Laparotomy and insertion of peritoneal dialysis catheter
Procedure: TJA33	Percutaneous introduction of peritoneal dialysis catheter
Procedure: TJA35	Removal of peritoneal dialysis catheter
Procedure: DJ008	Laparoscopic dialysis catheter insertion
Procedure: DR013	Initiation of continuos ambulatory peritoneal dialysis (CAPD)
Procedure: DR014	Hemodiafiltration (HDF)
Procedure: DR015	Hemodialysis, acute
Procedure: DR016	Hemodialysis, chronic

For peer review only - http://bmjopen.bmj.com/site/about/guidelines.xhtml

Procedure: DR017	Hemofiltration
Procedure: DR023	Peritonealdialysis, acute
Procedure: DR024	Peritonealdialysis, chronic
Procedure: DR055	Citrate dialysis
Procedure: DR056	Heparin free dialysis
Procedure: DR060	Home hemodialysis control
Procedure: DR061	Home hemodialysis start
Procedure: KAS10	Allogenic transplantation of kidney from cadaver donor
Procedure: KAS20	Allogenic transplantation of kidney from living donor
Procedure: QF006	Peritoneal dialysis
eGFR <60 mL/min/1.73m^2	Laboratory value
U-albumin/creatinine ratio > 3 mg/mmol	Laboratory value
ICD-10: E10	Type I diabetes mellitus
ICD-10: E11	Type II diabetes mellitus
ICD-10: E12	Malnutrition-related diabetes mellitus
ICD-10: E13	Other specified diabetes mellitus
ICD-10: E14	Unspecified diabetes mellitus
ATC: A10	Drugs used in diabetes
HbA1c > 48 mmol/L	Laboratory value

For peer review only

For peer review only - http://bmjopen.bmj.com/site/about/guidelines.xhtml

Table S3. Stages of chronic kidney disease diagnosed using measurements of eGFR and UACR according to the KDIGO clinical practice guidelines

**eGFR-defined stages**		
**Stage**	**eGFR thresholds (mL.min.1.73m²)**	**Abnormal**
1	≥ 90	No
2	60-89	No
3a	45-59	Yes
3b	30-44	Yes
4	15-29	Yes
5	GFR < 15	Yes
**Albuminuria-defined stages**		
**Stage**	**UACR thresholds (g/mol)**	**Abnormal**
A1	UACR < 3	No
A2	UACR 3-30	Yes
A3	UACR > 30	Yes

eGFR denotes estimated glomerular filtration rate; UACR, urinary albumin-creatinine ratio.

For peer review only - http://bmjopen.bmj.com/site/about/guidelines.xhtml

Table S4. International Classification of Diseases (ICD)-10 codes used to search for diagnoses of CKD, prevalent comorbidities, and outcomes of interest

Condition	ICD-10 codes
**CKD diagnosis and exclusions**	
CKD (broad)	N17-N19, I12.0, I12.9, I13.1-I13.2, E10.2, E11.2, E12.2, E13.2, E14.2, N00-N08, N10-N16, Z49, Z99.2
CKD (narrow)	N17-N19, I12.0, I12.9, I13.1-I13.2, E10.2, E11.2, E12.2, E13.2, E14.2, N083, Z49.1-Z49.2, Z99.2
Kidney donation	Z52.4
**Comorbidities**	
Heart failure	I50, I11.0, I13.0, I13.2
Coronary heart disease	I20-I25
Acute myocardial infarction	I21
Unstable angina	I20.0
Stroke	I60-I66, G45
Atrial fibrillation	I48
Peripheral artery disease	I70-I72, I73.1, I73.9, I74, I77.3, I77.6, I77.8, I79
Diabetes	E10-E14
Cancer	C
Head and neck cancer	C00-C14
Digestive organs cancer	C15-C26
Lung and thorax cancer	C30-C39
Skin cancer	C43-C44
Breast cancer	C50
Female genital cancer	C51-C58
Male genital cancer	C61
Urinary cancer	C64-C68
Hematologic cancer	C81-C96
**Outcomes**	
CVD	I
MACE	I50, I11.0, I13.0, I13.2, I42, I43, I25.5, I21, I60-I62, I63.0-I63.5, I63.8-I63.9, I64

CKD denotes chronic kidney disease; CVD, cardiovascular disease; MACE, major cardiovascular events. ICD-10 codes for “CKD (broad)” were used to search for diagnoses of CKD prior to the index date, which would exclude them from the analyses. ICD-10 codes for “CKD (narrow) were used to search for diagnoses of CKD during follow-up.

For peer review only - http://bmjopen.bmj.com/site/about/guidelines.xhtml

Table S5. Anatomical Therapeutic Chemical (ATC) classification system codes used to search for treatments of interest in this study's population

Treatment	ATC codes
RAASi	C09
Statin	C10AA, C10BA01-C10BA09, C10BA11-C10BA12, C10BX
SGLT-2i	A10BK01, A10BK02, A10BK03, A10BD15, A10BD16, A10BD20

RAASi denotes renin angiotensin aldosterone system inhibitors; SGLT-2i, sodium-glucose cotransporter-2 inhibitors.

For peer review only

For peer review only - http://bmjopen.bmj.com/site/about/guidelines.xhtml

Table S6. Baseline characteristics of the total study cohort stratified by age group

	18-45 years	45-65 years	65-80 years	80+ years
**N**	1,441	9,955	47,319	40,667
**Female, N (%)**	706 (49.0%)	3,830 (38.5%)	22,143 (46.8%)	24,519 (60.3%)
**Age, median (IQR)**	37.8 (30.9-41.9)	59.6 (55.0-62.7)	74.2 (70.8-77.1)	85.5 (82.6-89.1)
**Socioeconomic status, N (%)**				
Missing	776 (53.9%)	5,898 (59.2%)	30,480 (64.4%)	27,947 (68.7%)
High	90 (13.5%)	673 (16.6%)	3,743 (22.2%)	2,848 (22.4%)
Middle	216 (32.5%)	1,510 (37.2%)	7,345 (43.6%)	5,083 (40.0%)
Low	359 (54.0%)	1,874 (46.2%)	5,751 (34.2%)	4,789 (37.6%)
**Comorbidities, N (%)**				
Heart failure	45 (3.1%)	1,173 (11.8%)	7,982 (16.9%)	11,090 (27.3%)
CHD	20 (1.4%)	1,574 (15.8%)	12,268 (25.9%)	12,749 (31.3%)
AMI	9 (0.6%)	772 (7.8%)	5,463 (11.5%)	5,041 (12.4%)
Unstable angina	<=5	357 (3.6%)	2,860 (6.0%)	2,279 (5.6%)
Stroke	31 (2.2%)	828 (8.3%)	7,181 (15.2%)	8,677 (21.3%)
Atrial fibrillation	24 (1.7%)	1,038 (10.4%)	11,049 (23.4%)	13,263 (32.6%)
PAD	31 (2.2%)	510 (5.1%)	4,637 (9.8%)	3,975 (9.8%)
Diabetes	775 (53.8%)	5,031 (50.5%)	16,545 (35.0%)	9,394 (23.1%)
Cancer	58 (4.0%)	1,516 (15.2%)	14,615 (30.9%)	14,628 (36.0%)
Head and neck	6 (0.4%)	53 (0.5%)	396 (0.8%)	347 (0.9%)
Digestive	<=5	197 (2.0%)	1,739 (3.7%)	1,786 (4.4%)
Lung and thorax	<=5	112 (1.1%)	880 (1.9%)	455 (1.1%)
Skin	<=5	313 (3.1%)	5,123 (10.8%)	7,991 (19.6%)
Breast	<=5	185 (1.9%)	2,147 (4.5%)	1,836 (4.5%)
Female genital	<=5	119 (1.2%)	884 (1.9%)	778 (1.9%)
Male genital	<=5	160 (1.6%)	3,253 (6.9%)	2,397 (5.9%)
Urinary	10 (0.7%)	283 (2.8%)	1,653 (3.5%)	1,066 (2.6%)
Hematologic	13 (0.9%)	216 (2.2%)	1,215 (2.6%)	787 (1.9%)
**Measurements, median (IQR)**				
Systolic BP (mmHg)	130.0 (120.0-140.0)	137.0 (125.0-150.0)	140.0 (126.0-150.0)	140.0 (129.0-154.5)
BMI (kg/m^2)	28.8 (24.3-34.4)	29.3 (25.7-33.4)	27.1 (24.2-30.7)	24.8 (22.2-28.0)
**Laboratory, median (IQR)**				
P-creatinine (µmol/L)	71.0 (57.0-94.0)	94.0 (72.0-115.0)	97.0 (84.0-109.0)	90.0 (78.0-105.0)
eGFR (mL.min.1.73m^2)	94.2 (74.0-107.0)	59.2 (53.6-83.8)	54.6 (49.3-58.2)	50.5 (42.8-55.8)
UACR (g/mol)	9.0 (4.7-24.0)	6.4 (3.6-15.2)	3.3 (0.7-8.1)	2.5 (0.7-6.9)
Sodium (mmol/L)	139.0 (138.0-141.0)	140.0 (138.0-141.0)	140.0 (138.0-142.0)	140.0 (138.0-142.0)
Potassium (mmol/L)	4.0 (3.8-4.3)	4.2 (3.9-4.4)	4.2 (4.0-4.4)	4.2 (3.9-4.4)
HbA1c (mmol/mol)	54.0 (40.0-73.0)	50.0 (40.0-65.0)	44.0 (39.0-54.0)	42.0 (38.0-51.0)
Hemoglobin (g/L)	139.5 (126.0-152.0)	140.0 (129.0-152.0)	136.0 (126.0-146.0)	131.0 (120.0-141.0)
**Medications, N (%)**				
Statins	234 (16.2%)	4,012 (40.3%)	22,233 (47.0%)	14,253 (35.0%)

For peer review only - http://bmjopen.bmj.com/site/about/guidelines.xhtml

RAASi	452 (31.4%)	5,952 (59.8%)	29,472 (62.3%)	20,894 (51.4%)
SGLT2i	26 (1.8%)	335 (3.4%)	547 (1.2%)	78 (0.2%)

Data for baseline demographics and prevalent comorbidities were collected for each patient on the date this study identified their CKD (index date) using eGFR and UACR measurements according to the KDIGO clinical practice guidelines. The most recent clinical measurements and laboratory values within the one year prior to the index date are reported. For patients residing in Region Stockholm, the most recent socioeconomic data within the 10 years prior to the index date was collected from the Mosaic system, which applies the principles of geodemography to consumer household and individual data to categorize patients into one of the following three levels: 1) Highest income/education; 2) Medium income/education; and 3) Lowest income/education. AMI denotes acute myocardial infarction; BMI, body mass index; BP, blood pressure; CHD, coronary heart disease; eGFR, estimated glomerular filtration rate; HbA1c, glycated hemoglobin; IQR, interquartile range; PAD, peripheral artery disease; RAASi, renin-angiotensin aldosterone system inhibitors; SGLT2i, sodium-glucose cotransporter-2 inhibitors; UACR, urinary albumin-creatinine ratio.

For peer review only

Table S7. Proportions of patients diagnosed with chronic kidney disease in healthcare within five years of the development of the disease

Stage of CKD	Percentage with a diagnosis in healthcare within 5 years
S3a	29%
S3b	59%
S4+S5	82%
A2	34%
A3	62%

Each stage of chronic kidney disease (CKD) is defined using measurements of estimated glomerular filtration rate (stages S3a, S3b, and S4+S5) and urinary albumin-creatinine ratio (stages A2 and A3) according to the KDIGO clinical practice guidelines.

For peer review only

For peer review only - http://bmjopen.bmj.com/site/about/guidelines.xhtml

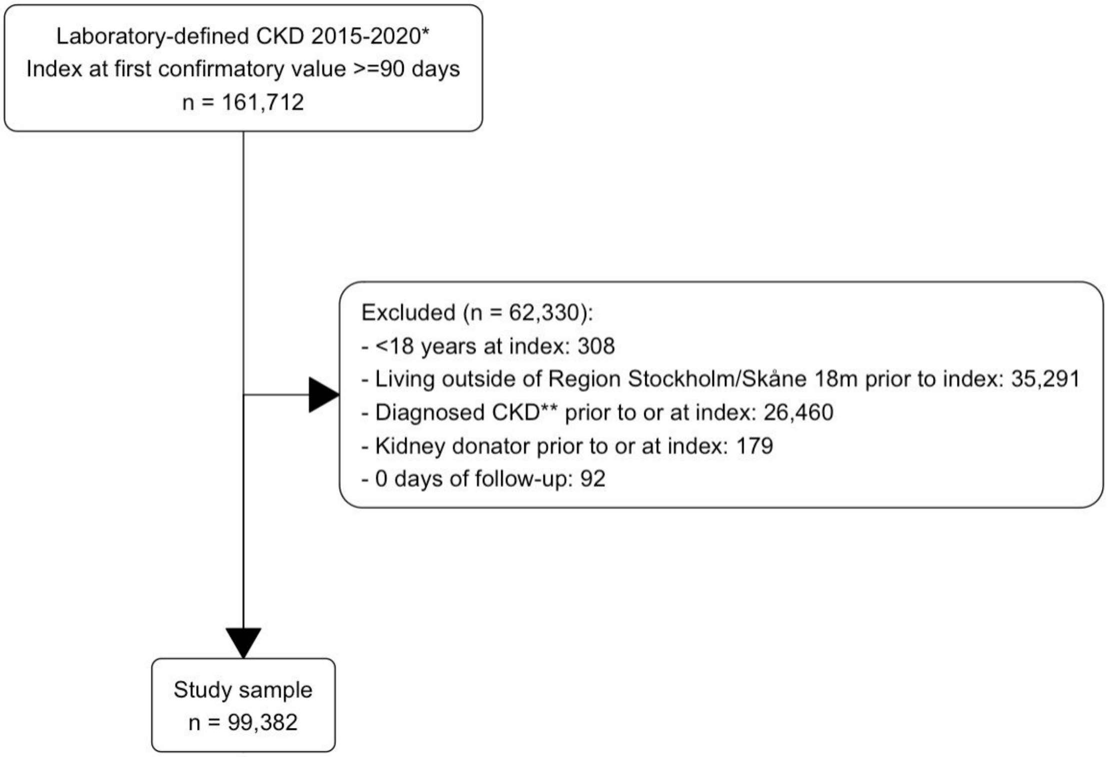

Figure S1. Participant flow

*Laboratory-defined CKD = KDIGO confirmed definition; **Diagnosed CKD = Any diagnosis position, any care level.

For peer review only - http://bmjopen.bmj.com/site/about/guidelines.xhtml

Figure S2. Multi-state models presenting the proportions of patients diagnosed or not diagnosed with chronic kidney disease (CKD) in healthcare who received or didn't receive guideline-directed pharmacological therapy, including the terminal, absorbing outcome, all-cause death

A) Statins

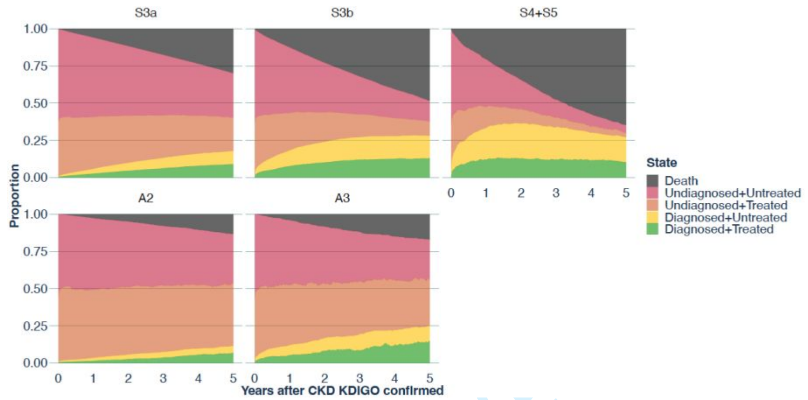

B) RAASi

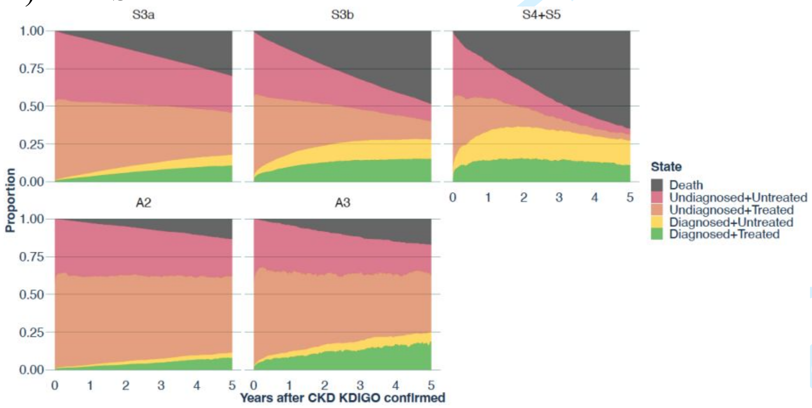

C) SGLT2i

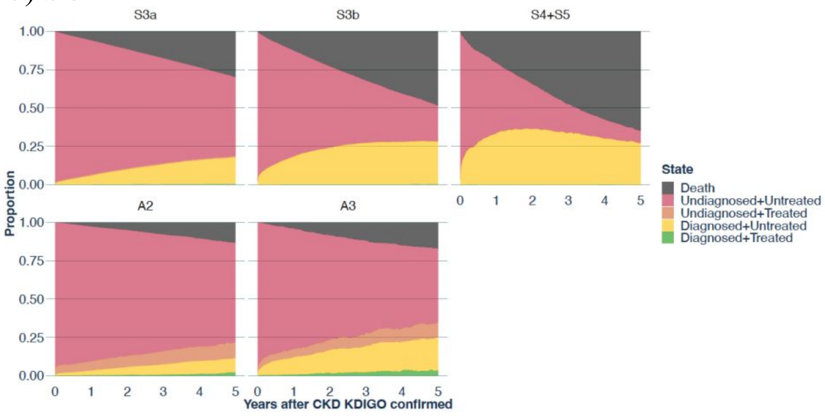

Use of A) Statins, B) renin-angiotensin aldosterone system inhibitors (RAASi), and C) sodium-glucose cotransporter-2 inhibitors (SGLT2i) were assessed. Patients are stratified by the KDIGO-defined stage of

For peer review only - http://bmjopen.bmj.com/site/about/guidelines.xhtml

their CKD, identified using estimated glomerular filtration rate (Stages S3a, S3b, and S4+S5) and urinary albumin-creatinine ratio (Stages A2 and A3).

For peer review only

For peer review only - http://bmjopen.bmj.com/site/about/guidelines.xhtml

Figure S3. Multi-state models presenting the proportions of patients diagnosed or not diagnosed with chronic kidney disease (CKD) in healthcare who received or didn’t receive guideline-directed pharmacological therapy, including the terminal, absorbing outcomes, major adverse cardiovascular events (MACE) and non-cardiovascular disease (CVD) death

A) Statins

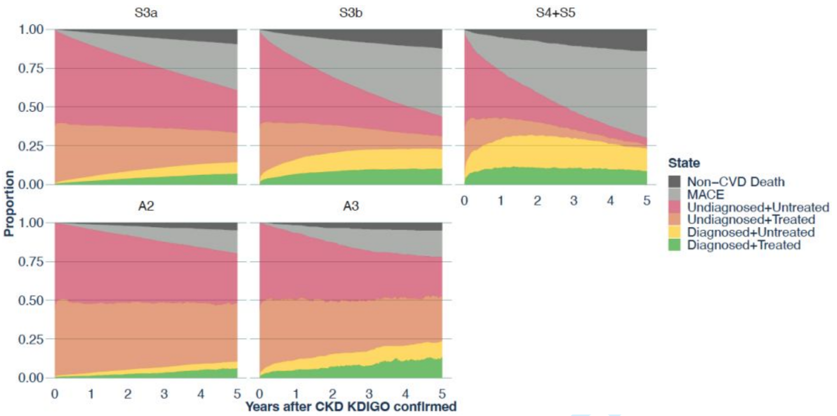

B) RAASi

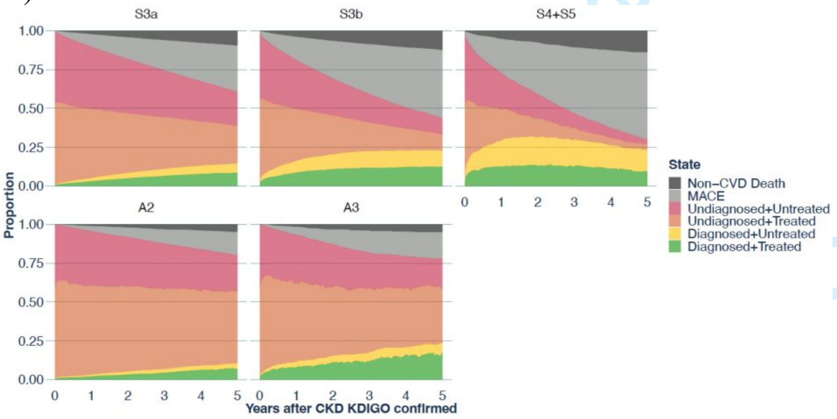

C) SGLT2i

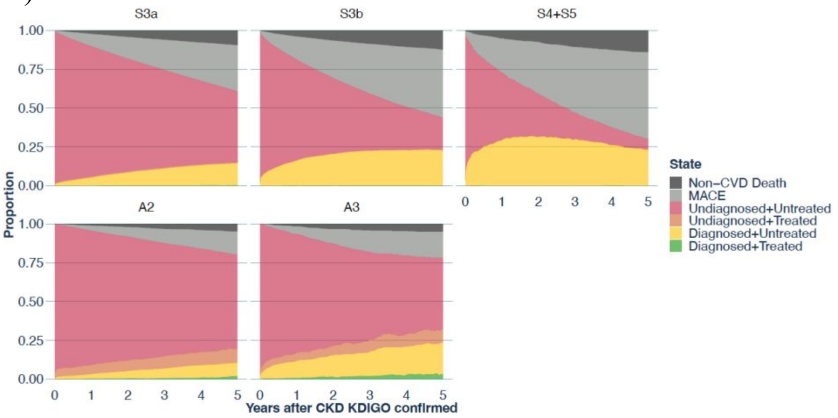

For peer review only - http://bmjopen.bmj.com/site/about/guidelines.xhtml

Use of A) Statins, B) renin-angiotensin aldosterone system inhibitors (RAASi), and C) sodium-glucose cotransporter-2 inhibitors (SGLT2i) were assessed. Patients are stratified by the KDIGO-defined stage of their CKD, identified using estimated glomerular filtration rate (Stages S3a, S3b, and S4+S5) and urinary albumin-creatinine ratio (Stages A2 and A3).

For peer review only

Figure S4. Multi-state models presenting the proportions of patients diagnosed or not diagnosed with chronic kidney disease (CKD) in healthcare who received or didn't receive guideline-directed pharmacological therapy, including the terminal, absorbing outcome, all-cause death

A) Statins

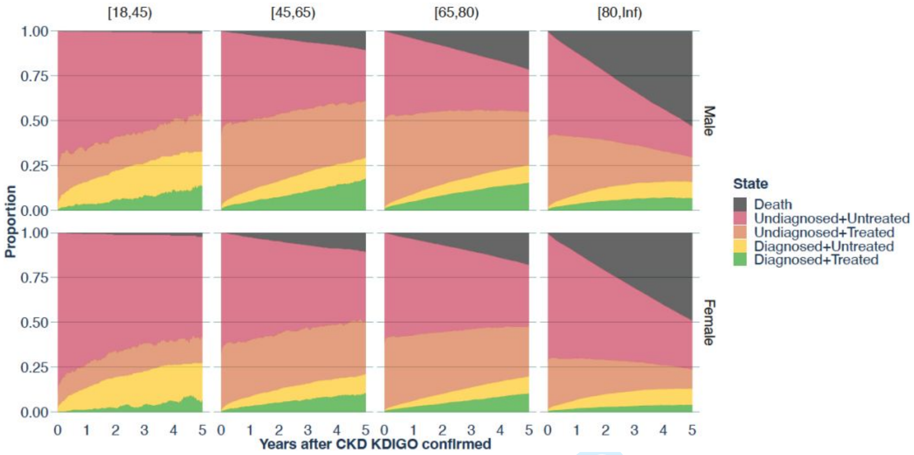

B) RAASi

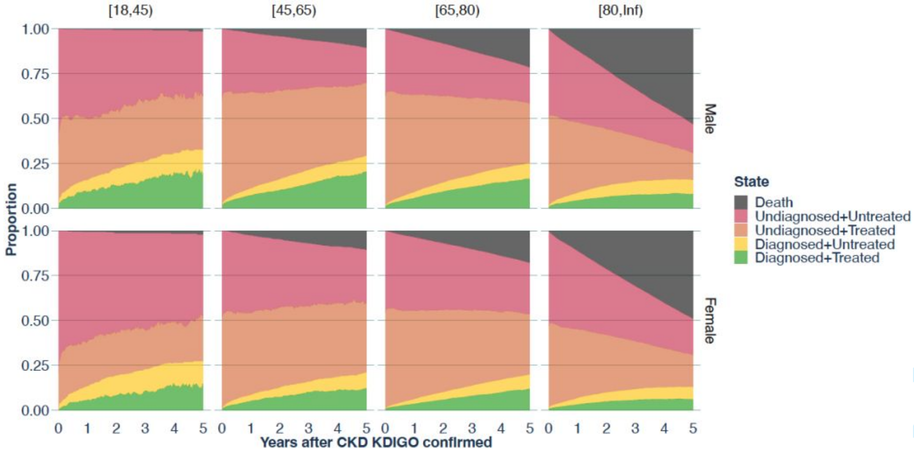

C) SGLT2i

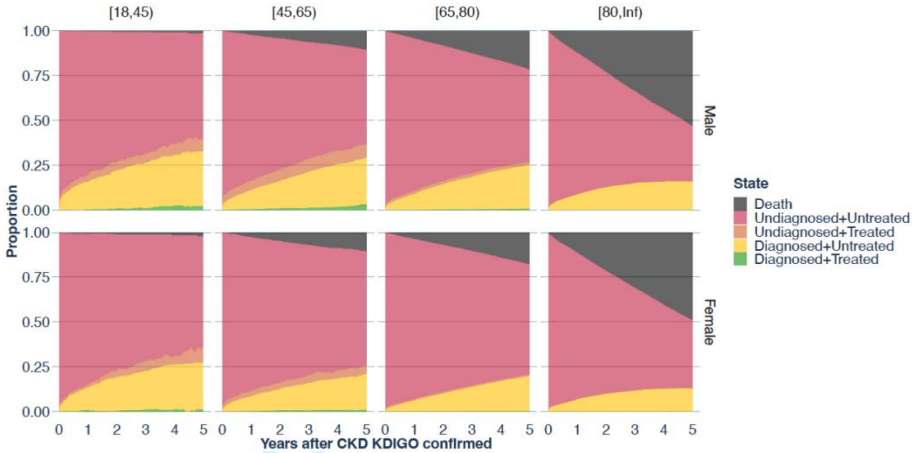

Use of A) Statins, B) renin-angiotensin aldosterone system inhibitors (RAASi), and C) sodium-glucose cotransporter-2 inhibitors (SGLT2i) were assessed. Patients are stratified by gender and age group.

For peer review only - http://bmjopen.bmj.com/site/about/guidelines.xhtml

Figure S5. Multi-state models presenting the proportions of patients diagnosed or not diagnosed with chronic kidney disease (CKD) in healthcare who received or didn't receive guideline-directed pharmacological therapy, including the terminal, absorbing outcomes, major adverse cardiovascular events (MACE) and non-cardiovascular disease (CVD) death

A) Statins

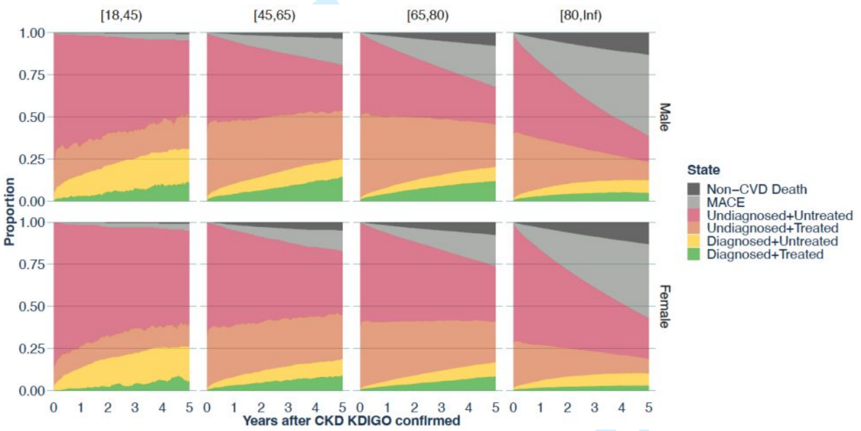

B) RAASi

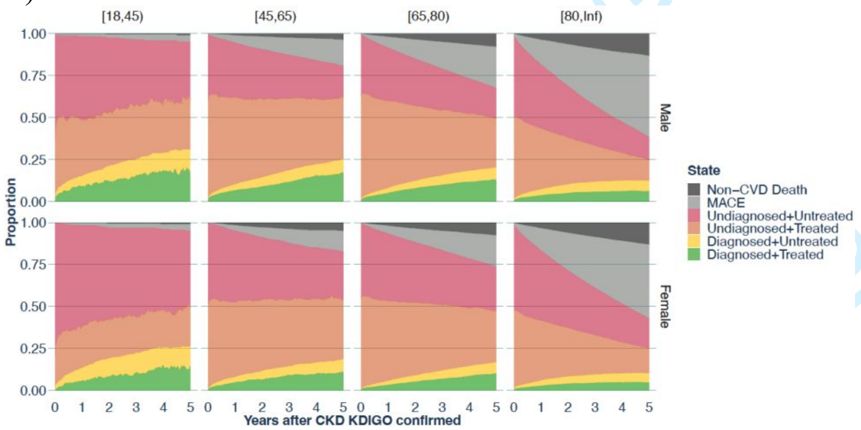

C) SLGT2i

For peer review only - http://bmjopen.bmj.com/site/about/guidelines.xhtml

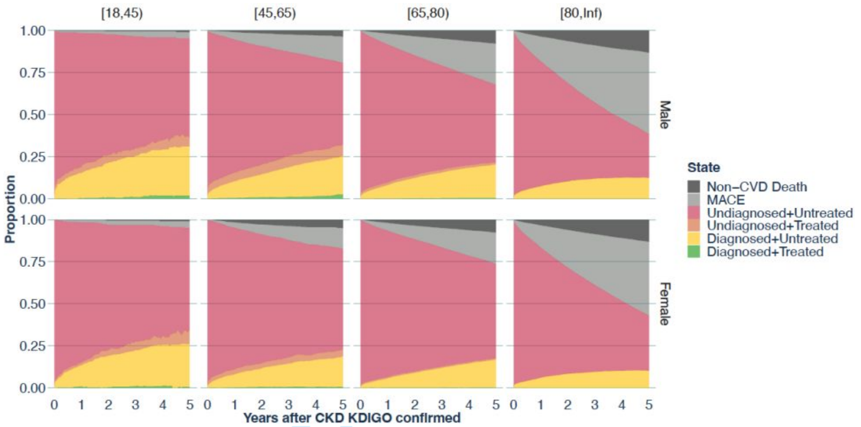

Use of A) Statins, B) renin-angiotensin aldosterone system inhibitors (RAASi), and C) sodium-glucose cotransporter-2 inhibitors (SGLT2i) were assessed. Patients are stratified by gender and age group.

For peer review only - http://bmjopen.bmj.com/site/about/guidelines.xhtml

References

1. WHO Collaborating Centre for Drug Statistics Methodology. ATC/DDD Index 2017. https://www.whocc.no/.
2. WHO. Classifications. http://www.who.int/classifications/icd/en/.
3. (NOMESCO), N. M.-S. C. NOMESCO Classification of Surgical Procedures. https://norden.diva-portal.org/smash/get/diva2:970547/FULLTEXT01.pdf (2010).
4. Ludvigsson, J. F., Otterblad-Olausson, P., Pettersson, B. U. & Ekbom, A. The Swedish personal identity number: Possibilities and pitfalls in healthcare and medical research. *European Journal of Epidemiology* **24**, 659–667 (2009).
5. Qin, Y. *et al.* The effect of angiotensin-converting enzyme inhibitor/angiotensin receptor blocker use on mortality in patients with chronic kidney disease: a meta-analysis of observational studies. *Pharmacoepidemiol Drug Saf* **25**, 503–511 (2016).
6. Balamuthusamy, S. *et al.* Renin angiotensin system blockade and cardiovascular outcomes in patients with chronic kidney disease and proteinuria: a meta-analysis. *Am Heart J* **155**, 791–805 (2008).
7. Xie, X. *et al.* Renin-Angiotensin System Inhibitors and Kidney and Cardiovascular Outcomes in Patients With CKD: A Bayesian Network Meta-analysis of Randomized Clinical Trials. *American Journal of Kidney Diseases* **67**, 728–741 (2016).
8. Zhang, Y. *et al.* ACE Inhibitor Benefit to Kidney and Cardiovascular Outcomes for Patients with Non-Dialysis Chronic Kidney Disease Stages 3–5: A Network Meta-Analysis of Randomised Clinical Trials. *Drugs* **80**, 797–811 (2020).
9. Collaboration, B. P. L. T. T. Blood pressure lowering and major cardiovascular events in people with and without chronic kidney disease: meta-analysis of randomised controlled trials. *BMJ* **347**, f5680 (2013).
10. Major, R. W., Cheung, C. K., Gray, L. J. & Brunskill, N. J. Statins and Cardiovascular Primary Prevention in CKD: A Meta-Analysis. *Clin J Am Soc Nephrol* **10**, 732–739 (2015).
11. Su, X. *et al.* Effect of Statins on Kidney Disease Outcomes: A Systematic Review and Meta-analysis. *American Journal of Kidney Diseases* **67**, 881–892 (2016).
12. Upadhyay, A. *et al.* Lipid-lowering therapy in persons with chronic kidney disease: a systematic review and meta-analysis. *Ann Intern Med* **157**, 251–262 (2012).
13. Strippoli, G. F. M. *et al.* Effects of statins in patients with chronic kidney disease: meta-analysis and meta-regression of randomised controlled trials. *BMJ* **336**, 645–651 (2008).
14. Palmer, S. C. *et al.* Benefits and harms of statin therapy for persons with chronic kidney disease: a systematic review and meta-analysis. *Ann Intern Med* **157**, 263–275 (2012).
15. Hou, W. *et al.* Effect of statin therapy on cardiovascular and renal outcomes in patients with chronic kidney disease: a systematic review and meta-analysis. *Eur Heart J* **34**, 1807–1817 (2013).
16. Zhang, X. *et al.* Effect of statins on cardiovascular events in patients with mild to moderate chronic kidney disease: a systematic review and meta-analysis of randomized clinical trials. *BMC Cardiovasc Disord* **14**, 19 (2014).
17. Barylski, M. *et al.* Statins decrease all-cause mortality only in CKD patients not requiring dialysis therapy—A meta-analysis of 11 randomized controlled trials involving 21,295 participants. *Pharmacological Research* **72**, 35–44 (2013).
18. Yamada, T. *et al.* Cardiovascular and renal outcomes with SGLT-2 inhibitors versus GLP-1 receptor agonists in patients with type 2 diabetes mellitus and chronic kidney disease: a systematic review and network meta-analysis. *Cardiovascular Diabetology* **20**, 14 (2021).

For peer review only - http://bmjopen.bmj.com/site/about/guidelines.xhtml

19. Salah, H. M. *et al.* Effect of sodium-glucose cotransporter 2 inhibitors on cardiovascular and kidney outcomes-Systematic review and meta-analysis of randomized placebo-controlled trials. *Am Heart J* **232**, 10–22 (2021).
20. Lin, D. S.-H., Lee, J.-K., Hung, C.-S. & Chen, W.-J. The efficacy and safety of novel classes of glucose-lowering drugs for cardiovascular outcomes: a network meta-analysis of randomised clinical trials. *Diabetologia* **64**, 2676–2686 (2021).
21. Kamdar, A., Sykes, R., Morrow, A., Mangion, K. & Berry, C. Cardiovascular outcomes of glucose lowering therapy in chronic kidney disease patients: a systematic review with meta-analysis. *Rev Cardiovasc Med* **22**, 1479–1490 (2021).
22. D'Andrea, E. *et al.* Heterogeneity of antidiabetic treatment effect on the risk of major adverse cardiovascular events in type 2 diabetes: a systematic review and meta-analysis. *Cardiovasc Diabetol* **19**, 154 (2020).
23. Staplin, N. *et al.* Net effects of sodium-glucose co-transporter-2 inhibition in different patient groups: a meta-analysis of large placebo-controlled randomized trials. *EClinicalMedicine* **41**, 101163 (2021).
24. Malik, A. H. *et al.* Cardiovascular Outcomes With the Use of Sodium-Glucose Cotransporter-2 Inhibitors in Patients With Type 2 Diabetes and Chronic Kidney Disease: An Updated Meta-Analysis of Randomized Controlled Trials. *Cardiol Rev* **28**, 116–124 (2020).
25. Cao, H. *et al.* Sodium-glucose cotransporter 2 inhibitors benefit to kidney and cardiovascular outcomes for patients with type 2 diabetes mellitus and chronic kidney disease 3b-4: A systematic review and meta-analysis of randomized clinical trials. *Diabetes Research and Clinical Practice* **180**, (2021).
26. Kaze, A. D., Zhuo, M., Kim, S. C., Patorno, E. & Paik, J. M. Association of SGLT2 inhibitors with cardiovascular, kidney, and safety outcomes among patients with diabetic kidney disease: a meta-analysis. *Cardiovascular Diabetology* **21**, 47 (2022).
27. Yang, S., Zhao, L., Mi, Y. & He, W. Effects of sodium-glucose cotransporter-2 inhibitors and aldosterone antagonists, in addition to renin-angiotensin system antagonists, on major adverse kidney outcomes in patients with type 2 diabetes and chronic kidney disease: A systematic review and network meta-analysis. *Diabetes, Obesity and Metabolism* **n/a**,.

For peer review only

For peer review only - http://bmjopen.bmj.com/site/about/guidelines.xhtml

STROBE 2007 (v4) Statement—Checklist of items that should be included in reports of *cohort studies*

Section/Topic	Item #	Recommendation	Reported on page #
**Title and abstract**	1	(*a*) Indicate the study's design with a commonly used term in the title or the abstract	1
		(*b*) Provide in the abstract an informative and balanced summary of what was done and what was found	2
**Introduction**			
Background/rationale	2	Explain the scientific background and rationale for the investigation being reported	4
Objectives	3	State specific objectives, including any prespecified hypotheses	4
**Methods**			
Study design	4	Present key elements of study design early in the paper	4
Setting	5	Describe the setting, locations, and relevant dates, including periods of recruitment, exposure, follow-up, and data collection	4
Participants	6	(*a*) Give the eligibility criteria, and the sources and methods of selection of participants. Describe methods of follow-up	5
		(*b*) For matched studies, give matching criteria and number of exposed and unexposed	-
Variables	7	Clearly define all outcomes, exposures, predictors, potential confounders, and effect modifiers. Give diagnostic criteria, if applicable	5-6
Data sources/ measurement	8*	For each variable of interest, give sources of data and details of methods of assessment (measurement). Describe comparability of assessment methods if there is more than one group	5-6
Bias	9	Describe any efforts to address potential sources of bias	-
Study size	10	Explain how the study size was arrived at	-
Quantitative variables	11	Explain how quantitative variables were handled in the analyses. If applicable, describe which groupings were chosen and why	6-7
Statistical methods	12	(*a*) Describe all statistical methods, including those used to control for confounding	6-7
		(*b*) Describe any methods used to examine subgroups and interactions	6-7
		(*c*) Explain how missing data were addressed	6-7
		(*d*) If applicable, explain how loss to follow-up was addressed	-
		(*e*) Describe any sensitivity analyses	-
**Results**			

For peer review only

Participants	13*	(a) Report numbers of individuals at each stage of study—eg numbers potentially eligible, examined for eligibility, confirmed eligible, included in the study, completing follow-up, and analysed	7
		(b) Give reasons for non-participation at each stage	-
		(c) Consider use of a flow diagram	Figure S1 Supplement, page 12
Descriptive data	14*	(a) Give characteristics of study participants (eg demographic, clinical, social) and information on exposures and potential confounders	8 (Table 1)
		(b) Indicate number of participants with missing data for each variable of interest	8
		(c) Summarise follow-up time (eg, average and total amount)	7
Outcome data	15*	Report numbers of outcome events or summary measures over time	10
Main results	16	(*a*) Give unadjusted estimates and, if applicable, confounder-adjusted estimates and their precision (eg, 95% confidence interval). Make clear which confounders were adjusted for and why they were included	10
		(*b*) Report category boundaries when continuous variables were categorized	10
		(*c*) If relevant, consider translating estimates of relative risk into absolute risk for a meaningful time period	10
Other analyses	17	Report other analyses done—eg analyses of subgroups and interactions, and sensitivity analyses	10
**Discussion**			
Key results	18	Summarise key results with reference to study objectives	13
**Limitations**			
Interpretation	20	Give a cautious overall interpretation of results considering objectives, limitations, multiplicity of analyses, results from similar studies, and other relevant evidence	14-15
Generalisability	21	Discuss the generalisability (external validity) of the study results	15
**Other information**			
Funding	22	Give the source of funding and the role of the funders for the present study and, if applicable, for the original study on which the present article is based	16

*Give information separately for cases and controls in case-control studies and, if applicable, for exposed and unexposed groups in cohort and cross-sectional studies.

Note: An Explanation and Elaboration article discusses each checklist item and gives methodological background and published examples of transparent reporting. The STROBE checklist is best used in conjunction with this article (freely available on the Web sites of PLoS Medicine at http://www.plosmedicine.org/, Annals of Internal Medicine at http://www.annals.org/, and Epidemiology at http://www.epidem.com/). Information on the STROBE Initiative is available at www.strobe-statement.org.

For peer review only

Are there lost opportunities in chronic kidney disease? A region-wide cohort study

Journal:	*BMJ Open*
Manuscript ID	bmjopen-2023-074064.R1
Article Type:	Original research
Date Submitted by the Author:	19-Mar-2024

Complete List of Authors:	Sundström, Johan; Uppsala University, Department of Medical Sciences; University of New South Wales, The George Institute for Global Health<br>Norhammar, Anna; Karolinska Institutet, Department of Medicine; Capio S:t Görans Hospital<br>Karayiannides, Stelios; Karolinska Institute, Department of Clinical Sciences, Danderyd Hospital ; Region Stockholm, Center for Diabetes, Academic Specialist Center<br>Bodegård, Johan; AstraZeneca PLC, Renal and Metabolism, Medical Department, BioPharmaceuticals<br>Gustafsson, Stefan; Sence Research AB<br>Cars, Thomas; Sence Research AB<br>Eriksson Svensson, Maria; Uppsala University, Department of Medical Sciences Renal Medicine<br>Ärnlöv, Johan; Karolinska Institutet, Division of Family Medicine, Department of Neurobiology, Care Sciences and Society; Dalarna University, School of Health and Social Studies

**Primary Subject Heading**:	Epidemiology
Secondary Subject Heading:	Cardiovascular medicine, Diabetes and endocrinology
Keywords:	EPIDEMIOLOGY, Chronic renal failure < NEPHROLOGY, Adult nephrology < NEPHROLOGY

I, the Submitting Author has the right to grant and does grant on behalf of all authors of the Work (as defined in the below author licence), an exclusive licence and/or a non-exclusive licence for contributions from authors who are: i) UK Crown employees; ii) where BMJ has agreed a CC-BY licence shall apply, and/or iii) in accordance with the terms applicable for US Federal Government officers or employees acting as part of their official duties; on a worldwide, perpetual, irrevocable, royalty-free basis to BMJ Publishing Group Ltd ("BMJ") its licensees and where the relevant Journal is co-owned by BMJ to the co-owners of the Journal, to publish the Work in this journal and any other BMJ products and to exploit all rights, as set out in our licence.

The Submitting Author accepts and understands that any supply made under these terms is made by BMJ to the Submitting Author unless you are acting as an employee on behalf of your employer or a postgraduate student of an affiliated institution which is paying any applicable article publishing charge ("APC") for Open Access articles. Where the Submitting Author wishes to make the Work available on an Open Access basis (and intends to pay the relevant APC), the terms of reuse of such Open Access shall be governed by a Creative Commons licence – details of these licences and which Creative Commons licence will apply to this Work are set out in our licence referred to above.

Other than as permitted in any relevant BMJ Author's Self Archiving Policies, I confirm this Work has not been accepted for publication elsewhere, is not being considered for publication elsewhere and does not duplicate material already published. I confirm all authors consent to publication of this Work and authorise the granting of this licence.

For peer review only

Are there lost opportunities in chronic kidney disease? A region-wide cohort study

Johan Sundström (1,2), Anna Norhammar (3,4), Stelios Karayiannides (5,6), Johan Bodegard (7), Stefan Gustafsson (8), Thomas Cars (8) Maria K Svensson (9,10), Johan Ärnlöv (11,12)

1) Department of Medical Sciences, Clinical Epidemiology, Uppsala University, Uppsala, Sweden
2) The George Institute for Global Health, University of New South Wales, Sydney, Australia
3) Cardiology Unit, Department of Medicine, Solna, Karolinska Institute, Stockholm, Sweden
4) Capio S:t Görans Hospital, Stockholm, Sweden
5) Department of Clinical Sciences, Karolinska Institutet, Danderyd Hospital, Stockholm, Sweden
6) Center for Diabetes, Academic Specialist Center, Region Stockholm, Sweden
7) Cardiovascular, Renal and Metabolism, Medical Department, BioPharmaceuticals, AstraZeneca, Oslo, Norway
8) Sence Research AB, Uppsala, Sweden
9) Department of Medical Sciences, Renal Medicine, Uppsala University, Uppsala, Sweden
10) Uppsala Clinical Research Center, Uppsala, Sweden
11) Division of Family Medicine, Department of Neurobiology, Care Sciences and Society, Karolinska Institutet, Huddinge, Sweden
12) School of Health and Social Studies, Dalarna University, Falun, Sweden

Word count: 4120

Correspondence to:
Professor Johan Sundström, MD PhD
Department of Medical Sciences, Uppsala University
Entrance 40, 5th floor; Uppsala University Hospital; 75185 Uppsala, Sweden
johan.sundstrom@uu.se
+46704225220

For peer review only - http://bmjopen.bmj.com/site/about/guidelines.xhtml

ABSTRACT

Objectives

Identify the windows of opportunity for diagnosis of chronic kidney disease (CKD) and the prevention of its adverse outcomes, and to quantify the potential population gains of such prevention.

Design and setting

Observational, population-wide study of residents in the Stockholm and Skåne regions of Sweden between January 1st, 2015, and December 31st, 2020.

Participants

All patients who didn't yet have a diagnosis of CKD in healthcare, but had CKD according to laboratory measurements of CKD biomarkers available in electronic health records.

Outcome measures

We assessed the proportions of the patient population that received a subsequent diagnosis of CKD in healthcare, that used guideline-directed pharmacological therapy (statins, renin-angiotensin aldosterone system inhibitors [RAASi], and/or sodium-glucose cotransporter-2 inhibitors [SGLT2i]), and that experienced adverse outcomes (all-cause mortality, cardiovascular mortality, or major adverse cardiovascular events [MACE]). The potential to prevent adverse outcomes in CKD was assessed using simulations of guideline-directed pharmacological therapy in untreated subsets of the study population.

Results

We identified 99,382 patients with undiagnosed CKD during the study period. Only 33% of those received a subsequent diagnosis of CKD in healthcare after five years. The proportion that used statins or RAASi was of similar size to the proportion that didn't, regardless of how advanced their CKD was. Use of SGLT2i was negligible. In simulations of optimal treatment, 22% of the 21,870 deaths, 27% of the 14,310 cardiovascular deaths, and 39% of the 22,224 MACE could have been avoided if every patient that didn't use an indicated medication for their laboratory-confirmed CKD was treated with guideline-directed pharmacological therapy for CKD.

Conclusions

While we noted under-diagnosis and under-treatment of CKD in this large contemporary population, we also identified a substantial realizable potential to improve CKD outcomes and reduce its burden by treating patients early with guideline-directed pharmacological therapy.

For peer review only - http://bmjopen.bmj.com/site/about/guidelines.xhtml

STRENGTHS AND LIMITATIONS OF THIS STUDY

- This study was strengthened by its access to health registries and electronic health records detailing most incidences of healthcare and medication use by residents in two of Sweden's largest regions, representing over a third of the national population.

- Given that only persons with suspected or known other disease have provided the samples necessary to make a laboratory-based assessment of CKD status, it is likely that this study underestimates the number of patients with undiagnosed CKD.

- This study couldn't assess if patients from a specific socioeconomic background or ethnicity were disproportionally affected by the deficiencies in CKD-related healthcare.

- Simulations of guideline-directed pharmacological therapy assumed that treatment was initiated at the time CKD was identified using laboratory measurements and that the adherence to treatment would be similar to that obtained in clinical trials, efficiency and success in treatment that is unlikely in routine clinical practice.

For peer review only

For peer review only - http://bmjopen.bmj.com/site/about/guidelines.xhtml

INTRODUCTION

Current estimates indicate that one in ten adults has CKD (between 700 million and one billion people globally).(1–3) As the prevalence of CKD increases with an aging population and growing rates of CKD risk factors (e.g., diabetes and hypertension),(4) so too does its contribution to total mortality and its economic burden;(2,5,6) highlighting the need to evaluate CKD diagnosis and treatment patterns.

Given that cardiovascular disease is a major cause of premature death among patients with CKD, management of cardiovascular disease risk factors (e.g., high cholesterol, hypertension, and chronic hyperglycemia) remains central to the treatment of CKD. Statins, renin-angiotensin aldosterone system inhibitors (RAASi), and sodium-glucose cotransporter-2 inhibitors (SGLT2i) are first-line pharmacological therapies in patients with CKD;(7–9) each demonstrated to reduce the risk of major adverse cardiovascular events (MACE) in this patient population (Table S1). Data from recent large cohort studies suggest that low proportions of patients with any degree of laboratory-confirmed CKD are diagnosed with CKD or treated with these drugs.(1,10,11) While low use of SGLT2i may be explained by the recency of their inclusion in CKD treatment guidelines,(11–13) it is less clear as to why statins and RAASi have been underused, considering that they have been readily available in clinical practice for more than 20 years. Hence, detailed characterization of prevalence and consequences of sub-optimal CKD-related prevention and healthcare, and determination of the potential for improving outcomes in patients with CKD, are of great public health importance.

For that purpose, we identified a large cohort of patients with CKD using laboratory data in electronic health records, with the aim to detail the proportions of those patients that received a subsequent diagnosis of CKD in healthcare, that used guideline-directed pharmacological therapy for their CKD, and that developed adverse outcomes. We also aimed to identify windows of opportunity for preventing adverse outcomes in patients with CKD, by assessing the absolute numbers of adverse outcomes that would be prevented by the simulated application of several variants of guideline-directed pharmacological therapy.

METHODS

Data sources

Sweden has a comprehensive, nationwide public healthcare system to which each resident has access with a minor co-payment for healthcare visits, hospitalizations and medications.(14) Residents have a unique personal identification number (person-ID),(15) which is mandatory for all administrative purposes, including any contact with the healthcare system and filling of drug prescriptions. Thus, providing a basis for complete population-wide medical history.

The present study utilized data from the CELOSIA database, the details of which can be viewed in the Supplementary File. Briefly, it includes data from the Swedish Prescribed Drug Register, the Cause of Death Register, the National Patient Register, and from several data sources with regional coverage including electronic health records in the Stockholm

For peer review only - http://bmjopen.bmj.com/site/about/guidelines.xhtml

and Skåne regions. A complete list of the Anatomical Therapeutic Chemical (ATC) codes,(16) International Classification of Diseases (ICD)-10 codes,(17) procedure codes,(18) and laboratory values used in the CELOSIA database is available in Table S2.

Individual patient-level data from the national registers and regional data sources were linked using the person-ID. The linked pseudonymized database was managed separately by Sence Research AB, Uppsala, Sweden. The study was approved by the Swedish Ethical Review Authority (approval numbers 2020-03850 and 2020-06716).

Study population

Participant flow through the study is described in Figure S1. Laboratory measurements of creatinine-based estimated glomerular filtration rate (eGFR) and/or urine albumin-creatinine ratio (UACR), available in the electronic health records, were used to identify patients >18 years of age with CKD (irrespectively of whether it was diagnosed, indicated by the presence of an ICD code in healthcare databases) according to the Kidney Disease: Improving Global Outcomes (KDIGO) clinical practice guidelines.(8) Meaning, CKD was identified if a patient presented at least two abnormal eGFR measurements (<60 ml/min/1.73m^2) and/or two abnormal UACR measurements (≥3 g/mol), with the first and last measurements taken at least 90 days apart and where all measurements in between were abnormal.

The last abnormal measurement, which would have allowed the identification of CKD, needed to have been taken between January 1st, 2015, and December 31st, 2020. The first abnormal measurement, however, could have been taken prior to the start of that study period. The date at which CKD was identified was considered the index date. The revised Lund-Malmö equation was used to calculate eGFR from creatinine, age and sex;(19) and UACR was expressed in g/mol. The eGFR and UACR thresholds defining each stage of CKD according to KDIGO guidelines are detailed in Table S3.

At the time of and within the 18 months prior to the date that CKD was identified (index), patients needed to be residing in Region Stockholm or Region Skåne where electronic health records were available. Patients with a prior diagnosis of CKD in healthcare or who had donated a kidney were excluded (ICD-10 codes used to identify those patients are listed in Table S4; ICD-10 codes for a broad CKD definition were used). Patients with zero days of follow-up (i.e., those who died at the time CKD was identified) were also excluded (n=92).

Follow-up and characterization of exposures, outcomes, and comorbidities

Patients were followed for up to five years or until the censor date (December 31st, 2020), whichever occurred first. Adverse outcomes of interest during follow-up were all-cause death, non-cardiovascular death, and MACE, where MACE was defined as a primary diagnosis of MACE in inpatient care or a primary/non-primary diagnosis of death caused by cardiovascular disease.

To evaluate the lapse in time between the laboratory-based identification of CKD and a subsequent diagnosis of CKD in healthcare, ICD-10 codes indicating a CKD diagnosis in healthcare were also searched for. Prevalent comorbidities were defined as all diagnoses up until the laboratory-based identification of CKD. Diagnoses available from primary care,

For peer review only - http://bmjopen.bmj.com/site/about/guidelines.xhtml

specialized outpatient care and inpatient care were used, including both primary and non-primary diagnoses. The ICD-10 codes used to identify diagnoses of CKD in healthcare (codes for a narrow CKD definition were used), prevalent comorbidities, and the adverse outcomes of interest are listed in Table S4.

To characterize the use of guideline-directed pharmacological therapy within the total study population, filled prescriptions for statins, RAASi, and SGLT2i were searched for using the ATC codes listed in Table S5. For each medication, a patient was considered treated throughout the follow-up period if a new prescription was filled before the coverage period of the previous filled prescription expired. The coverage period for each filled prescription was calculated as the total number of pills dispensed divided by the prescribed daily dosage. This coverage period was extended by a 25% grace period, allowing for small deviations (e.g., potentially due to imperfect medication adherence) from the expected dispensation pattern.

Statistical analyses

All statistical analyses were performed using R version 3.6.0, and the R package Epi was used for all multi-state analyses.(20,21)

Proportions of patients with diagnoses, treatments, and adverse outcomes

To detail the proportion of patients with laboratory-identified CKD that received a subsequent diagnosis of CKD in healthcare and/or used a guideline-directed pharmacological therapy (statins, RAASi, and/or SGLT2i), as well as the proportion affected by adverse outcomes, multi-state models were constructed with four possible transient states:

1) Undiagnosed and untreated.
2) Undiagnosed and treated.
3) Diagnosed and untreated.
4) Diagnosed and treated.

The transient nature of the multi-state models meant that any change in a patient's state during follow-up was accounted for by the model and reflected in the results (e.g., any change in treatment status over time or at the first diagnosis of CKD in healthcare). Each model also included either all-cause death, non-cardiovascular disease death, or MACE as terminal, absorbing states. The Aalen-Johansen estimator of transition probabilities was used in a multi-state model adjusting for age (categorical [18,45) [45,65) [65,80) [80,Inf)) and sex.

In figures for the multi-state models and all tables where patients are stratified by the severity of their CKD, patients were stratified primarily using eGFR measurements. If eGFR data were normal or unavailable, UACR measurements were used to stratify the patients. To detail the lapse in time between the laboratory-based identification of CKD and a diagnosis of CKD in healthcare, a treatment, and/or an adverse event, the Aalen-Johansen estimator was calculated along the time scale, years since the laboratory-based identification of CKD. Kaplan-Meier curves were used to present the proportions of patients who received a diagnosis of CKD in healthcare after the laboratory-based identification of CKD.

For peer review only - http://bmjopen.bmj.com/site/about/guidelines.xhtml

Simulations of population effects of guideline-directed pharmacological therapy

To evaluate the potential to prevent adverse outcomes in patients with CKD, several variants of guideline-directed pharmacological therapy were simulated in non-overlapping subsets of the study population, which included all patients who weren't treated with one or more of statins, RAASi or SGLT2i throughout follow-up. For example, in simulations of statin therapy, patients included in the analysis couldn't have been treated with statins during the entire follow-up period, but they could have been treated with RAASi or SGLT2i. Separate analyses were conducted for monotherapy with each treatment and for all possible combinations of treatment in polytherapy (e.g., any two of the medications of interest or all three medications in combination).

Hazard ratios from several large systematic reviews and meta-analyses,(22–24) which provided a comprehensive compilation of randomized controlled trials that evaluated the effect of statin, RAASi, or SGLT2i treatment on all-cause death, cardiovascular death, and MACE in patients with CKD, were used to calculate the probability of five-year survival for those outcomes, simulating the use of guideline-directed pharmacological therapy in the total number of patients untreated with that specific therapy. From the difference between the actual probability of an event in the untreated population, as observed during follow-up, and the estimated probability of five-year survival, $S_{untreated}(5y)$ and $S_{treated}(5y)$, the absolute reduction in risk of an adverse event, Δ_{risk}, was calculated. Using this value, the number needed to treat, NNT, to prevent one adverse event, and the number of prevented deaths (by any cause) and MACE were estimated for each treatment.

$$S_{treated}(5y) = S_{untreated}(5y)^{\exp\left(log_n(HR_{RAASi}) + log_n(HR_{statin}) + log_n(HR_{SGLT2i})\right)};$$
$$\Delta_{risk} = \left(1 - S_{untreated}(5y)\right) - \left(1 - S_{treated}(5y)\right);$$
$$NNT = {}^{1}/\Delta_{risk};$$
$$N_{saved} = {}^{N_{untreated}}/NNT$$

It was assumed that the treatment effect reported by each meta-analysis was replicable in each simulation and that medication adherence was similar to that obtained in clinical trials from the date that CKD was identified using laboratory measurements. For simulations of monotherapy, the relevant hazard ratio extracted from each meta-analysis was used. For combination therapy, hazard ratios were calculated using the hazard ratios extracted from the three meta-analyses under the assumption that there was no deviation from a multiplicative relative effect of using treatments in combination (i.e., no interaction between medications), as shown previously.(25–27) Multiple sensitivity analyses were conducted to account for variable treatment effects due to differences in the population, including a subgroup of younger age (with higher likelihood to be considered for preventive treatment); a subgroup with type-2 diabetes or hypertension (which would already have other indications for some of the drugs and hence be more likely to be considered for preventive treatment); and a subgroup that survived more than 90 days after index (which would be more likely to be considered for preventive treatment than very sick patients); and variable treatment effects due to differences in the treatment, including combinations of assumptions of multiplicative effects of all drugs assumed, but HR for SGLT2i set to 1; and assumptions of no multiplicative effects, only the HR from the

For peer review only - http://bmjopen.bmj.com/site/about/guidelines.xhtml

drug with the largest effect counted, HR for all other drugs set to 1. These analyses also investigated the influence of less-than-optimal treatment effect (the intention-to-treat effect obtained in clinical trials); i.e. if there are delays in initiating treatment, and/or adherence is less than that obtained in clinical trials, and/or other reasons for not expecting optimal treatment effect, a number less than 100 could be considered.

Patient and public involvement

Patients and the public were not involved in the design, conduct, reporting, or dissemination plans of this study.

RESULTS

Baseline characteristics

In a background population of ~3.8 million residents between the beginning of 2015 and the end of 2020, a total of 99,382 patients without a medical record of CKD were identified as having CKD using laboratory measurements of eGFR and/or UACR. The characteristics of these patients are presented in Table 1 and Table S6. Overall, patients were elderly (median age, 77.7 years; IQR, 71.3-84.2 years), and the prevalence of cardiovascular diseases and diabetes were high. At baseline, those with CKD stages A2 and A3 were younger, had higher BMI, were more often male, more often had diabetes, and more often had preventive drug treatment, than those with stages S3a to S5 (Table 1). Patients were followed for a median of 3.0 years (IQR, 1.6-4.5 years).

For peer review only

For peer review only - http://bmjopen.bmj.com/site/about/guidelines.xhtml

Table 1. Baseline characteristics of the total study cohort with laboratory-identified chronic kidney disease and that stratified by KDIGO-defined stages of chronic kidney disease

	Total	S3a eGFR 45-59 mL/min/1.73m²	S3b eGFR 30-44 mL/min/1.73m²	S4+S5 eGFR < 30 mL/min/1.73m²	A2 UACR 3-30 g/mol	A3 UACR > 30 g/mol
**N**	99,382	72,407	14,315	1,740	9,549	1,371
**Female, N (%)**	51,198 (51.5%)	37,477 (51.8%)	8,465 (59.1%)	1,180 (67.8%)	3,532 (37.0%)	544 (39.7%)
**Age, median (IQR)**	77.7 (71.3-84.2)	77.9 (72.3-83.6)	83.4 (76.7-88.8)	87.3 (81.5-91.6)	65.3 (56.0-73.2)	62.0 (50.5-70.4)
**Socioeconomic status, N (%)**						
Missing	65,101 (65.5%)	46,897 (64.8%)	10,187 (71.2%)	1,342 (77.1%)	5,757 (60.3%)	918 (67.0%)
High	7,354 (21.5%)	5,847 (22.9%)	881 (21.3%)	85 (21.4%)	497 (13.1%)	44 (9.7%)
Middle	14,154 (41.3%)	10,752 (42.1%)	1,656 (40.1%)	135 (33.9%)	1,444 (38.1%)	167 (36.9%)
Low	12,773 (37.3%)	8,911 (34.9%)	1,591 (38.5%)	178 (44.7%)	1,851 (48.8%)	242 (53.4%)
**Comorbidities, N (%)**						
Heart failure	20,290 (20.4%)	14,511 (20.0%)	4,274 (29.9%)	625 (35.9%)	744 (7.8%)	136 (9.9%)
CHD	26,611 (26.8%)	19,555 (27.0%)	4,543 (31.7%)	555 (31.9%)	1,701 (17.8%)	257 (18.7%)
AMI	11,285 (11.4%)	8,197 (11.3%)	1,999 (14.0%)	255 (14.7%)	725 (7.6%)	109 (8.0%)
Unstable angina	5,498 (5.5%)	4,075 (5.6%)	829 (5.8%)	114 (6.6%)	424 (4.4%)	56 (4.1%)
Stroke	16,717 (16.8%)	12,256 (16.9%)	2,982 (20.8%)	370 (21.3%)	974 (10.2%)	135 (9.8%)
Atrial fibrillation	25,374 (25.5%)	19,035 (26.3%)	4,515 (31.5%)	575 (33.0%)	1,099 (11.5%)	150 (10.9%)
PAD	9,153 (9.2%)	6,649 (9.2%)	1,543 (10.8%)	184 (10.6%)	676 (7.1%)	101 (7.4%)
Diabetes	31,745 (31.9%)	19,510 (26.9%)	4,019 (28.1%)	441 (25.3%)	6,866 (71.9%)	909 (66.3%)
Cancer	30,817 (31.0%)	23,735 (32.8%)	4,652 (32.5%)	571 (32.8%)	1,664 (17.4%)	195 (14.2%)
**Measurements, median (IQR)**						
Systolic BP (mmHg)	140.0 (127.0-150.0)	140.0 (126.0-150.0)	140.0 (125.0-153.0)	140.0 (125.0-155.0)	140.0 (130.0-150.0)	140.0 (130.0-153.0)
BMI (kg/m²)	26.5 (23.5-30.1)	26.2 (23.4-29.7)	25.8 (22.8-29.5)	25.6 (22.6-29.3)	29.0 (25.4-33.1)	29.4 (25.4-33.7)
**Laboratory, median (IQR)**						
P-creatinine (µmol/L)	94.0 (81.0-108.0)	93.0 (82.0-105.0)	116.0 (102.0-133.0)	151.0 (133.0-178.0)	69.0 (59.0-80.0)	70.0 (58.2-83.0)
eGFR (mL/min/1.73m²)	53.7 (46.9-57.9)	54.1 (49.8-57.3)	38.9 (33.8-43.0)	24.5 (20.9-28.3)	78.9 (70.2-90.1)	80.0 (70.6-92.8)
UACR (g/mol)	4.0 (1.0-9.7)	1.3 (0.5-4.6)	2.5 (0.7-9.6)	6.6 (2.1-27.6)	6.6 (4.3-12.0)	54.1 (34.0-90.0)
Sodium (mmol/L)	140.0 (138.0-142.0)	140.0 (138.0-142.0)	140.0 (138.0-142.0)	140.0 (138.0-142.0)	140.0 (138.0-141.0)	139.0 (137.0-141.0)
Potassium (mmol/L)	4.2 (3.9-4.4)	4.2 (3.9-4.4)	4.2 (4.0-4.5)	4.3 (4.0-4.7)	4.1 (3.9-4.3)	4.1 (3.9-4.3)
HbA1c (mmol/mol)	44.0 (39.0-55.0)	42.0 (38.0-51.0)	44.0 (38.0-55.0)	44.0 (38.0-57.0)	53.0 (44.0-67.0)	53.0 (42.0-69.0)
Hemoglobin (g/L)	134.0 (123.0-145.0)	135.0 (124.0-145.0)	128.0 (117.0-139.0)	122.0 (111.0-132.0)	142.0 (131.0-152.0)	140.0 (128.0-153.0)

For peer review only - http://bmjopen.bmj.com/site/about/guidelines.xhtml

**Medications, N (%)**						
Statins	40,732 (41.0%)	29,271 (40.4%)	5,594 (39.1%)	625 (35.9%)	4,611 (48.3%)	631 (46.0%)
RAASi	56,770 (57.1%)	40,626 (56.1%)	8,460 (59.1%)	972 (55.9%)	5,854 (61.3%)	858 (62.6%)
SGLT2i	986 (1.0%)	470 (0.6%)	52 (0.4%)	≤5	412 (4.3%)	49 (3.6%)

Data for baseline demographics and prevalent comorbidities were collected for each patient on the date this study identified their chronic kidney disease (CKD; index date) using laboratory measurements of eGFR and UACR according to the KDIGO clinical practice guidelines. The most recent clinical measurements and laboratory values within the one year prior to the index date are reported. For patients residing in Region Stockholm, the most recent socioeconomic data within the 10 years prior to the index date was collected from the Mosaic system, which applies the principles of geodemography to consumer household and individual data to categorize patients into one of the following three levels: 1) Highest income/education; 2) Medium income/education; and 3) Lowest income/education. Stratification of the patients into the different KDIGO-defined stages of CKD using eGFR (stages S3a, S3b, and S4+S5) and UACR measurements (stages A2 and A3) is based on the median of all measurements in the ≥90-day period required to diagnose CKD. AMI denotes acute myocardial infarction; BMI, body mass index; BP, blood pressure; CHD, coronary heart disease; eGFR, estimated glomerular filtration rate; HbA1c, glycated hemoglobin; IQR, interquartile range; PAD, peripheral artery disease; RAASi, renin-angiotensin aldosterone system inhibitors; SGLT2i, sodium-glucose cotransporter-2 inhibitors; UACR, urinary albumin-creatinine ratio.

For peer review only

Two out of three with chronic kidney disease remain undiagnosed after five years

After five years from the date that CKD was identified using laboratory measurements, only 33% of the 99,382 patients received a subsequent diagnosis of CKD in healthcare. Of that 33%, the median time to a diagnosis was 1.4 years (IQR, 0.4-2.7 years). The proportion of patients that received a subsequent diagnosis of CKD in healthcare varied between stages of KDIGO-defined CKD, largely irrespective of age (Figure 1 and Figure 2). Notably, the rates over time of receiving a CKD diagnosis was similar in stages S3a and A2, and stages S3b and A3, respectively (Figure 1 and Figure 2).

In patients with less advanced CKD (KDIGO-defined CKD stage 3a), identified using eGFR measurements, the proportion of patients that received a diagnosis of CKD in healthcare was lower than in those with more advanced CKD (KDIGO stages 4 and 5 CKD) (29% *versus* 82%). A similar pattern was found in those with CKD identified using UACR measurements (KDIGO stage A2 CKD, 34% *versus* KDIGO stage A3 CKD, 62%; Table S7). Analogously, the time to a diagnosis of CKD in healthcare was shorter in patients with more advanced stages of CKD.

Use of guideline-directed pharmacological therapy in relation to a chronic kidney disease diagnosis

The proportions of patients, stratified by the severity of their CKD according to eGFR or UACR measurements, who received a CKD diagnosis in healthcare or who used a guideline-directed pharmacological therapy are presented in Figure 3. Interestingly, around half of the patients were already using statins or RAASi when CKD was identified using laboratory measurements. Use of SGLT2i was negligible, as anticipated.

Use of combination guideline-directed pharmacological therapy might greatly reduce absolute adverse outcome rates

After five years, 21,870 persons died (of any cause), 14,310 persons died from cardiovascular disease, and 22,442 persons experienced a MACE. The proportion of patients that died from any cause or had a MACE increased with each worsening stage of CKD (Figure S2 and Figure S3). Similary, proportions of those adverse outcomes increased with each incremental increase in age (Figure S4 and Figure S5). Findings were similar between men and women.

In simulated monotherapy of statins, RAASi or SGLT2i reduced the incidences of all-cause death, cardiovascular death, and MACE by 6-23% (Table 2). When variations of dual therapy were simulated, the incidences of all-cause death, cardiovascular death, and MACE were reduced by 14-35%. The greatest reductions in the incidences of all-cause death, cardiovascular death, and MACE were estimated when all three pharmacological treatments were used in combination, preventing 25% of all-cause deaths, 26% of cardiovascular deaths, and 46% of MACE. If every patient that didn't use an indicated medication for their laboratory-confirmed CKD was treated according to CKD treatment guidelines, 5,014 (22%) of the 21,870 all-cause deaths, 3,871 (27%) of the 14,310 cardiovascular deaths, and 8,722 (39%) of the 22,442 MACE could have potentially been

For peer review only - http://bmjopen.bmj.com/site/about/guidelines.xhtml

avoided. If everyone that wasn't treated with triple therapy had received it, the number needed to treat to avoid one all-cause death, one cardiovascular death, and one MACE would be 11, 15 and 7, respectively.

The sensitivity analysis showed that the potential to prevent all-cause deaths, cardiovascular deaths, and MACE decreased as the proportion that received optimal treatment decreased (Figure 4). The potential to prevent these events was most notably lower in younger patients and when treatment with SGLT2i was not included as part of combination therapy.

For peer review only

For peer review only - http://bmjopen.bmj.com/site/about/guidelines.xhtml

Table 2. Number of adverse events potentially prevented over five years in simulated provision of monotherapy and combination pharmacological therapy, in optimal settings, to all that were untreated with those corresponding regimens

Treatment	Number of patients without specific treatment regimen, additional possibility to treat	Outcome (Number of events)	Probability of an event in untreated	HR from meta-analyses	Estimated probability of an event	Absolute difference in risk	Number needed to treat	Events prevented
**Eligible for additional monotherapy with:**								
Statins	402	Death (23)	0.084	0.92 (0.85-0.99)	0.077	0.007	156	3
RAASi	353	Death (11)	0.074	0.94 (0.84-1.07)	0.070	0.004	234	2
SGLT2i	38760	Death (6415)	0.250	0.87 (0.80-0.95)	0.221	0.029	35	1106
Statins	402	CVD death (18)	0.065	0.91 (0.84-0.99)	0.060	0.005	175	2
RAASi	353	CVD death (4)	0.018	0.94 (0.78-1.04)	0.017	0.001	935	0
SGLT2i	38760	CVD death (4603)	0.188	0.87 (0.79-0.95)	0.165	0.023	45	862
Statins	400	MACE (47)	0.199	0.77 (0.70-0.85)	0.157	0.042	24	17
RAASi	345	MACE (14)	0.083	0.84 (0.78-0.91)	0.070	0.013	78	4
SGLT2i	36766	MACE (7477)	0.294	0.84 (0.75-0.94)	0.254	0.040	25	1488
**Eligible for additional dual therapy with:**								
Statins + RAASi	142	Death (12)	0.271	0.86	0.239	0.032	31	5
Statins + SGLT2i	25877	Death (6252)	0.359	0.80	0.299	0.059	17	1539
RAASi + SGLT2i	10560	Death (2363)	0.327	0.82	0.277	0.050	20	532
Statins + RAASi	142	CVD death (5)	0.100	0.86	0.086	0.014	72	2
Statins + SGLT2i	25877	CVD death (4331)	0.268	0.79	0.219	0.049	20	1273
RAASi + SGLT2i	10560	CVD death (1539)	0.228	0.82	0.191	0.037	27	393
Statins + RAASi	146	MACE (10)	0.181	0.65	0.121	0.060	17	9
Statins + SGLT2i	26738	MACE (6728)	0.368	0.65	0.257	0.111	9	2974
RAASi + SGLT2i	10621	MACE (2344)	0.325	0.71	0.243	0.082	12	881
**Eligible for triple therapy**								
All therapies	20420	Death (6661)	0.464	0.75	0.374	0.090	11	1827

For peer review only - http://bmjopen.bmj.com/site/about/guidelines.xhtml

All therapies	20420	CVD death (3709)	0.293	0.74	0.228	0.065	15	1339
All therapies	21836	MACE (5604)	0.387	0.54	0.233	0.154	7	3349

The outcome death is death by any cause. Treatment will all therapies indicates simulated treatment with a combination of statins, renin-angiotensin aldosterone inhibitors, and sodium-glucose cotransporter-2 inhibitors. The probability of an event in untreated is the probability of an event observed in the given sub-population during follow-up. The estimated probability of event is the probability of an event when each given treatment is simulated using the hazard ratio for monotherapy reported in the relevant meta-analysis, or the hazard ratio calculated under the assumption of a multiplicative relative effect of using treatments in combination. The absolute difference in risk is the difference in the observed probability of an event and the estimated probability of an event. The number needed to treat is the number of patients that need to be treated to prevent one event. CVD denotes cardiovascular; HR, hazard ratio; MACE, major adverse cardiovascular events. RAASi, renin-angiotensin aldosterone inhibitors; SGLT2i, sodium-glucose cotransporter-2 inhibitors.

For peer review only

For peer review only - http://bmjopen.bmj.com/site/about/guidelines.xhtml

DISCUSSION

In a background population of ~3.8 million people from Sweden's Stockholm and Skåne regions, this study identified 99,382 patients with undiagnosed CKD in a six-year period that ended in 2020. Of that population, CKD remained undiagnosed by healthcare in two out of three patients five years after CKD could be identified using available laboratory measurements. Those with more advanced CKD were diagnosed faster than those with less severe CKD, seemingly irrespective of age. Use of guideline-directed pharmacological therapy was not dramatically affected by a given CKD diagnosis, seemingly irrespective of the severity of the CKD; and there was very little use of SGLT2i across the entire study population, as anticipated during this observation period. Simulations of guideline-directed pharmacological therapy indicated that there is potential to substantially improve outcomes in CKD, with the greatest projected protective effects resulting from the use of statins, RAASi and SGLT2i in combination.

What opportunities are currently lost in chronic kidney disease?

A CKD diagnosis would be anticipated to lead to several changes in care, such as more structured follow-up, changed dosing of several drug classes, and more preventive measures taken at contrast radiological examinations, to name but a few.

No striking changes to treatment regimens were observed around the time of a diagnosis of CKD in healthcare, suggesting that clinical practice didn't always follow the clinical practice guidelines informing practitioners during the study period, such as those published by KDIGO in 2012 and 2013,(8,9,11) and national guidelines and recommendations. Indeed, the use of such pharmacological treatments is pertinent to this study's population, where the proportion of patients that was affected by MACE was far greater than the proportion that died of non-cardiovascular causes. The underuse of guideline-directed pharmacological therapy is consistent with recent large, cohort studies, which also reported that only small proportions of patients with CKD were using statins, RAASi or SGLT2i.(1,10,11) Given that SGLT2i have only recently been included in clinical practice guidelines after the study period,(7,11–13) low use of these agents was in line with expectations. Patients with CKD stages A2 and A3 more often used SGLT2i than those with stages S3a to S5, both at baseline and during follow-up (Table 1 and Figure 3), explained by their more than twice as high prevalence of diabetes (Table 1). In sum, our data clearly show that opportunities are lost in both the diagnosis and treatment of CKD.

It should be noted that diagnosing and treating CKD is likely less incentivized in Sweden than in many other countries. A CKD diagnosis has smaller effects on rewards for healthcare units in Sweden, where a government-funded healthcare system covers all citizens, than it would have under insurance/claims systems.(28) Further, some other countries with similar healthcare systems have much stronger encouragement for registering a CKD diagnosis and providing evidence-based CKD care. The United Kingdom, for example, had a focus on registering CKD diagnoses and providing evidence-based preventive care since 2006, as part of the primary care Quality and Outcomes Framework.(29)

What are the windows of opportunity in chronic kidney disease?

For peer review only - http://bmjopen.bmj.com/site/about/guidelines.xhtml

Simulations of pharmacological treatments indicated that, with greater adherence to guidelines by the caring physician and adequate medication adherence by the patient, there is significant potential to improve outcomes in CKD. Addressing cardiovascular disease risk is central to CKD treatment.(7) Lowering of blood pressure and lipids in patients with CKD has robust evidence for the prevention of both cardiovascular disease events and death;(22,23,30) and evidence is accumulating that SGLT2i reduces the risk of cardiorenal complications (heart failure and CKD) and all-cause mortality in all patients with CKD, irrespective of whether they have diabetes or not.(12,13,31) Combinations of these treatments are likely to have multiplicative protective effects.(25,26) Therefore, there is little reason, apart from side effects, to not use all three treatments in combination from the time of CKD diagnosis. On the contrary, RAASi and SGLT2i seem to have additive effects when used in combination. In the current study, adding an SGLT2i to the treatment regimen provided the largest contribution to the combined treatment effect of the scenarios tested (Figure 4).

While greater use of evidence-based treatment is likely the clearest opportunity for improving outcomes in CKD, deficiencies in the diagnosis of CKD also merit addressing. It's likely that the laboratory analyses used for CKD definitions in the present study are routinely performed for an array of conditions and complaints, but unless the caring physician is specifically looking for kidney disease, the historic data needed to make a CKD diagnosis may be overlooked. Although our findings are exclusively based on Swedish health care data, the low rate of CKD diagnosis in patients who fulfill CKD criteria has been described in numerous other health care systems globally.(1) Future research might trial systems where eGFR and UACR values in databases are automatically monitored, notifying the caring physicians if a patient presents abnormal measurements meeting the criteria for CKD. This concept has already been implemented in acute kidney injury, where electronic alert systems monitor electronic health records.(32,33) An automated system, such as that, may be the most reliable way to address the issue of human oversight, the likely reason so many cases of CKD went undiagnosed. Indeed, automated systems could theoretically be applied in multiple scenarios, from small databases within private health clinics to large national or regional databases, providing the capacity for continuous population-wide opportunistic screening; as compared to the current system, in which screening is only recommended in high-risk populations (i.e., diabetes, hypertension and previously known cardiovascular disease).(34)

Strengths and limitations

This study had access to health registries and electronic health records detailing most incidences of healthcare and medication use by residents in two of Sweden's largest regions, representing over a third of the national population. Despite its strength in its capacity to identify patients relevant to the study aims and to characterize the subsequent, large study population, several limitations must be acknowledged.

The identification of undiagnosed CKD was dependent on patients providing samples for laboratory analyses. Since only persons with suspected or known other disease have provided the samples necessary to make a laboratory-based assessment of CKD status, it is likely that this study underestimates the number of patients with undiagnosed CKD. It is possible that some diagnosing has taken place outside of the utilized data source. This

For peer review only - http://bmjopen.bmj.com/site/about/guidelines.xhtml

would instead lead to an underestimation of the number of patients with a diagnosis of CKD. The follow-up period varied between patients, and in some cases, patients may have been enrolled towards the end of the inclusion period, leaving little opportunity for a diagnosis of CKD in healthcare. However, the methods used take person-time and censoring into account. It must also be acknowledged that this study couldn't assess if patients from a specific socioeconomic background or ethnicity were disproportionally affected by the deficiencies in CKD-related healthcare.

Simulations of guideline-directed pharmacological therapy assumed that treatment was initiated at the time CKD was identified using laboratory measurements and that the adherence to treatment would be the same as that obtained in clinical trials. While indicating the full preventive potential, such efficiency and success in treatment is unlikely in routine clinical practice. The simulations assumed that the treatment effects observed in meta-analyses of each medication of interest were replicable in this study's untreated populations and that conjoint effects on outcomes did not deviate from multiplicativity, which has been demonstrated for RAASi and statins,(25,26) but not robustly for SGLT2i in relation to the other two drug classes.

Finally, the high age of the population merits some discussion. It can be debated if the age-related fall in eGFR should be viewed as glomerular senescence rather than kidney disease that uniformly requires treatment, and if age-agnostic diagnostic thresholds should be used.(35) In the absence of guideline recommendations for treating elderly persons differently, we have taken a similar approach as one would to hypertension or heart failure, and have assumed similar potential relative gains for all ages. Sensitivity analyses limiting the sample to younger persons provided less than half of the potential effect on the absolute scale than the analyses of the whole age distribution.

Conclusion

Chronic kidney disease remained undiagnosed in two out of three patients over half a decade in routine healthcare, despite readily available data indicating its development. This highlights the need for automated systems that continuously monitor electronic health records and alert physicians when CKD has developed in a patient; eliminating any reliance on the physician to, firstly, decide to search for the disease and to then make a diagnosis. Those who do receive a diagnosis get insufficient guideline-directed pharmacological therapy with statins, RAASi, and SGLT2i, collectively potentiating a situation where CKD won't be addressed in healthcare until more advanced disease develops. Despite these lost opportunities, this study indicates that there is significant potential to substantially improve outcomes in CKD with stricter adherence to clinical practice guidelines by both the treating physicians and the patients.

For peer review only - http://bmjopen.bmj.com/site/about/guidelines.xhtml

AUTHOR CONTRIBUTIONS

J.S., A.N., S.K., J.B, S.G., T.C:, M.K.S., and J.Ä. developed the concept of the study. S.G and T.C performed all data curation and statistical analyses. J.S., A.N., S.K., S.G., T.C:, M.K.S., and J.Ä. participated in data interpretation. J.S. drafted the original manuscript, with critical intellectual input from A.N., S.K., J.B, S.G., T.C:, M.K.S., and J.Ä.

ACKNOWLEDGEMENTS

The authors thank Jordan Loader PhD, of Sence, Uppsala, Sweden, for providing medical writing support, which was funded by AstraZeneca, Stockholm, Sweden, in accordance with Good Publication Practice (GPP3) guidelines (http://www.ismpp.org/gpp3). The authors also thank Susanna Jerström and Pål Hasvold from AstraZeneca for valuable comments on the manuscript.

FUNDING

The study was fully sponsored by AstraZeneca N/A.

COMPETING INTERESTS

J.S. reports stock ownership in Anagram kommunikation AB and Symptoms Europe AB. A.N. has participated as a consultant on advisory board committees and educational activities with Astra Zeneca, Novo Nordisk, Boehringer Ingelhim and Eli Lilly. S.K. has participated as a consultant on advisory board committees and educational activities with AstraZeneca and Novo Nordisk. J.B. is an employee of AstraZeneca. T.C. is an employee and stockowner in Sence Research AB (an independent company in epidemiology and outcomes research) that received funding from AstraZeneca for statistical analysis within this research project. M.K.S. has participated as a consultant on advisory board committees and educational activities with Amgen, AstraZeneca, Boehringer Ingelheim, GSK, and NovoNordisk. J.Ä. has received payment/honoraria from AstraZeneca and Novartis; participated on a data safety monitoring/advisory board for AstraZeneca, Astella and Boehringer Ingelheim. The other authors report no competing interests.

DATA SHARING

Data sources utilised in this project are subject to ethical and privacy restrictions in Sweden. Therefore, the data that support the findings of this study are not available on request.

For peer review only - http://bmjopen.bmj.com/site/about/guidelines.xhtml

REFERENCES

1. Sundström J, Bodegard J, Bollmann A, Vervloet MG, Mark PB, Karasik A, et al. Prevalence, outcomes, and cost of chronic kidney disease in a contemporary population of 2·4 million patients from 11 countries: The CaReMe CKD study. The Lancet Regional Health - Europe. 2022 Sep 1;20:100438.
2. GBD Chronic Kidney Disease Collaboration. Global, regional, and national burden of chronic kidney disease, 1990-2017: a systematic analysis for the Global Burden of Disease Study 2017. Lancet. 2020 Feb 29;395(10225):709–33.
3. Hill NR, Fatoba ST, Oke JL, Hirst JA, O'Callaghan CA, Lasserson DS, et al. Global Prevalence of Chronic Kidney Disease - A Systematic Review and Meta-Analysis. PLoS One. 2016;11(7):e0158765.
4. Chen TK, Knicely DH, Grams ME. Chronic Kidney Disease Diagnosis and Management: A Review. JAMA. 2019 Oct 1;322(13):1294–304.
5. Saran R, Robinson B, Abbott KC, Agodoa LYC, Bhave N, Bragg-Gresham J, et al. US Renal Data System 2017 Annual Data Report: Epidemiology of Kidney Disease in the United States. American Journal of Kidney Diseases. 2018 Mar;71(3):A7.
6. Saran R, Robinson B, Abbott KC, Bragg-Gresham J, Chen X, Gipson D, et al. US Renal Data System 2019 Annual Data Report: Epidemiology of Kidney Disease in the United States. American Journal of Kidney Diseases. 2020 Jan;75(1):A6–7.
7. de Boer IH, Khunti K, Sadusky T, Tuttle KR, Neumiller JJ, Rhee CM, et al. Diabetes Management in Chronic Kidney Disease: A Consensus Report by the American Diabetes Association (ADA) and Kidney Disease: Improving Global Outcomes (KDIGO). Diabetes Care. 2022 Oct 3;45(12):3075–90.
8. Levin A, Stevens PE. Summary of KDIGO 2012 CKD Guideline: behind the scenes, need for guidance, and a framework for moving forward. Kidney Int. 2014 Jan;85(1):49–61.
9. Wanner C, Tonelli M, Kidney Disease: Improving Global Outcomes Lipid Guideline Development Work Group Members. KDIGO Clinical Practice Guideline for Lipid Management in CKD: summary of recommendation statements and clinical approach to the patient. Kidney Int. 2014 Jun;85(6):1303–9.
10. Tuttle KR, Alicic RZ, Duru OK, Jones CR, Daratha KB, Nicholas SB, et al. Clinical Characteristics of and Risk Factors for Chronic Kidney Disease Among Adults and Children: An Analysis of the CURE-CKD Registry. JAMA Netw Open. 2019 Dec 2;2(12):e1918169.
11. Tangri N, Svensson MK, Bodegård J, Eryd SA, Thuresson M, Gustafsson S, et al. Mortality, Healthcare Burden, and Treatment of Chronic Kidney Disease – A Multinational, Observational Study (OPTIMISE-CKD). Kidney360. 2024 Jan 23;10.34067/KID.0000000000000374.
12. Heerspink HJL, Stefánsson BV, Correa-Rotter R, Chertow GM, Greene T, Hou FF, et al. Dapagliflozin in Patients with Chronic Kidney Disease. N Engl J Med. 2020 Oct 8;383(15):1436–46.
13. The EMPA-KIDNEY Collaborative Group, Herrington WG, Staplin N, Wanner C, Green JB, Hauske SJ, et al. Empagliflozin in Patients with Chronic Kidney Disease. N Engl J Med. 2023 Jan 12;388(2):117–27.
14. Anell A. The public-private pendulum--patient choice and equity in Sweden. N Engl J Med. 2015 Jan 1;372(1):1–4.

For peer review only

15. Ludvigsson JF, Otterblad-Olausson P, Pettersson BU, Ekbom A. The Swedish personal identity number: Possibilities and pitfalls in healthcare and medical research. European Journal of Epidemiology. 2009;24(11):659–67.
16. World Health Organization. WHO Collaborating Centre for Drug Statistics Methodology. ATC/DDD Index 2017. Available from: https://www.whocc.no/. Accessed December 9, 2022.
17. World Health Organization. International classification of disease and related health problems. Available from: http://www.who.int/classifications/icd/en/. Accessed December 9, 2022.
18. Nordic Medico-Statistical Committee. NOMESCO Classification of Surgical Procedures. 2010. Available from: https://norden.diva-portal.org/smash/get/diva2:970547/FULLTEXT01.pdf. Accessed December 9, 2022.
19. Nyman U, Grubb A, Larsson A, Hansson LO, Flodin M, Nordin G, et al. The revised Lund-Malmö GFR estimating equation outperforms MDRD and CKD-EPI across GFR, age and BMI intervals in a large Swedish population. Clin Chem Lab Med. 2014 Jun;52(6):815–24.
20. R Development Core Team Core Team. R: A language and environment for statistical computing. (2011).
21. Carstensen B, Plummer M. Using Lexis Objects for Multi-State Models in R. Journal of Statistical Software. 2011 Jan 4;38:1–18.
22. Hou W, Lv J, Perkovic V, Yang L, Zhao N, Jardine MJ, et al. Effect of statin therapy on cardiovascular and renal outcomes in patients with chronic kidney disease: a systematic review and meta-analysis. Eur Heart J. 2013 Jun;34(24):1807–17.
23. Balamuthusamy S, Srinivasan L, Verma M, Adigopula S, Jalandhara N, Jalandara N, et al. Renin angiotensin system blockade and cardiovascular outcomes in patients with chronic kidney disease and proteinuria: a meta-analysis. Am Heart J. 2008 May;155(5):791–805.
24. Mavrakanas TA, Tsoukas MA, Brophy JM, Sharma A, Gariani K. SGLT-2 inhibitors improve cardiovascular and renal outcomes in patients with CKD: a systematic review and meta-analysis. Sci Rep. 2023 Sep 23;13(1):15922.
25. Sundström J, Gulliksson G, Wirén M. Synergistic effects of blood pressure-lowering drugs and statins: systematic review and meta-analysis. BMJ Evid Based Med. 2018 Apr;23(2):64–9.
26. Kanukula R, Esam H, Sundström J, Rodgers A, Salam A. Does Co-administration of Antihypertensive Drugs and Statins Alter Their Efficacy and Safety? A Systematic Review and Meta-analysis of Randomized Controlled Trials. J Cardiovasc Pharmacol. 2019 Jun;73(6):352–8.
27. Wald DS, Law M, Morris JK, Bestwick JP, Wald NJ. Combination therapy versus monotherapy in reducing blood pressure: meta-analysis on 11,000 participants from 42 trials. Am J Med. 2009 Mar 1;122(3):290–300.
28. Tangri N, Franzén S, Peach EJ, Barone S, Kushner PR. REVEAL CKD: Estimated Glomerular Filtration Rate (eGFR) Decline Before and After a CKD Diagnosis Among Patients With CKD Stage 3. Presented at the American Society of Nephrology Kidney Week 2022, Orlando, FL, USA, November 3–6. 2022;
29. Stevens PE, O'Donoghue DJ. The UK model for system redesign and chronic kidney disease services. Semin Nephrol. 2009 Sep;29(5):475–82.

For peer review only - http://bmjopen.bmj.com/site/about/guidelines.xhtml

30. Qin Y, Chen T, Chen Q, Lv JY, Qi N, Wu C, et al. The effect of angiotensin-converting enzyme inhibitor/angiotensin receptor blocker use on mortality in patients with chronic kidney disease: a meta-analysis of observational studies. Pharmacoepidemiol Drug Saf. 2016 May;25(5):503–11.
31. Wheeler DC, Stefánsson BV, Jongs N, Chertow GM, Greene T, Hou FF, et al. Effects of dapagliflozin on major adverse kidney and cardiovascular events in patients with diabetic and non-diabetic chronic kidney disease: a prespecified analysis from the DAPA-CKD trial. Lancet Diabetes Endocrinol. 2021 Jan;9(1):22–31.
32. Kashani KB. Automated acute kidney injury alerts. Kidney Int. 2018 Sep;94(3):484–90.
33. Breighner CM, Kashani KB. Impact of e-alert systems on the care of patients with acute kidney injury. Best Pract Res Clin Anaesthesiol. 2017 Sep;31(3):353–9.
34. Nationellt programområde. Nationellt vårdprogram för kronisk njursjukdom. 2022 [cited 2023 Jan 15]; Available from: https://d2flujgsl7escs.cloudfront.net/external/Nationellt_vardprogram_for_kronisk_njursjukdom.pdf
35. Delanaye P, Jager KJ, Bökenkamp A, Christensson A, Dubourg L, Eriksen BO, et al. CKD: A Call for an Age-Adapted Definition. J Am Soc Nephrol. 2019 Oct;30(10):1785–805.

For peer review only

For peer review only - http://bmjopen.bmj.com/site/about/guidelines.xhtml

FIGURE LEGENDS

Figure 1. Time to first chronic kidney disease (CKD) diagnosis in healthcare after CKD was identified using measurements of estimated glomerular filtration rate (eGFR)

Kaplan-Meier curves. Patients are stratified by the KDIGO-defined stage of their CKD and by age group. S3a indicates eGFR 45-59 mL/min/1.73m^2; S3b, eGFR 30-44 mL/min/1.73m^2; S4, eGFR 15-29 mL/min/1.73m^2; S5, eGFR < 15 mL/min/1.73m^2.

Figure 2. Time to first chronic kidney disease (CKD) diagnosis in healthcare after CKD was identified using measurements of urinary albumin-creatinine ratio (UACR)

Kaplan-Meier curves. Patients are stratified by the KDIGO-defined stage of their CKD and by age group. All patients from the total cohort whose CKD could be identified using abnormal UACR measurements according to the KDIGO guidelines were included in these analyses, regardless of whether measurements of estimated glomerular filtration rate were available. A2 indicates UACR 3-30 g/mol; A3, UACR > 30 g/mol.

Figure 3. Multi-state models presenting the proportions of patients diagnosed or not diagnosed with chronic kidney disease (CKD) in healthcare that used or didn't use guideline-directed pharmacological therapy

Use of **A)** statins, **B)** renin-angiotensin aldosterone system inhibitors (RAASi), and **C)** sodium-glucose cotransporter-2 inhibitors (SGLT2i) were assessed. Patients are stratified by the KDIGO-defined stage of their CKD, identified using estimated glomerular filtration rate (Stages S3a, S3b, and S4+S5) and urinary albumin-creatinine ratio (Stages A2 and A3).

Figure 4. Sensitivity analyses demonstrating variable treatment effect in less-than-optimal settings

Dashed, horizontal lines indicate optimal results (multiplicative effects of all drugs assumed, using all drugs and all age groups). Multipl., multiplicative effects of all drugs assumed; Multipl., no SGLT2i, multiplicative effects of all drugs assumed, but HR for SGLT2i set to 1; Single drug, no multiplicative effects of drugs assumed, only the HR from the drug with the largest effect counted, HR for all other drugs set to 1; Age<=71, results only from subset of persons in lowest fourth of age (71 years); All ages+T2D/HTN, results only from subset of persons with type-2 diabetes or hypertension; Surv>90d, results only from subset that survived more than 90 days after index.

For peer review only - http://bmjopen.bmj.com/site/about/guidelines.xhtml

For peer review only

For peer review only - http://bmjopen.bmj.com/site/about/guidelines.xhtml

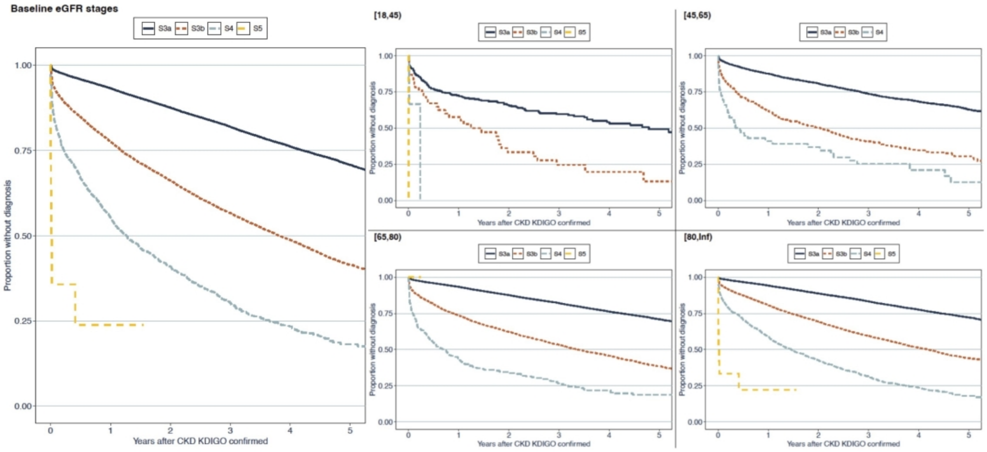

Figure 1. Time to first chronic kidney disease (CKD) diagnosis in healthcare after CKD was identified using measurements of estimated glomerular filtration rate (eGFR)
Kaplan-Meier curves. Patients are stratified by the KDIGO-defined stage of their CKD and by age group. S3a indicates eGFR 45-59 mL/min/1.73m2; S3b, eGFR 30-44 mL/min/1.73m2; S4, eGFR 15-29 mL/min/1.73m2; S5, eGFR < 15 mL/min/1.73m2.

236x111mm (300 x 300 DPI)

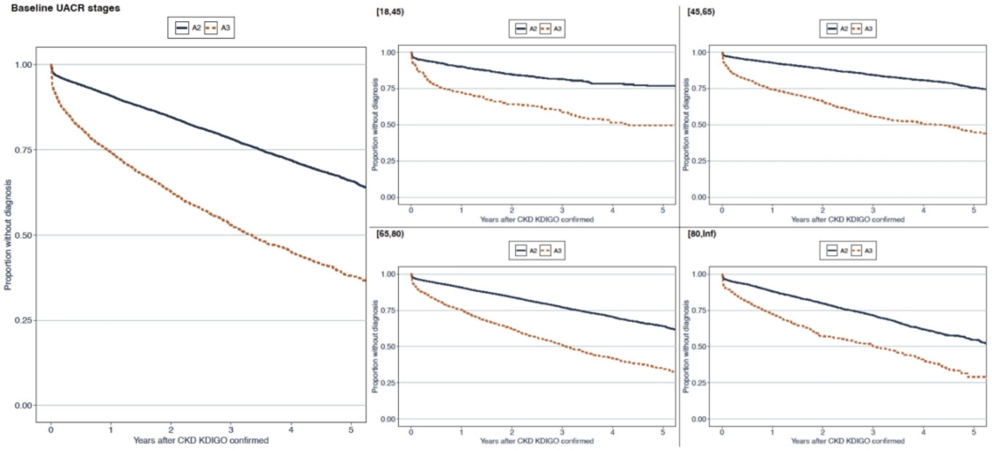

Figure 2. Time to first chronic kidney disease (CKD) diagnosis in healthcare after CKD was identified using measurements of urinary albumin-creatinine ratio (UACR)
Kaplan-Meier curves. Patients are stratified by the KDIGO-defined stage of their CKD and by age group. All patients from the total cohort whose CKD could be identified using abnormal UACR measurements according to the KDIGO guidelines were included in these analyses, regardless of whether measurements of estimated glomerular filtration rate were available. A2 indicates UACR 3-30 g/mol; A3, UACR > 30 g/mol.

229x105mm (300 x 300 DPI)

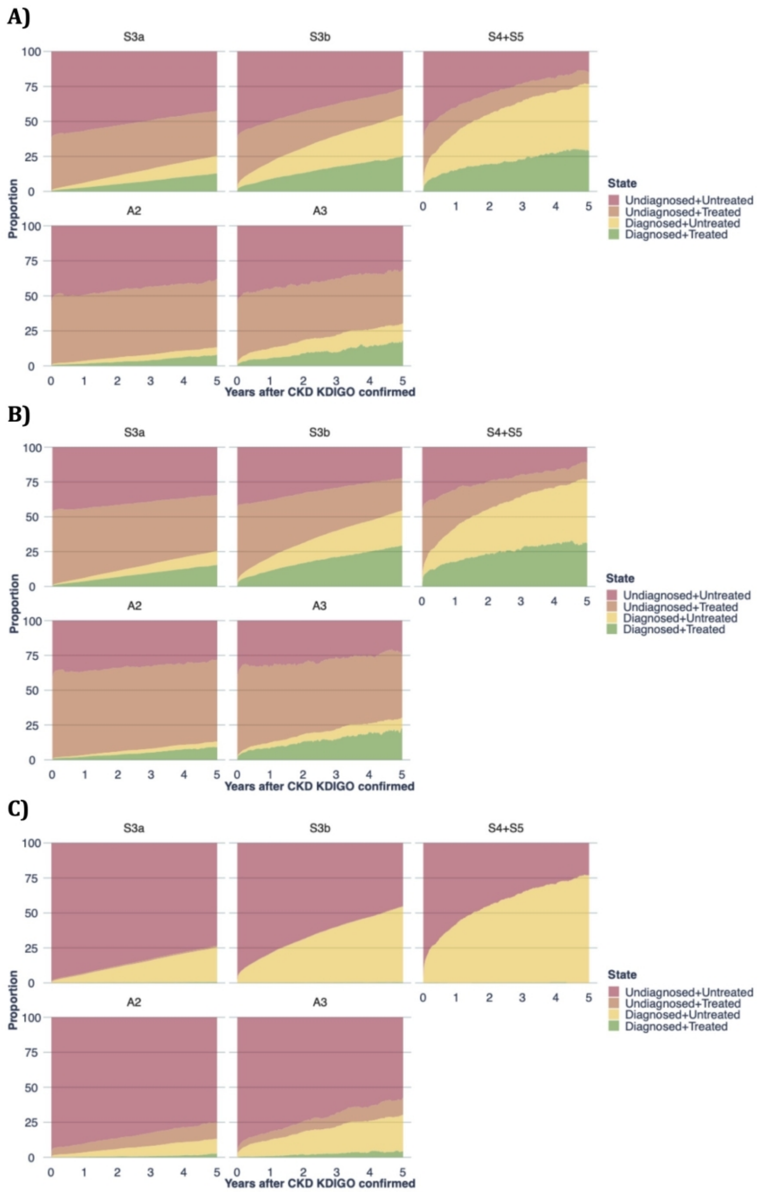

Figure 3. Multi-state models presenting the proportions of patients diagnosed or not diagnosed with chronic kidney disease (CKD) in healthcare that used or didn't use guideline-directed pharmacological therapy Use of A) statins, B) renin-angiotensin aldosterone system inhibitors (RAASi), and C) sodium-glucose cotransporter-2 inhibitors (SGLT2i) were assessed. Patients are stratified by the KDIGO-defined stage of their CKD, identified using estimated glomerular filtration rate (Stages S3a, S3b, and S4+S5) and urinary albumin-creatinine ratio (Stages A2 and A3).

145x228mm (300 x 300 DPI)

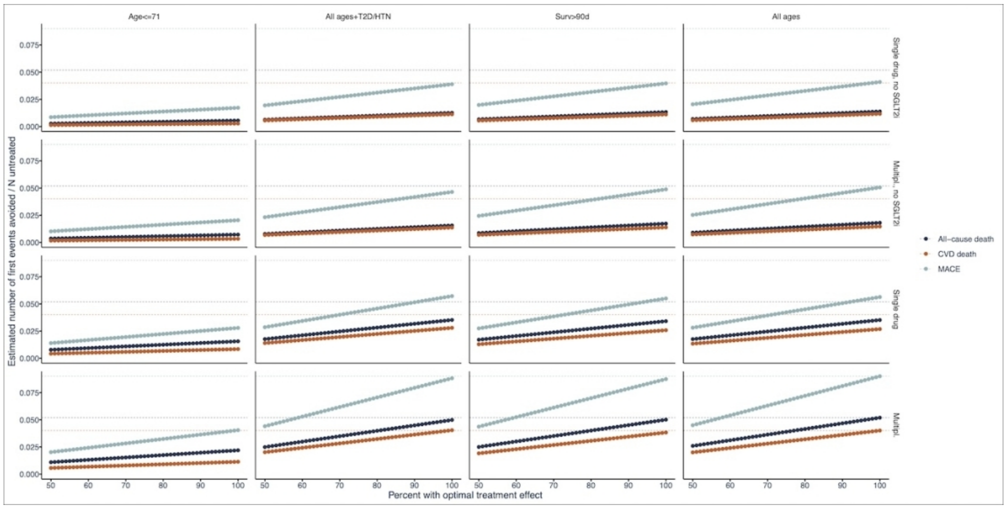

Figure 4. Sensitivity analyses demonstrating variable treatment effect in less-than-optimal settings
Dashed, horizontal lines indicate optimal results (multiplicative effects of all drugs assumed, using all drugs and all age groups). Multipl., multiplicative effects of all drugs assumed; Multipl., no SGLT2i, multiplicative effects of all drugs assumed, but HR for SGLT2i set to 1; Single drug, no multiplicative effects of drugs assumed, only the HR from the drug with the largest effect counted, HR for all other drugs set to 1; Age<=71, results only from subset of persons in lowest fourth of age (71 years); All ages+T2D/HTN, results only from subset of persons with type-2 diabetes or hypertension; Surv>90d, results only from subset that survived more than 90 days after index.

112x56mm (300 x 300 DPI)

Lost opportunities in chronic kidney disease

Supplemental Material

Contents

Supplemental Methods. The CELOSIA database 2

Table S1. Meta-analyses that assessed the effect of first-line pharmacological treatments on the risk of major adverse cardiovascular events in patients with chronic kidney disease, with or without diabetes 3

Table S2. Inclusion criteria for the CELOSIA database from which the study sample was extracted 4

Table S3. Stages of chronic kidney disease diagnosed using measurements of eGFR and UACR according to the KDIGO clinical practice guidelines 6

Table S4. International Classification of Diseases (ICD)-10 codes used to search for diagnoses of CKD, prevalent comorbidities, and outcomes of interest 7

Table S5. Anatomical Therapeutic Chemical (ATC) classification system codes used to search for treatments of interest in this study's population........ 8

Table S6. Baseline characteristics of the total study cohort stratified by age group 9

Table S7. Proportions of patients diagnosed with chronic kidney disease in healthcare within five years of the development of the disease 11

Figure S1. Participant flow 12

Figure S2. Multi-state models presenting the proportions of patients diagnosed or not diagnosed with chronic kidney disease (CKD) in healthcare who received or didn't receive guideline-directed pharmacological therapy, including the terminal, absorbing outcome, all-cause death . 13

Figure S3. Multi-state models presenting the proportions of patients diagnosed or not diagnosed with chronic kidney disease (CKD) in healthcare who received or didn't receive guideline-directed pharmacological therapy, including the terminal, absorbing outcomes, major adverse cardiovascular events (MACE) and non-cardiovascular disease (CVD) death 14

Figure S4. Multi-state models presenting the proportions of patients diagnosed or not diagnosed with chronic kidney disease (CKD) in healthcare who received or didn't receive guideline-directed pharmacological therapy, including the terminal, absorbing outcome, all-cause death . 15

Figure S5. Multi-state models presenting the proportions of patients diagnosed or not diagnosed with chronic kidney disease (CKD) in healthcare who received or didn't receive guideline-directed pharmacological therapy, including the terminal, absorbing outcomes, major adverse cardiovascular events (MACE) and non-cardiovascular disease (CVD) death 16

References 17

For peer review only - http://bmjopen.bmj.com/site/about/guidelines.xhtml

Supplemental Methods. The CELOSIA database

The CELOSIA database, from which this present study's sample was extracted, includes all Swedish residents aged ≥18 years who were diagnosed with chronic kidney disease (CKD), heart failure or diabetes mellitus between January 1st, 2000, and December 31st, 2020.

Patients were identified from several sources with national coverage, including the Swedish Prescribed Drug Registry, the National Patient Registry, and the National Death Registry. The Swedish Prescribed Drug Registry logs each resident's filled drug prescriptions according to the Anatomical Therapeutic Chemical [ATC] classification system.[1] The National Patient Registry and the National Death Registry records diagnoses, surgical procedures, and causes of death using the International Classification of Diseases [ICD][2] system and the Nordic Medico-Statistical Committee Classification of Surgical Procedure system.[3]

Patients were also identified from several data sources with regional coverage, including the Healthcare Data Warehouse of Region Stockholm, the Healthcare Data Warehouse of Region Skåne Melior, and the Profdoc Medical Office of Region Skåne. Additionally, the CELOSIA database utilized electronic health records from Region Stockholm and Region Skåne to diagnose patients with CKD using laboratory measurements.

A complete list of the ATC codes, ICD-10 codes, procedure codes, and laboratory values used to identify these patients is available in Supplemental Table 2. Patients were required to have a 12-digit personal identity number, an identity number unique to all Swedish residents,[4] enabling data to be linked between the different sources.

The total population with data available for the present study was ~3.8 million people, corresponding to 36% of the Swedish population.

For peer review only - http://bmjopen.bmj.com/site/about/guidelines.xhtml

Table S1. Meta-analyses that assessed the effect of first-line pharmacological treatments on the risk of major adverse cardiovascular events in patients with chronic kidney disease, with or without diabetes

Study	Drug	Statistic	All-cause mortality	MACE	CVD mortality	Myocardial infarction	Stroke	Hospitalization for HF
Qin et al., 2016[5]	RAASi	HR (95%CI)	0.83 (0.78-0.87)					
Balamuthusamy et al., 2008[6]	RAASi	RR (95%CI)	1.03 (0.92-1.04)	0.91 (0.85-0.98)	1.12 (0.95-1.32)	0.82 (0.67-1.00)	1.02 (0.81-1.28)	0.78 (0.66-0.92)
Xie et al., 2016[7]	ACEi	OR (95%CI)	0.87 (0.74-1.01)	0.82 (0.71-0.92)	0.88 (0.72-1.09)			
Zhang et al., 2020[8]	ACEi	OR (95%CI)	0.77 (0.66-0.91)	0.73 (0.64-0.84)	0.73 (0.63-0.86)			
Ninomiya et al., 2013[9]	ACEi	HR (95%CI)		0.81 (0.73-0.90)				
Xie et al., 2016[7]	ARBs	OR (95%CI)	0.99 (0.78-1.21)	0.76 (0.62-0.89)	1.12 (0.80-1.58)			
Zhang et al., 2020[8]	ARBs	OR (95%CI)	1.01 (0.82-1.25)	0.83 (0.70-0.98)	1.16 (0.88-1.53)			
Major et al., 2015[10]	Statins	RR (95%CI)	0.66 (0.49-0.88)	0.59 (0.48-0.72)			0.56 (0.28-1.13)	
Su et al., 2016[11]	Statins	OR (95%CI)		0.69 (0.61-0.79)				
Upadhyay et al., 2012[12]	Statins	RR (95%CI)	0.88 (0.79-0.98)	0.75 (0.68-0.84)				
Strippoli et al., 2008[13]	Statins	RR (95%CI)	0.81 (0.74-0.89)		0.80 (0.70-0.90)			
Palmer et al., 2014[14]	Statins	RR (95%CI)	0.81 (0.74-0.88)	0.76 (0.73-0.80)	0.78 (0.68-0.89)	0.55 (0.42-0.72)	0.61 (0.38-0.98)	
Hou et al., 2013[15]	Statins	RR (95%CI)	0.92 (0.85-0.99)	0.77 (0.70-0.85)	0.91 (0.84-0.99)		0.79 (0.56-1.12)	
Zhang et al., 2014[16]	Statins	RR (95%CI)	0.79 (0.72-0.86)	0.76 (0.72-0.80)	0.83 (0.73-0.93)	0.66 (0.52-0.83)	0.70 (0.57-0.85)	
Barylski et al., 2013[17]	Statins	RR (95%CI)	0.66 (0.55-0.79)	0.55 (0.40-0.75)	0.69 (0.55-0.78)		0.66 (0.50-0.88)	
Yamada et al., 2021[18]	SGLT-2i	RR (95%CI)		0.87 (0.76-1.00)				
Salah et al., 2021[19]	SGLT-2i	HR (95%CI)	0.82 (0.67-1.00)		0.84 (0.68-1.03)	0.72 (0.54-0.97)	0.67 (0.33-1.36)	0.61 (0.48-0.77)
Lin et al., 2021[20]	SGLT-2i	RR (95%CI)		0.84 (0.82-0.97				
Kamdar et al., 2021[21]	SGLT-2i	HR (95%CI)	0.89 (0.81-0.98)		0.90 (0.79-1.02)	0.82 (0.64-1.05)		0.69 (0.63-0.75)
D'Andrea et al., 2020[22]	SGLT-2i	HR (95%CI)		0.82 (0.69-0.97)				
Staplin et al., 2021[23]	SGLT-2i	RR (95%CI)	0.94 (0.75-1.12)	0.84 (0.76-0.93)	0.84 (0.73-0.97)			
Malik et al., 2020[24]	SGLT-2i	HR (95%CI)		0.80 (0.69-0.92)	0.88 (0.71-1.09)	0.78 (0.62-0.97)	0.83 (0.62-1.11)	0.61 (0.47-0.77)
Cao et al., 2021[25]	SGLT-2i	HR (95%CI)	0.83 (0.55-1.27)	0.75 (0.60-0.93)	0.89 (0.60-1.30)			0.74 (0.55-0.99)
Kaze et al., 2022[26]	SGLT-2i	HR (95%CI)	0.86 (0.77-0.96)	0.83 (0.75-0.93)	0.84 (0.74-0.96)	0.78 (0.67-0.92)	0.76 (0.59-0.97)	0.62 (0.55-0.71)
Yang et al., 2022[27]	SGLT-2i	OR (95%CI)	0.79 (0.66-0.93)		0.80 (0.65-0.98)			0.57 (0.45-0.72)

ACEi denotes angiotensin converting enzyme inhibitors; ARBs, angiotensin receptor blockers; CI, confidence interval; CVD, cardiovascular disease; HF, heart failure; HR, hazard ratio; MACE, major adverse cardiovascular events; OR, odds ratio; RAASi, renin-angiotensin aldosterone system inhibitors; RR, risk ratio; SGLT-2i, sodium-glucose cotransporter-2 inhibitors.

For peer review only - http://bmjopen.bmj.com/site/about/guidelines.xhtml

Table S2. Inclusion criteria for the CELOSIA database from which the study sample was extracted

Inclusion criteria (any of)	Description
ICD10: I50	Heart failure
ICD10: I11.0	Hypertensive heart disease with (congestive) heart failure
ICD10: I13.0	Hypertensive heart and renal disease with (congestive) heart failure
ICD10: I13.2	Hypertensive heart and renal disease with both (congestive) heart failure and renal failure
ICD10: I25.5	Ischaemic cardiomyopathy
ICD10: I42.0	Dilated cardiomyopathy
ICD10: I42.6	Alcoholic cardiomyopathy
ICD10: I42.9	Cardiomyopathy, unspecified
ICD10: I43.1	Cardiomyopathy in metabolic diseases
ICD10: Z99.4	Dependence on artificial heart
Procedure: FQA	Transplantation of heart
Procedure: FQB	Transplantation of heart and lung
Procedure: FPE26	Implantation of transvenous cardiac pacemaker with biventricular electrodes
ATC: C01CX08	Levosimendan
BNP >100ng/L	Laboratory value
NT-proBNP >300ng/L	Laboratory value
ICD-10: E10.2	Type I diabetes mellitus with renal complications
ICD-10: E11.2	Type II diabetes mellitus with renal complications
ICD-10: E12.2	Malnutrition-related diabetes mellitus with renal complications
ICD-10: E13.2	Other specified diabetes mellitus with renal complications
ICD-10: E14:2	Unspecified diabetes mellitus with renal complications
ICD-10: N08.3	Glomerular disorders in diabetes mellitus
ICD-10: N17	Acute renal failure
ICD-10: N18	Chronic kidney failure
ICD-10: N19	Unspecified kidney failure
ICD-10: I12.0	Glomerular disorders in diabetes mellitus
ICD-10: I12.9	Hypertensive renal disease without renal failure
ICD-10: I13.1	Hypertensive heart and renal disease with renal failure
ICD-10: I13.9	Hypertensive heart and renal disease, unspecified
ICD-10: Z49.1	Extracorporeal dialysis
ICD-10: Z49.2	Other dialysis
ICD-10: Z99.2	Dependence on renal dialysis
Procedure: JAK10	Laparotomy and insertion of peritoneal dialysis catheter
Procedure: TJA33	Percutaneous introduction of peritoneal dialysis catheter
Procedure: TJA35	Removal of peritoneal dialysis catheter
Procedure: DJ008	Laparoscopic dialysis catheter insertion
Procedure: DR013	Initiation of continuos ambulatory peritoneal dialysis (CAPD)
Procedure: DR014	Hemodiafiltration (HDF)
Procedure: DR015	Hemodialysis, acute
Procedure: DR016	Hemodialysis, chronic
Procedure: DR017	Hemofiltration

For peer review only

For peer review only - http://bmjopen.bmj.com/site/about/guidelines.xhtml

Procedure: DR023	Peritonealdialysis, acute
Procedure: DR024	Peritonealdialysis, chronic
Procedure: DR055	Citrate dialysis
Procedure: DR056	Heparin free dialysis
Procedure: DR060	Home hemodialysis control
Procedure: DR061	Home hemodialysis start
Procedure: KAS10	Allogenic transplantation of kidney from cadaver donor
Procedure: KAS20	Allogenic transplantation of kidney from living donor
Procedure: QF006	Peritoneal dialysis
eGFR <60 mL/min/1.73m^2	Laboratory value
U-albumin/creatinine ratio > 3 mg/mmol	Laboratory value
ICD-10: E10	Type I diabetes mellitus
ICD-10: E11	Type II diabetes mellitus
ICD-10: E12	Malnutrition-related diabetes mellitus
ICD-10: E13	Other specified diabetes mellitus
ICD-10: E14	Unspecified diabetes mellitus
ATC: A10	Drugs used in diabetes
HbA1c > 48 mmol/L	Laboratory value

For peer review only

For peer review only - http://bmjopen.bmj.com/site/about/guidelines.xhtml

Table S3. Stages of chronic kidney disease diagnosed using measurements of eGFR and UACR according to the KDIGO clinical practice guidelines

**eGFR-defined stages**		
**Stage**	**eGFR thresholds (mL.min.1.73m²)**	**Abnormal**
1	≥ 90	No
2	60-89	No
3a	45-59	Yes
3b	30-44	Yes
4	15-29	Yes
5	GFR < 15	Yes
**Albuminuria-defined stages**		
**Stage**	**UACR thresholds (g/mol)**	**Abnormal**
A1	UACR < 3	No
A2	UACR 3-30	Yes
A3	UACR > 30	Yes

eGFR denotes estimated glomerular filtration rate; UACR, urinary albumin-creatinine ratio.

For peer review only - http://bmjopen.bmj.com/site/about/guidelines.xhtml

Table S4. International Classification of Diseases (ICD)-10 codes used to search for diagnoses of CKD, prevalent comorbidities, and outcomes of interest

Condition	ICD-10 codes
**CKD diagnosis and exclusions**	
CKD (broad)	N17-N19, I12.0, I12.9, I13.1-I13.2, E10.2, E11.2, E12.2, E13.2, E14.2, N00-N08, N10-N16, Z49, Z99.2
CKD (narrow)	N17-N19, I12.0, I12.9, I13.1-I13.2, E10.2, E11.2, E12.2, E13.2, E14.2, N083, Z49.1-Z49.2, Z99.2
Kidney donation	Z52.4
**Comorbidities**	
Heart failure	I50, I11.0, I13.0, I13.2
Coronary heart disease	I20-I25
Acute myocardial infarction	I21
Unstable angina	I20.0
Stroke	I60-I66, G45
Atrial fibrillation	I48
Peripheral artery disease	I70-I72, I73.1, I73.9, I74, I77.3, I77.6, I77.8, I79
Diabetes	E10-E14
Cancer	C
Head and neck cancer	C00-C14
Digestive organs cancer	C15-C26
Lung and thorax cancer	C30-C39
Skin cancer	C43-C44
Breast cancer	C50
Female genital cancer	C51-C58
Male genital cancer	C61
Urinary cancer	C64-C68
Hematologic cancer	C81-C96
**Outcomes**	
CVD	I
MACE	I50, I11.0, I13.0, I13.2, I42, I43, I25.5, I21, I60-I62, I63.0-I63.5, I63.8-I63.9, I64

CKD denotes chronic kidney disease; CVD, cardiovascular disease; MACE, major cardiovascular events. ICD-10 codes for “CKD (broad)” were used to search for diagnoses of CKD prior to the index date, which would exclude them from the analyses. ICD-10 codes for “CKD (narrow) were used to search for diagnoses of CKD during follow-up.

For peer review only

For peer review only - http://bmjopen.bmj.com/site/about/guidelines.xhtml

Table S5. Anatomical Therapeutic Chemical (ATC) classification system codes used to search for treatments of interest in this study's population

Treatment	ATC codes
RAASi	C09
Statin	C10AA, C10BA01-C10BA09, C10BA11-C10BA12, C10BX
SGLT-2i	A10BK01, A10BK02, A10BK03, A10BD15, A10BD16, A10BD20

RAASi denotes renin angiotensin aldosterone system inhibitors; SGLT-2i, sodium-glucose cotransporter-2 inhibitors.

For peer review only

For peer review only - http://bmjopen.bmj.com/site/about/guidelines.xhtml

Table S6. Baseline characteristics of the total study cohort stratified by age group

	18-45 years	45-65 years	65-80 years	80+ years
**N**	1,441	9,955	47,319	40,667
**Female, N (%)**	706 (49.0%)	3,830 (38.5%)	22,143 (46.8%)	24,519 (60.3%)
**Age, median (IQR)**	37.8 (30.9-41.9)	59.6 (55.0-62.7)	74.2 (70.8-77.1)	85.5 (82.6-89.1)
**Socioeconomic status, N (%)**				
Missing	776 (53.9%)	5,898 (59.2%)	30,480 (64.4%)	27,947 (68.7%)
High	90 (13.5%)	673 (16.6%)	3,743 (22.2%)	2,848 (22.4%)
Middle	216 (32.5%)	1,510 (37.2%)	7,345 (43.6%)	5,083 (40.0%)
Low	359 (54.0%)	1,874 (46.2%)	5,751 (34.2%)	4,789 (37.6%)
**Comorbidities, N (%)**				
Heart failure	45 (3.1%)	1,173 (11.8%)	7,982 (16.9%)	11,090 (27.3%)
CHD	20 (1.4%)	1,574 (15.8%)	12,268 (25.9%)	12,749 (31.3%)
AMI	9 (0.6%)	772 (7.8%)	5,463 (11.5%)	5,041 (12.4%)
Unstable angina	<=5	357 (3.6%)	2,860 (6.0%)	2,279 (5.6%)
Stroke	31 (2.2%)	828 (8.3%)	7,181 (15.2%)	8,677 (21.3%)
Atrial fibrillation	24 (1.7%)	1,038 (10.4%)	11,049 (23.4%)	13,263 (32.6%)
PAD	31 (2.2%)	510 (5.1%)	4,637 (9.8%)	3,975 (9.8%)
Diabetes	775 (53.8%)	5,031 (50.5%)	16,545 (35.0%)	9,394 (23.1%)
Cancer	58 (4.0%)	1,516 (15.2%)	14,615 (30.9%)	14,628 (36.0%)
Head and neck	6 (0.4%)	53 (0.5%)	396 (0.8%)	347 (0.9%)
Digestive	<=5	197 (2.0%)	1,739 (3.7%)	1,786 (4.4%)
Lung and thorax	<=5	112 (1.1%)	880 (1.9%)	455 (1.1%)
Skin	<=5	313 (3.1%)	5,123 (10.8%)	7,991 (19.6%)
Breast	<=5	185 (1.9%)	2,147 (4.5%)	1,836 (4.5%)
Female genital	<=5	119 (1.2%)	884 (1.9%)	778 (1.9%)
Male genital	<=5	160 (1.6%)	3,253 (6.9%)	2,397 (5.9%)
Urinary	10 (0.7%)	283 (2.8%)	1,653 (3.5%)	1,066 (2.6%)
Hematologic	13 (0.9%)	216 (2.2%)	1,215 (2.6%)	787 (1.9%)
**Measurements, median (IQR)**				
Systolic BP (mmHg)	130.0 (120.0-140.0)	137.0 (125.0-150.0)	140.0 (126.0-150.0)	140.0 (129.0-154.5)
BMI (kg/m²)	28.8 (24.3-34.4)	29.3 (25.7-33.4)	27.1 (24.2-30.7)	24.8 (22.2-28.0)
**Laboratory, median (IQR)**				
P-creatinine (μmol/L)	71.0 (57.0-94.0)	94.0 (72.0-115.0)	97.0 (84.0-109.0)	90.0 (78.0-105.0)
eGFR (mL.min.1.73m²)	94.2 (74.0-107.0)	59.2 (53.6-83.8)	54.6 (49.3-58.2)	50.5 (42.8-55.8)
UACR (g/mol)	9.0 (4.7-24.0)	6.4 (3.6-15.2)	3.3 (0.7-8.1)	2.5 (0.7-6.9)
Sodium (mmol/L)	139.0 (138.0-141.0)	140.0 (138.0-141.0)	140.0 (138.0-142.0)	140.0 (138.0-142.0)
Potassium (mmol/L)	4.0 (3.8-4.3)	4.2 (3.9-4.4)	4.2 (4.0-4.4)	4.2 (3.9-4.4)
HbA1c (mmol/mol)	54.0 (40.0-73.0)	50.0 (40.0-65.0)	44.0 (39.0-54.0)	42.0 (38.0-51.0)
Hemoglobin (g/L)	139.5 (126.0-152.0)	140.0 (129.0-152.0)	136.0 (126.0-146.0)	131.0 (120.0-141.0)
**Medications, N (%)**				
Statins	234 (16.2%)	4,012 (40.3%)	22,233 (47.0%)	14,253 (35.0%)
RAASi	452 (31.4%)	5,952 (59.8%)	29,472 (62.3%)	20,894 (51.4%)
SGLT2i	26 (1.8%)	335 (3.4%)	547 (1.2%)	78 (0.2%)

For peer review only - http://bmjopen.bmj.com/site/about/guidelines.xhtml

Data for baseline demographics and prevalent comorbidities were collected for each patient on the date this study identified their CKD (index date) using eGFR and UACR measurements according to the KDIGO clinical practice guidelines. The most recent clinical measurements and laboratory values within the one year prior to the index date are reported. For patients residing in Region Stockholm, the most recent socioeconomic data within the 10 years prior to the index date was collected from the Mosaic system, which applies the principles of geodemography to consumer household and individual data to categorize patients into one of the following three levels: 1) Highest income/education; 2) Medium income/education; and 3) Lowest income/education. AMI denotes acute myocardial infarction; BMI, body mass index; BP, blood pressure; CHD, coronary heart disease; eGFR, estimated glomerular filtration rate; HbA1c, glycated hemoglobin; IQR, interquartile range; PAD, peripheral artery disease; RAASi, renin-angiotensin aldosterone system inhibitors; SGLT2i, sodium-glucose cotransporter-2 inhibitors; UACR, urinary albumin-creatinine ratio.

For peer review only

For peer review only - http://bmjopen.bmj.com/site/about/guidelines.xhtml

Table S7. Proportions of patients diagnosed with chronic kidney disease in healthcare within five years of the development of the disease

Stage of CKD	Percentage with a diagnosis in healthcare within 5 years
S3a	29%
S3b	59%
S4+S5	82%
A2	34%
A3	62%

Each stage of chronic kidney disease (CKD) is defined using measurements of estimated glomerular filtration rate (stages S3a, S3b, and S4+S5) and urinary albumin-creatinine ratio (stages A2 and A3) according to the KDIGO clinical practice guidelines.

For peer review only

For peer review only - http://bmjopen.bmj.com/site/about/guidelines.xhtml

Figure S1. Participant flow

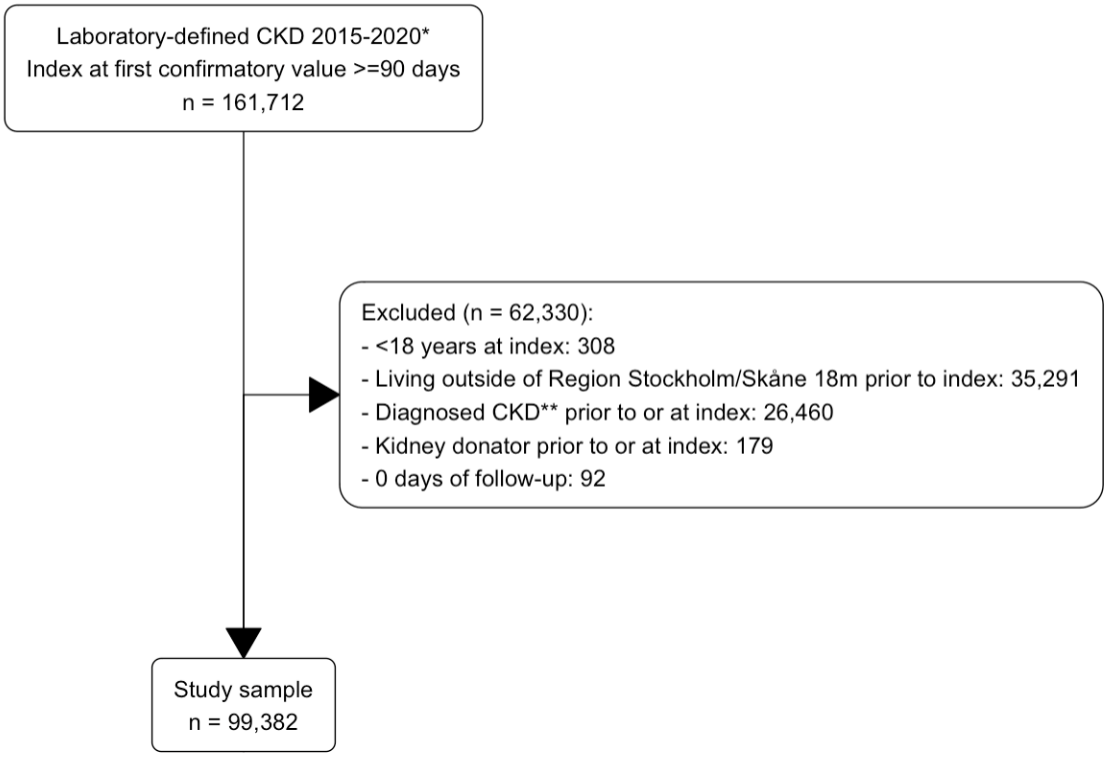

*Laboratory-defined CKD = KDIGO confirmed definition; **Diagnosed CKD = Any diagnosis position, any care level.

For peer review only - http://bmjopen.bmj.com/site/about/guidelines.xhtml

Figure S2. Multi-state models presenting the proportions of patients diagnosed or not diagnosed with chronic kidney disease (CKD) in healthcare who received or didn't receive guideline-directed pharmacological therapy, including the terminal, absorbing outcome, all-cause death

A) Statins

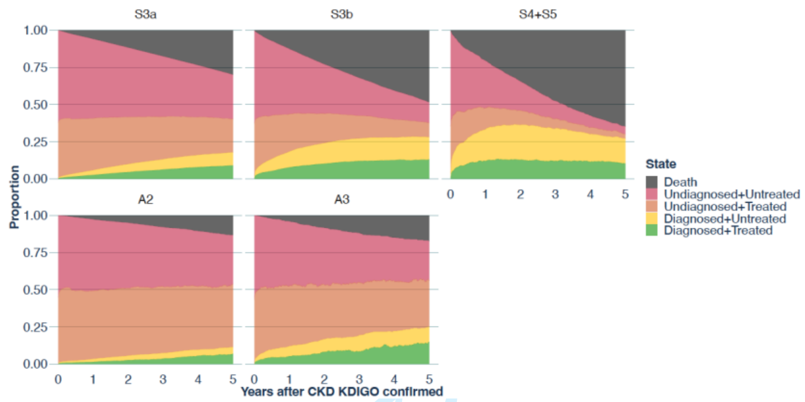

B) RAASi

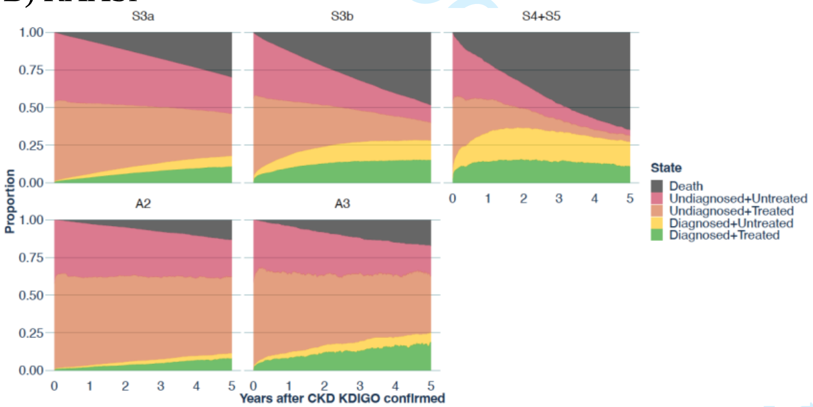

C) SGLT2i

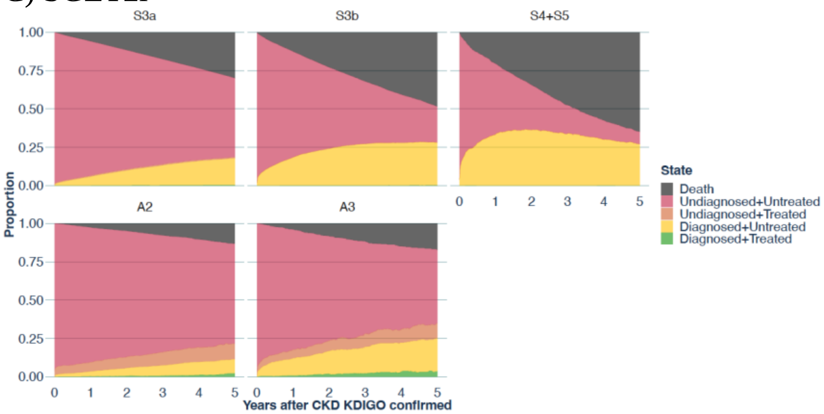

Use of A) Statins, B) renin-angiotensin aldosterone system inhibitors (RAASi), and C) sodium-glucose cotransporter-2 inhibitors (SGLT2i) were assessed. Patients are stratified by the KDIGO-defined stage of their CKD, identified using estimated glomerular filtration rate (Stages S3a, S3b, and S4+S5) and urinary albumin-creatinine ratio (Stages A2 and A3).

For peer review only - http://bmjopen.bmj.com/site/about/guidelines.xhtml

Figure S3. Multi-state models presenting the proportions of patients diagnosed or not diagnosed with chronic kidney disease (CKD) in healthcare who received or didn't receive guideline-directed pharmacological therapy, including the terminal, absorbing outcomes, major adverse cardiovascular events (MACE) and non-cardiovascular disease (CVD) death

A) Statins

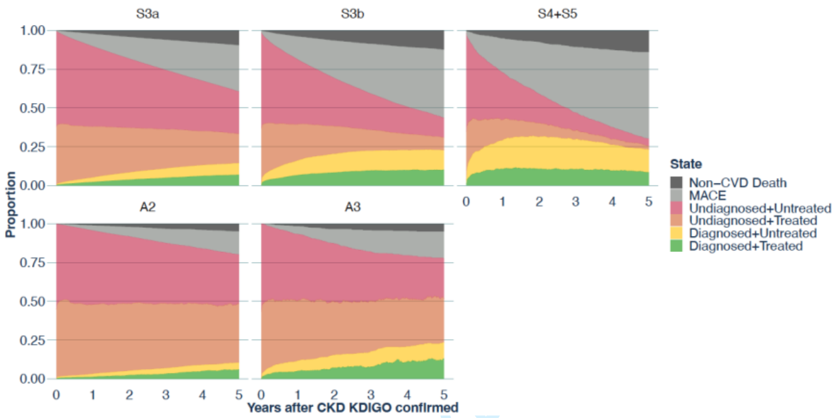

B) RAASi

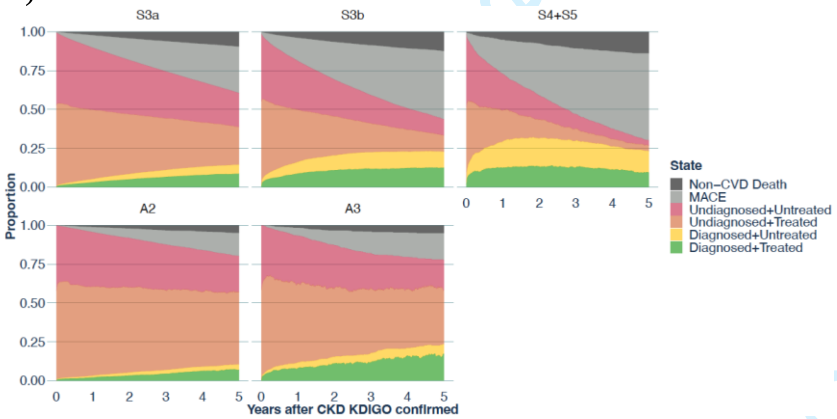

C) SGLT2i

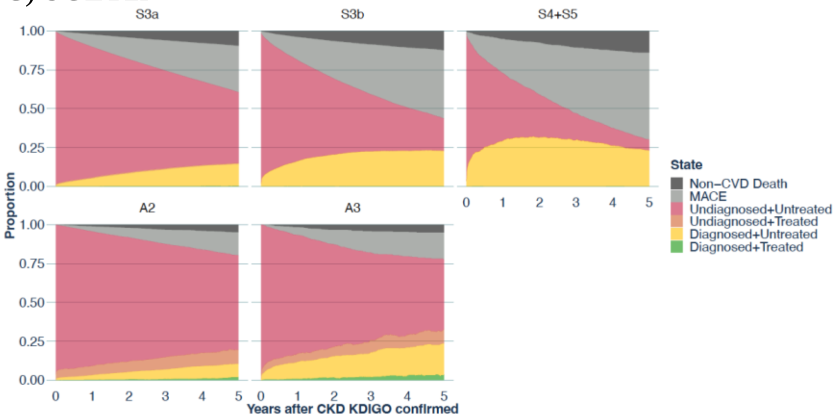

Use of A) Statins, B) renin-angiotensin aldosterone system inhibitors (RAASi), and C) sodium-glucose cotransporter-2 inhibitors (SGLT2i) were assessed. Patients are stratified by the KDIGO-defined stage of their CKD, identified using estimated glomerular filtration rate (Stages S3a, S3b, and S4+S5) and urinary albumin-creatinine ratio (Stages A2 and A3).

For peer review only - http://bmjopen.bmj.com/site/about/guidelines.xhtml

Figure S4. Multi-state models presenting the proportions of patients diagnosed or not diagnosed with chronic kidney disease (CKD) in healthcare who received or didn't receive guideline-directed pharmacological therapy, including the terminal, absorbing outcome, all-cause death

A) Statins

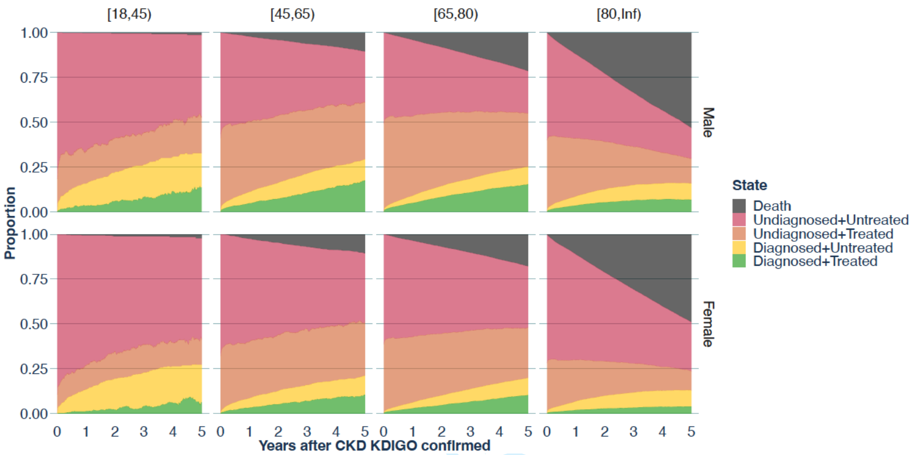

B) RAASi

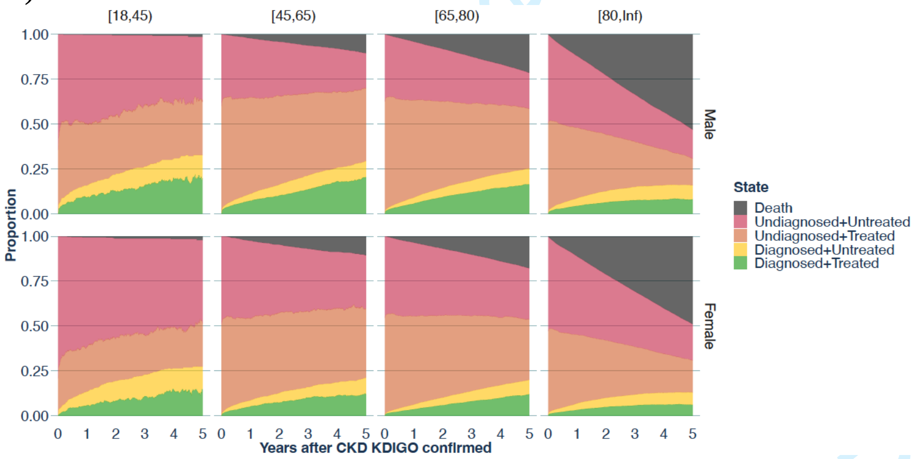

C) SGLT2i

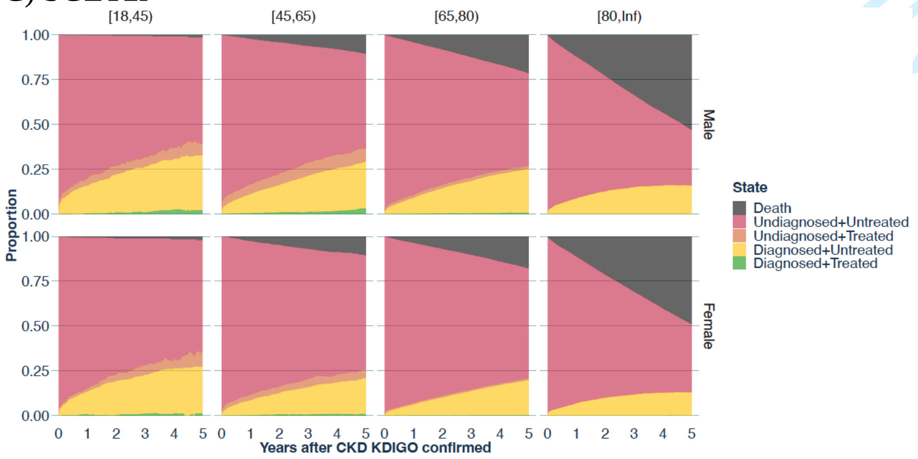

Use of A) Statins, B) renin-angiotensin aldosterone system inhibitors (RAASi), and C) sodium-glucose cotransporter-2 inhibitors (SGLT2i) were assessed. Patients are stratified by gender and age group.

For peer review only - http://bmjopen.bmj.com/site/about/guidelines.xhtml

Figure S5. Multi-state models presenting the proportions of patients diagnosed or not diagnosed with chronic kidney disease (CKD) in healthcare who received or didn't receive guideline-directed pharmacological therapy, including the terminal, absorbing outcomes, major adverse cardiovascular events (MACE) and non-cardiovascular disease (CVD) death

A) Statins

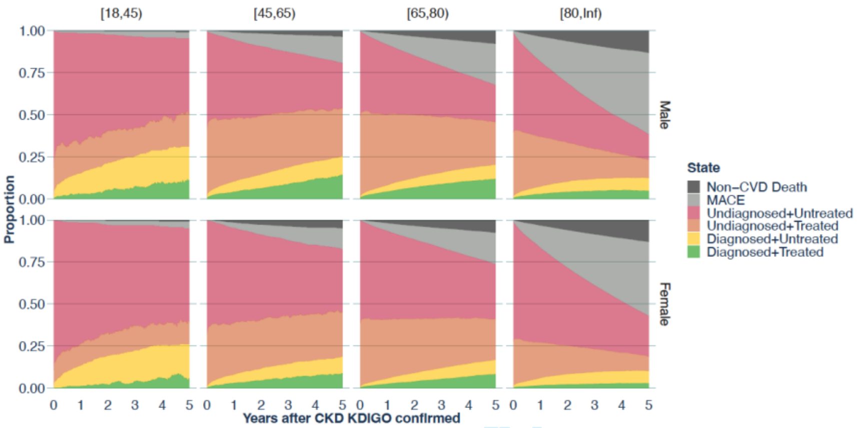

B) RAASi

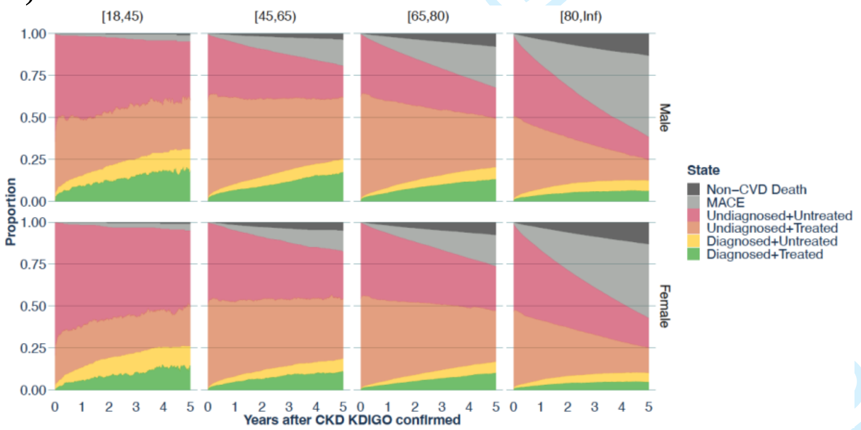

C) SLGT2i

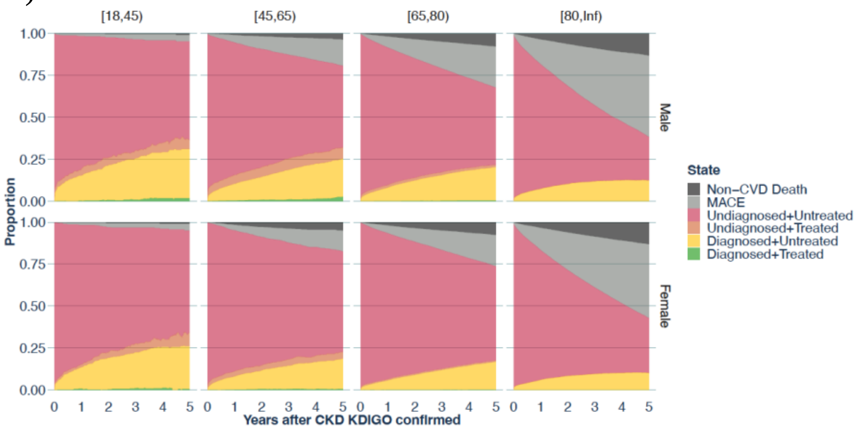

Use of A) Statins, B) renin-angiotensin aldosterone system inhibitors (RAASi), and C) sodium-glucose cotransporter-2 inhibitors (SGLT2i) were assessed. Patients are stratified by gender and age group.

For peer review only - http://bmjopen.bmj.com/site/about/guidelines.xhtml

References

1. WHO Collaborating Centre for Drug Statistics Methodology. ATC/DDD Index 2017. https://www.whocc.no/.
2. WHO. Classifications. http://www.who.int/classifications/icd/en/.
3. (NOMESCO), N. M.-S. C. NOMESCO Classification of Surgical Procedures. https://norden.diva-portal.org/smash/get/diva2:970547/FULLTEXT01.pdf (2010).
4. Ludvigsson, J. F., Otterblad-Olausson, P., Pettersson, B. U. & Ekbom, A. The Swedish personal identity number: Possibilities and pitfalls in healthcare and medical research. *European Journal of Epidemiology* **24**, 659–667 (2009).
5. Qin, Y. *et al.* The effect of angiotensin-converting enzyme inhibitor/angiotensin receptor blocker use on mortality in patients with chronic kidney disease: a meta-analysis of observational studies. *Pharmacoepidemiol Drug Saf* **25**, 503–511 (2016).
6. Balamuthusamy, S. *et al.* Renin angiotensin system blockade and cardiovascular outcomes in patients with chronic kidney disease and proteinuria: a meta-analysis. *Am Heart J* **155**, 791–805 (2008).
7. Xie, X. *et al.* Renin-Angiotensin System Inhibitors and Kidney and Cardiovascular Outcomes in Patients With CKD: A Bayesian Network Meta-analysis of Randomized Clinical Trials. *American Journal of Kidney Diseases* **67**, 728–741 (2016).
8. Zhang, Y. *et al.* ACE Inhibitor Benefit to Kidney and Cardiovascular Outcomes for Patients with Non-Dialysis Chronic Kidney Disease Stages 3–5: A Network Meta-Analysis of Randomised Clinical Trials. *Drugs* **80**, 797–811 (2020).
9. Collaboration, B. P. L. T. T. Blood pressure lowering and major cardiovascular events in people with and without chronic kidney disease: meta-analysis of randomised controlled trials. *BMJ* **347**, f5680 (2013).
10. Major, R. W., Cheung, C. K., Gray, L. J. & Brunskill, N. J. Statins and Cardiovascular Primary Prevention in CKD: A Meta-Analysis. *Clin J Am Soc Nephrol* **10**, 732–739 (2015).
11. Su, X. *et al.* Effect of Statins on Kidney Disease Outcomes: A Systematic Review and Meta-analysis. *American Journal of Kidney Diseases* **67**, 881–892 (2016).
12. Upadhyay, A. *et al.* Lipid-lowering therapy in persons with chronic kidney disease: a systematic review and meta-analysis. *Ann Intern Med* **157**, 251–262 (2012).
13. Strippoli, G. F. M. *et al.* Effects of statins in patients with chronic kidney disease: meta-analysis and meta-regression of randomised controlled trials. *BMJ* **336**, 645–651 (2008).
14. Palmer, S. C. *et al.* Benefits and harms of statin therapy for persons with chronic kidney disease: a systematic review and meta-analysis. *Ann Intern Med* **157**, 263–275 (2012).
15. Hou, W. *et al.* Effect of statin therapy on cardiovascular and renal outcomes in patients with chronic kidney disease: a systematic review and meta-analysis. *Eur Heart J* **34**, 1807–1817 (2013).
16. Zhang, X. *et al.* Effect of statins on cardiovascular events in patients with mild to moderate chronic kidney disease: a systematic review and meta-analysis of randomized clinical trials. *BMC Cardiovasc Disord* **14**, 19 (2014).
17. Barylski, M. *et al.* Statins decrease all-cause mortality only in CKD patients not requiring dialysis therapy—A meta-analysis of 11 randomized controlled trials involving 21,295 participants. *Pharmacological Research* **72**, 35–44 (2013).
18. Yamada, T. *et al.* Cardiovascular and renal outcomes with SGLT-2 inhibitors versus GLP-1 receptor agonists in patients with type 2 diabetes mellitus and chronic kidney disease: a systematic review and network meta-analysis. *Cardiovascular Diabetology* **20**, 14 (2021).

For peer review only - http://bmjopen.bmj.com/site/about/guidelines.xhtml

19. Salah, H. M. *et al.* Effect of sodium-glucose cotransporter 2 inhibitors on cardiovascular and kidney outcomes-Systematic review and meta-analysis of randomized placebo-controlled trials. *Am Heart J* **232**, 10–22 (2021).
20. Lin, D. S.-H., Lee, J.-K., Hung, C.-S. & Chen, W.-J. The efficacy and safety of novel classes of glucose-lowering drugs for cardiovascular outcomes: a network meta-analysis of randomised clinical trials. *Diabetologia* **64**, 2676–2686 (2021).
21. Kamdar, A., Sykes, R., Morrow, A., Mangion, K. & Berry, C. Cardiovascular outcomes of glucose lowering therapy in chronic kidney disease patients: a systematic review with meta-analysis. *Rev Cardiovasc Med* **22**, 1479–1490 (2021).
22. D'Andrea, E. *et al.* Heterogeneity of antidiabetic treatment effect on the risk of major adverse cardiovascular events in type 2 diabetes: a systematic review and meta-analysis. *Cardiovasc Diabetol* **19**, 154 (2020).
23. Staplin, N. *et al.* Net effects of sodium-glucose co-transporter-2 inhibition in different patient groups: a meta-analysis of large placebo-controlled randomized trials. *EClinicalMedicine* **41**, 101163 (2021).
24. Malik, A. H. *et al.* Cardiovascular Outcomes With the Use of Sodium-Glucose Cotransporter-2 Inhibitors in Patients With Type 2 Diabetes and Chronic Kidney Disease: An Updated Meta-Analysis of Randomized Controlled Trials. *Cardiol Rev* **28**, 116–124 (2020).
25. Cao, H. *et al.* Sodium-glucose cotransporter 2 inhibitors benefit to kidney and cardiovascular outcomes for patients with type 2 diabetes mellitus and chronic kidney disease 3b-4: A systematic review and meta-analysis of randomized clinical trials. *Diabetes Research and Clinical Practice* **180**, (2021).
26. Kaze, A. D., Zhuo, M., Kim, S. C., Patorno, E. & Paik, J. M. Association of SGLT2 inhibitors with cardiovascular, kidney, and safety outcomes among patients with diabetic kidney disease: a meta-analysis. *Cardiovascular Diabetology* **21**, 47 (2022).
27. Yang, S., Zhao, L., Mi, Y. & He, W. Effects of sodium-glucose cotransporter-2 inhibitors and aldosterone antagonists, in addition to renin-angiotensin system antagonists, on major adverse kidney outcomes in patients with type 2 diabetes and chronic kidney disease: A systematic review and network meta-analysis. *Diabetes, Obesity and Metabolism* **n/a**,.

For peer review only

For peer review only - http://bmjopen.bmj.com/site/about/guidelines.xhtml

STROBE 2007 (v4) Statement—Checklist of items that should be included in reports of *cohort studies*

Section/Topic	Item #	Recommendation	Reported on page #
**Title and abstract**	1	(*a*) Indicate the study's design with a commonly used term in the title or the abstract	1
		(*b*) Provide in the abstract an informative and balanced summary of what was done and what was found	2
**Introduction**			
Background/rationale	2	Explain the scientific background and rationale for the investigation being reported	4
Objectives	3	State specific objectives, including any prespecified hypotheses	4
**Methods**			
Study design	4	Present key elements of study design early in the paper	4
Setting	5	Describe the setting, locations, and relevant dates, including periods of recruitment, exposure, follow-up, and data collection	4
Participants	6	(*a*) Give the eligibility criteria, and the sources and methods of selection of participants. Describe methods of follow-up	5
		(*b*) For matched studies, give matching criteria and number of exposed and unexposed	-
Variables	7	Clearly define all outcomes, exposures, predictors, potential confounders, and effect modifiers. Give diagnostic criteria, if applicable	5-6
Data sources/ measurement	8*	For each variable of interest, give sources of data and details of methods of assessment (measurement). Describe comparability of assessment methods if there is more than one group	5-6
Bias	9	Describe any efforts to address potential sources of bias	-
Study size	10	Explain how the study size was arrived at	-
Quantitative variables	11	Explain how quantitative variables were handled in the analyses. If applicable, describe which groupings were chosen and why	6-7
Statistical methods	12	(*a*) Describe all statistical methods, including those used to control for confounding	6-7
		(*b*) Describe any methods used to examine subgroups and interactions	6-7
		(*c*) Explain how missing data were addressed	6-7
		(*d*) If applicable, explain how loss to follow-up was addressed	-
		(*e*) Describe any sensitivity analyses	-
**Results**			

For peer review only

Participants	13*	(a) Report numbers of individuals at each stage of study—eg numbers potentially eligible, examined for eligibility, confirmed eligible, included in the study, completing follow-up, and analysed	7
		(b) Give reasons for non-participation at each stage	-
		(c) Consider use of a flow diagram	Figure S1 Supplement, page 12
Descriptive data	14*	(a) Give characteristics of study participants (eg demographic, clinical, social) and information on exposures and potential confounders	8 (Table 1)
		(b) Indicate number of participants with missing data for each variable of interest	8
		(c) Summarise follow-up time (eg, average and total amount)	7
Outcome data	15*	Report numbers of outcome events or summary measures over time	10
Main results	16	(*a*) Give unadjusted estimates and, if applicable, confounder-adjusted estimates and their precision (eg, 95% confidence interval). Make clear which confounders were adjusted for and why they were included	10
		(*b*) Report category boundaries when continuous variables were categorized	10
		(*c*) If relevant, consider translating estimates of relative risk into absolute risk for a meaningful time period	10
Other analyses	17	Report other analyses done—eg analyses of subgroups and interactions, and sensitivity analyses	10
**Discussion**			
Key results	18	Summarise key results with reference to study objectives	13
**Limitations**			
Interpretation	20	Give a cautious overall interpretation of results considering objectives, limitations, multiplicity of analyses, results from similar studies, and other relevant evidence	14-15
Generalisability	21	Discuss the generalisability (external validity) of the study results	15
**Other information**			
Funding	22	Give the source of funding and the role of the funders for the present study and, if applicable, for the original study on which the present article is based	16

*Give information separately for cases and controls in case-control studies and, if applicable, for exposed and unexposed groups in cohort and cross-sectional studies.

Note: An Explanation and Elaboration article discusses each checklist item and gives methodological background and published examples of transparent reporting. The STROBE checklist is best used in conjunction with this article (freely available on the Web sites of PLoS Medicine at http://www.plosmedicine.org/, Annals of Internal Medicine at http://www.annals.org/, and Epidemiology at http://www.epidem.com/). Information on the STROBE Initiative is available at www.strobe-statement.org.

For peer review only
